# Light-Promoted C–H/C–H Coupling of Imidazo[1,2-*a*]pyridines with 5-(Hetero)aryl-1,2,5-oxadiazolo[3,4-*b*]pyrazines over TiO_2_ and Experimental/In Silico Evaluation of COX-1 and COX-2 Inhibitory Activity

**DOI:** 10.3390/molecules31162830

**Published:** 2026-08-13

**Authors:** Maria A. Trestsova, Daria A. Andreeva, Mikhail A. Kiskin, Maria V. Komelkova, Pavel M. Vassiliev, Alena. S. Taran, Ludmila A. Yolshina, Alexander G. Kvashnichev, Veronika A. Isaeva, Irina A. Utepova, Oleg N. Chupakhin, Alexey P. Sarapultsev

**Affiliations:** 1 Department of Organic and Biomolecular Chemistry, Ural Federal University, 28 Mira Street, 620002 Ekaterinburg, Russia; mtrescova@mail.ru (M.A.T.); andreevadarya03@mail.ru (D.A.A.); chupakhin@ios.uran.ru (O.N.C.); 2 Institute of Organic Synthesis of the Russian Academy of Sciences, 22 S. Kovalevskoy Street, 620066 Ekaterinburg, Russia; 3 Russian-Chinese Education and Research Center of System Pathology, South Ural State University, 76 Lenin prosp., 454080 Chelyabinsk, Russia; mkomelkova@mail.ru (M.V.K.); pvassiliev@mail.ru (P.M.V.);; 4 Kurnakov Institute of General and Inorganic Chemistry of the Russian Academy of Sciences, 31 Leninsky prosp., 119991 Moscow, Russia; mkiskin@igic.ras.ru; 5 Scientific Center for Innovative Medicines, Volgograd State Medical University, 1 Pavshikh Bortsov Square, 400131 Volgograd, Russia; 6 Institute of High-Temperature Electrochemistry, Ural Branch of the Russian Academy of Sciences, 20 Akademicheskaya Street, 620066 Ekaterinburg, Russiakvashnichev@bk.ru (A.G.K.); 7 Institute of Immunology and Physiology, Ural Branch of the Russian Academy of Sciences, 106 Pervomaiskaya Street, 620049 Ekaterinburg, Russia

**Keywords:** C–H/C–H coupling, photocatalytic oxidation, titanium dioxide, imidazo[1,2-*a*]pyridine, oxadiazolopyrazine, heterogeneous photocatalysis, multiple docking, fully connected convolutional correlation neural network, COX inhibitors, fluorometric screening

## Abstract

A light-promoted C–H/C–H coupling of imidazo[1,2-*a*]pyridines with 5-(hetero)aryl-1,2,5-oxadiazolo[3,4-*b*]pyrazines was developed using a heterogeneous oxidative photocatalytic system based on molecular oxygen, nanosized TiO_2_, and light irradiation. The method provides direct access to C3-heteroarylated imidazo[1,2-*a*]pyridines under metal-free conditions and expands the synthetic utility of electron-deficient oxadiazolopyrazine partners in the construction of biheteroaryl scaffolds. The synthesized compounds were evaluated computationally using a fully connected convolutional correlation neural network based on multiple-docking energy spectra, which prioritized the series as potential COX-1 and COX-2 ligands. To test this prioritization experimentally, all 18 compounds were screened in fluorometric COX-1 and COX-2 inhibitor assays at 1 µM. Compound 3f emerged as a strong preliminary COX-1 hit at 1 µM (86.64 ± 5.18% inhibition), whereas 3h and 3l showed weaker COX-1 inhibition. No compound showed high or moderate COX-2 inhibition at the screening concentration; only weak COX-2 inhibitory signals were observed for several derivatives. Thus, the combined synthetic, computational, and enzymatic data identify compound 3f as the main COX-1-skewed hit in this series and provide a basis for further dose–response, selectivity, and cell-based anti-inflammatory studies. It should also be noted that the COX-1 inhibition assay used ovine COX-1, whereas the computational models were built on human COX-1 and COX-2 structures; this species difference is an additional reason to treat the in silico–experimental comparison as approximate.

## 1. Introduction

Direct dehydrogenative cross-coupling between two C–H fragments has become an increasingly important strategy for constructing C–C bonds, because it reduces the need for prefunctionalized substrates and improves the step and atom economy of synthetic sequences [1,2]. In heteroaromatic chemistry, this approach is particularly attractive, since biheteroaryl motifs are widely represented in medicinal chemistry, natural products, agrochemicals, and functional organic materials [3,4,5,6]. Nevertheless, direct C–H/C–H coupling between two heteroaromatic partners remains synthetically demanding, since it requires careful selection of the oxidizing system and reaction conditions.

**Figure 1:**
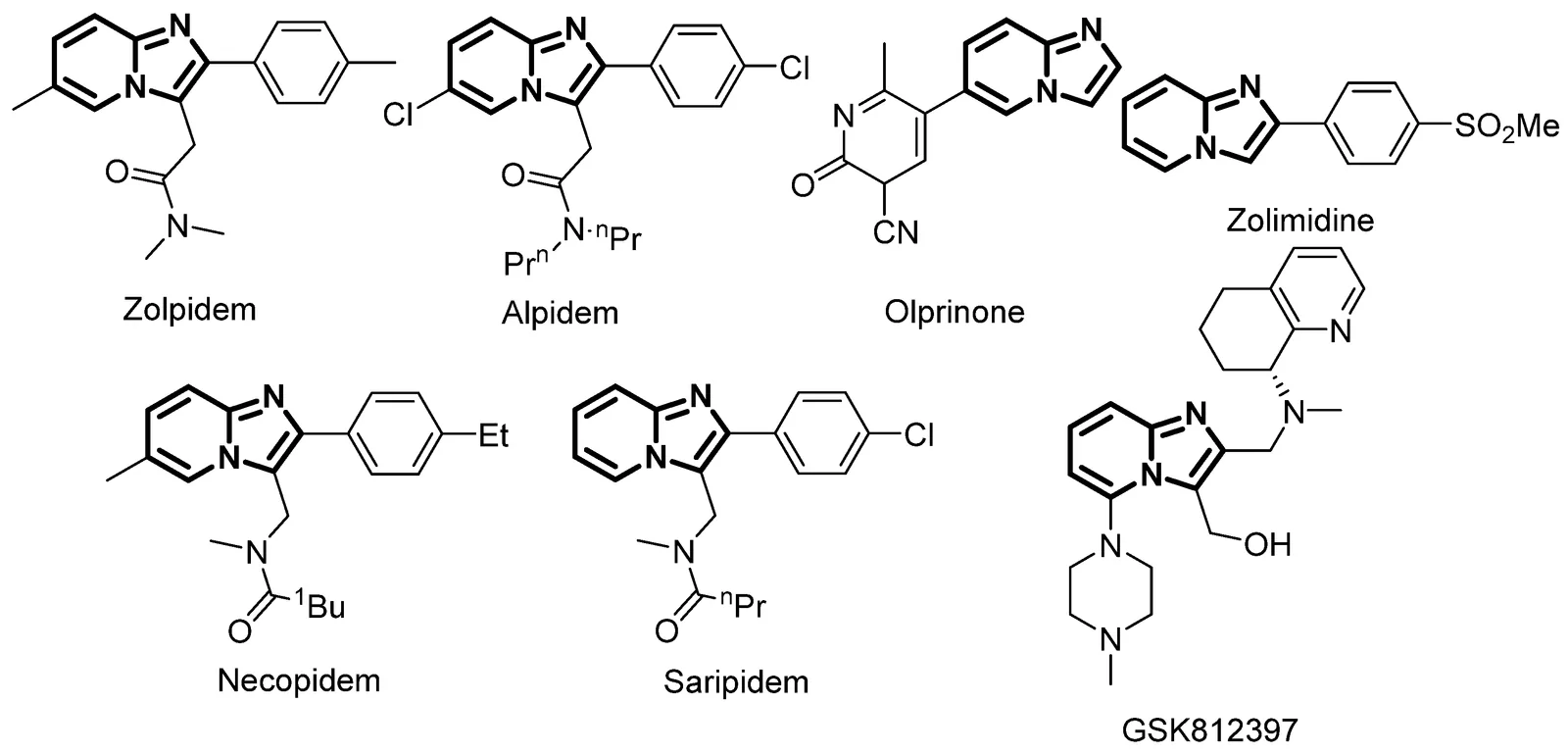
Imidazo[1,2-*a*]pyridine-based drugs.

The imidazo[1,2-*a*]pyridine scaffold is a privileged heteroaromatic motif in drug discovery. Derivatives of this core have been associated with antifungal, anti-inflammatory, antitumor, antiviral, antibacterial, antiprotozoal, antipyretic, and analgesic activities, and they also act as inhibitors of β-amyloid formation, as GABA and benzodiazepine-receptor ligands, and as cardiotonic agents [7,8,9,10,11,12]. Several clinically used or pharmacologically characterized compounds contain the imidazo[1,2-*a*]pyridine fragment, including zolpidem, alpidem, olprinone, zolimidine, necopidem, saripidem, and the HIV-related compound GSK812397 (Figure 1). This broad pharmacological relevance makes functionalization of imidazo[1,2-*a*]pyridines a valuable objective for both synthetic and medicinal chemistry.

Considerable effort has been devoted to the C3-functionalization of imidazo[1,2-*a*]pyridines; however, coupling with electron-deficient heteroarenes remains markedly less developed than reactions with electron-rich partners. In the present study, imidazo[1,2-*a*]pyridines were coupled with [1,2,5]oxadiazolo[3,4-*b*]pyrazines for the first time. The transformation was achieved using an oxidative system based on atmospheric oxygen, nanosized TiO_2_, and light irradiation. TiO_2_-based photocatalysis has been used in aerobic oxidative transformations of organic substrates, including selective oxidation of amines to imines and oxidation of heterocyclic substrates, while related TiO_2_-mediated systems have enabled oxidative coupling of azines with aromatic nucleophiles [13,14,15]. The present reaction extends this photocatalytic platform to a new class of C3-heteroarylated imidazo[1,2-*a*]pyridines.

In parallel with synthetic development, computational prioritization is increasingly used to select compounds for biological evaluation [16,17]. Fully connected neural networks [18] differ from other architectures in that they allow stable reference patterns to be constructed by energy minimization, whereas convolutional networks [19] are used to reduce the dimensionality of a redundant input space. Molecular docking remains a central tool in this field [20], but conventional site-directed docking usually estimates the affinity of a single ligand to one predefined binding site—a representation that can be restrictive, because ligand recognition may involve multiple regions of the protein surface rather than a single pocket. The present work therefore used a fully connected convolutional correlation neural network based on spectra of multiple-docking energies, while preserving the original docking and neural-network workflow described below.

Cyclooxygenase (COX) enzymes are biologically relevant targets for anti-inflammatory drug discovery, because they catalyze key steps of prostanoid biosynthesis [21]. COX-1 is constitutively expressed in most tissues and is linked to physiological prostanoid production, whereas COX-2 is strongly associated with the inducible generation of inflammatory prostaglandins, although constitutive COX-2 functions are also recognized in several tissues [22]. Screening novel heteroaromatic scaffolds against both isoforms is therefore informative not only for detecting activity, but also for identifying preliminary isoform-bias patterns that may guide subsequent optimization.

Here, we report a metal-free, TiO_2_-mediated C–H/C–H coupling route to 18 C3-heteroarylated imidazo[1,2-*a*]pyridines, evaluate their predicted affinity to COX-1 and COX-2 by a multiple-docking/FCCorNet workflow, and examine this computational prioritization by fluorometric in vitro COX-1 and COX-2 screening at 1 µM. This combined workflow links synthetic accessibility with enzymatic screening and identifies the most promising experimentally supported hit for subsequent medicinal-chemistry optimization.

## 2. Results and Discussion

### 2.1. Synthesis of the New Compounds

Optimization of the C–H/C–H coupling of imidazo[1,2-*a*]pyridines with [1,2,5]oxadiazolo[3,4-*b*]pyrazines was carried out using 2-phenylimidazo[1,2-*a*]pyridine (**1a**) and 5-phenyl-[1,2,5]oxadiazolo[3,4-*b*]pyrazine (**2a**) as model substrates (Table 1). Initially, the reaction was run in acetic acid using the oxidative system air O_2_/nanosized TiO_2_/Xe-lamp irradiation. At room temperature, the reaction did not proceed; however, upon refluxing the reaction mixture, the target product **3a** was obtained in 62% yield. Refluxing in acetic acid without the oxidative system afforded **3a** in only 8% yield. Replacing acetic acid with trifluoroacetic or trifluoromethanesulfonic acid, using boron trifluoride etherate (0.1 equiv) as a Lewis acid, or molecular iodine as a catalyst did not give the target compounds. Under otherwise identical conditions, commercial anatase TiO_2_ afforded **3a** in only 36% yield (Table 1, entry 10), whereas the rutile TiO_2_ nanopowder (10–20 nm) used throughout this work gave 62% (entry 3).

**Scheme 1:**
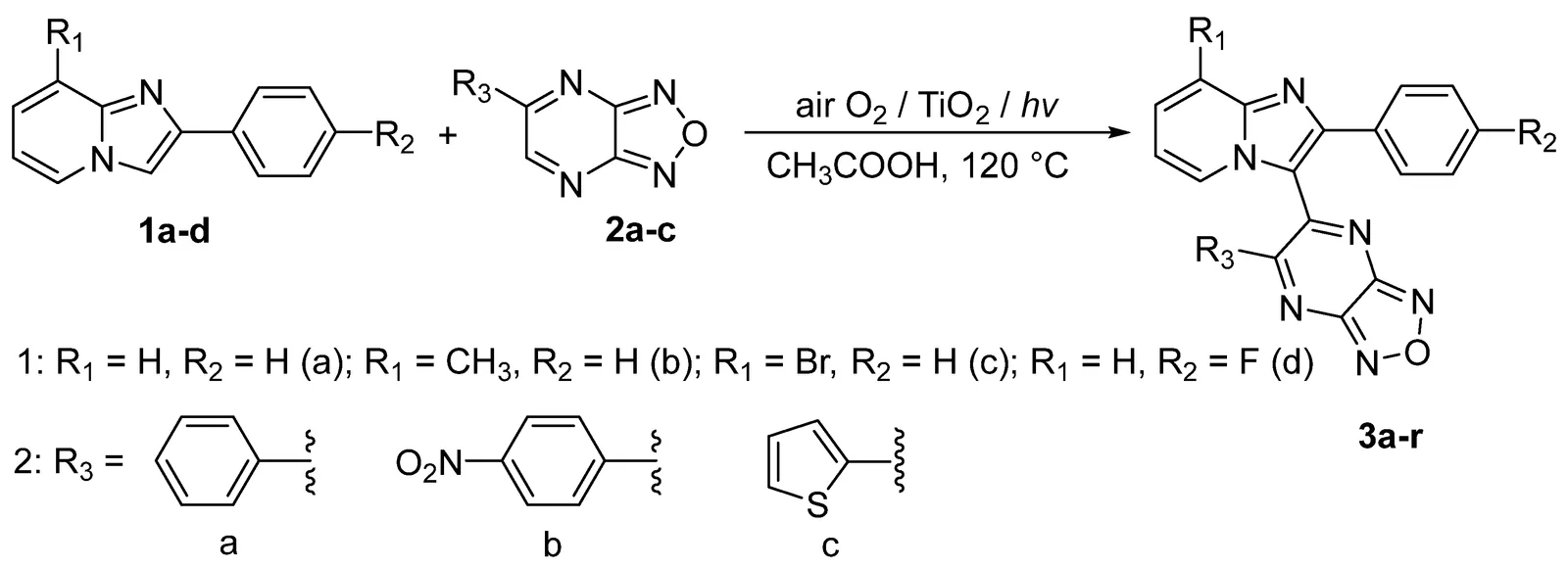
C–H/C–H coupling reactions of imidazo[1,2-*a*]pyridines **1a**–**f** with [1,2,5]oxadiazolo[3,4-*b*]pyrazines **2a**–**c**.

After optimizing the reaction conditions, we examined the applicability of the transformation to structurally diverse imidazo[1,2-*a*]pyridines and [1,2,5]oxadiazolo[3,4-*b*]pyrazines. The C–H/C–H coupling reactions of imidazo[1,2-*a*]pyridines **1a**–**f** with [1,2,5]oxadiazolo[3,4-*b*]pyrazines **2a**–**c** were carried out in the presence of the TiO_2_ photocatalyst under irradiation with a Xe lamp (5000 K, 35 W). To enhance the activity of TiO_2_ and to avoid coagulation of the nanosized particles, the mixture of starting materials was sonicated for 3 min. The reactions were run under reflux in acetic acid for 6 h (Scheme 1). The yields of the resulting compounds **3a**–**r** are summarized in Table 2. It should be noted that with other electron-deficient heterocycles, such as acridine, quinoxalin-2-one, 3,6-diphenyl-1,2,4-triazine, and quinazoline, the imidazo[1,2-*a*]pyridines did not react under the conditions described.

**Scheme 2:**
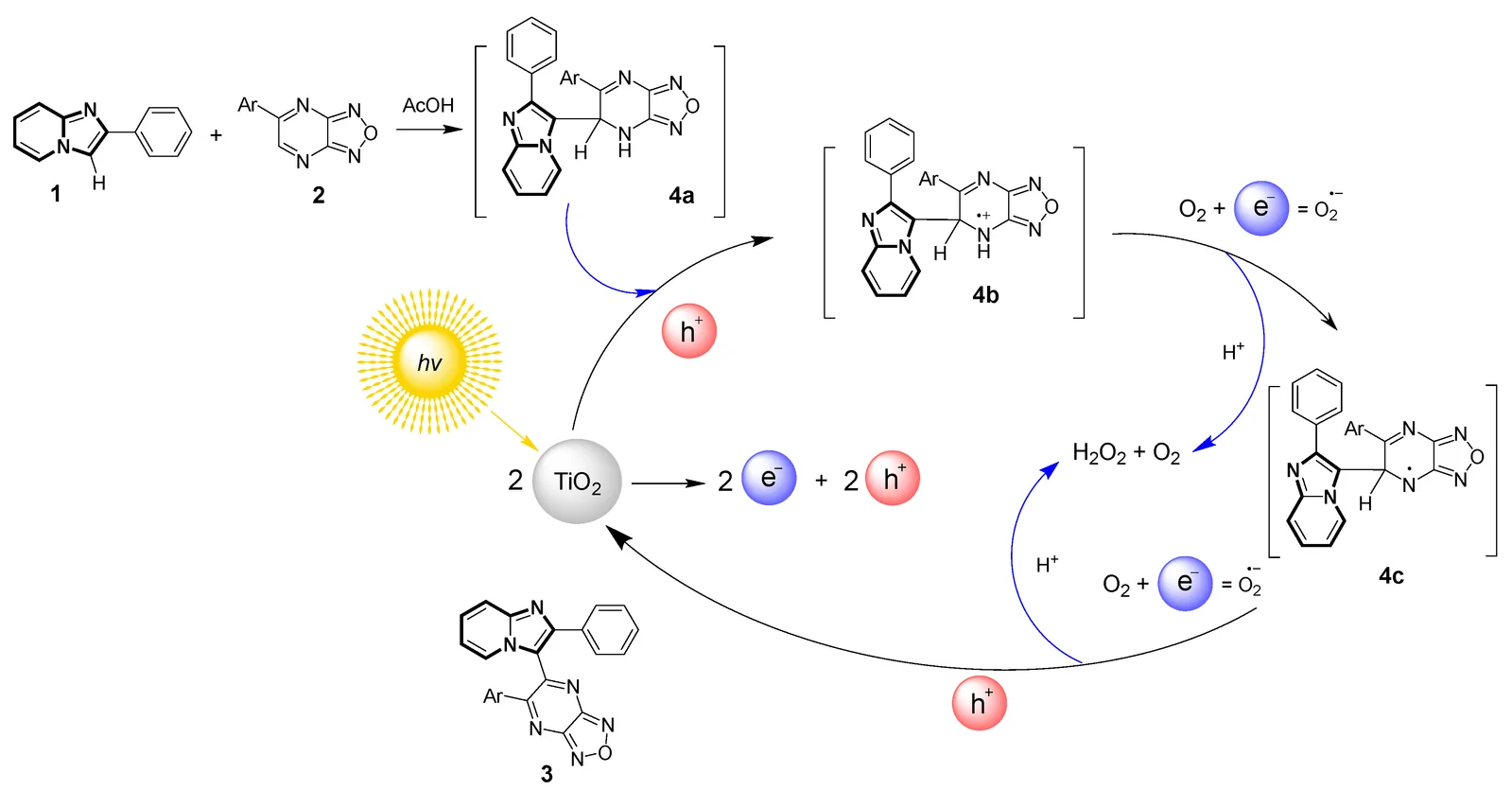
Proposed reaction mechanism.

On the basis of previous mechanistic studies of related TiO_2_-mediated aerobic oxidative C–H/C–H coupling reactions [13,23], a plausible mechanism is proposed for the coupling of imidazo[1,2-*a*]pyridines with [1,2,5]oxadiazolo[3,4-*b*]pyrazines (Scheme 2). Irradiation of the TiO_2_ surface can generate an e^−^/h^+^ pair, and atmospheric oxygen may interact with photogenerated electrons to form the superoxide radical anion O_2_^•−^. In the proposed pathway, the σ^H^-adduct **4a** may undergo single-electron oxidation to the radical-cation intermediate **4b**, followed by proton loss to give **4c**. Rearomatization to product **3** may then occur through subsequent oxidation and proton capture. Direct observation of the σ^H^-adduct, superoxide radical anion, and the individual oxidation/rearomatization steps was not performed for the present substrate pair; therefore, Scheme 2 should be regarded as a mechanistic proposal by analogy with earlier TiO_2_-mediated systems rather than as a directly proven pathway for this series.

**Figure 2:**
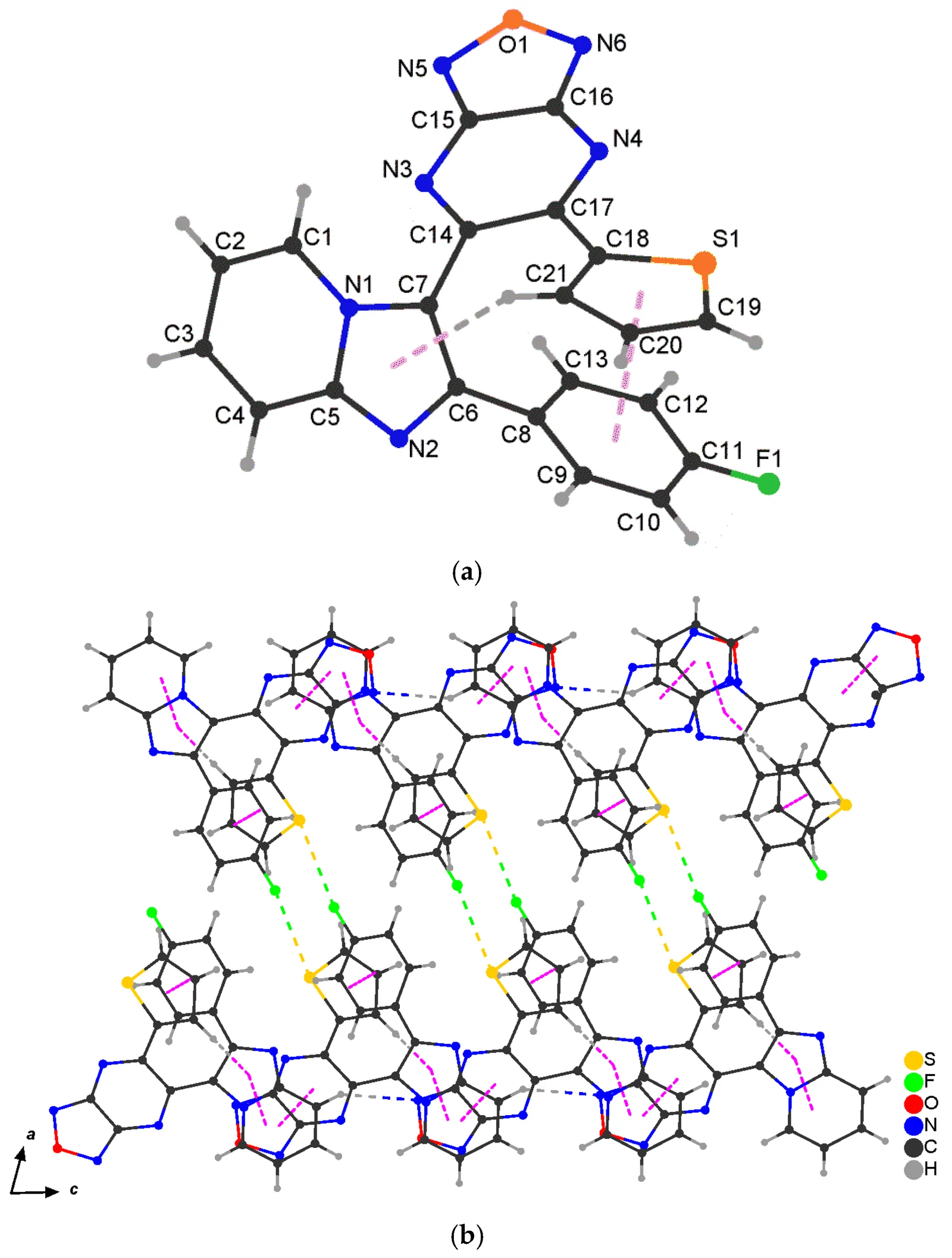
Molecular structure (**a**) and crystal packing of **3l** (non-covalent interactions C-H…N, S…F, C-H…π and π…π shown by dotted lines) (**b**).

The molecular structure of compound **3l**, 5-(2-(4-fluorophenyl)imidazo[1,2-*a*]pyridin-3-yl)-6-(thien-2-yl)[1,2,5]oxadiazolo[3,4-*b*]pyrazine, was confirmed by single-crystal X-ray diffraction analysis. In the molecule of **3l** (Figure 2a; selected bond distances and angles are listed in Table S1), the imidazopyridine and oxadiazolopyrazine fragments are rotated, and the dihedral angle N1-C7-C14-N3 is 47.8(2)o. The thiophene and oxadiazolopyrazine fragments are located at an angle N4-C17-C18-S1 of 28.0(2)o, and the angle between the phenyl and imidazopyridine fragment is slightly larger, N2-C6-C8-C9 42.0(3)o. In the molecule, π…π stacking interactions are formed between the thiophene and phenyl fragments, enhanced by the C-H…π contact of H atom of the thiophene ring and the imidazole ring (Tables S2 and S3). In the crystal, molecules form a supramolecular layer as a result of π…π contacts between the aromatic fragments of imidazopyridine and oxadiazolopyrazine and C-H…N interaction (Figure 2b, Tables S2 and S4). Additionally, weak contacts are observed between the fluorine and sulfur fragments of adjacent layers (S1…F1 3.129(3) Å), leading to their “dimerization”.

### 2.2. In Silico Affinity Evaluation of the New Compounds

Table 3 lists the calculated affinity descriptors of compounds **3a**–**r** toward COX-1.

The *W* values of the fully connected convolutional correlation neural network and the minimum multiple-docking energies (Δ*E*) listed in Table 3 indicate that the synthesized compounds constitute a computationally prioritized set of putative COX-1 ligands. Within this model, compound **3j** showed the most favorable minimum docking energy in the COX-1 series and therefore warranted particular attention in the subsequent experimental comparison. These computational descriptors were treated only as an *in silico* prioritization layer and were subsequently compared with direct enzymatic screening, rather than interpreted as evidence of inhibitory activity.

Table 4 lists the calculated affinity descriptors of compounds **3a**–**r** toward COX-2.

For COX-2, the synthesized compounds also showed favorable computational descriptors, and eight derivatives (**3k**, **3q**, **3b**, **3n**, **3e**, **3h**, **3d** and **3g**) had the most favorable minimum multiple-docking energy values (|ΔE| > 10.0 kcal/mol in this model). The mean minimum docking energy was somewhat more favorable for COX-2 (ΔEmean = −9.9 kcal/mol) than for COX-1 (ΔEmean = −8.7 kcal/mol), whereas the mean FCCorNet energy was higher for COX-1 (Wmean = 342) than for COX-2 (Wmean = 279). This divergence between the two computational descriptors indicates that they may capture different aspects of ligand–protein recognition; it does not, by itself, establish isoform-selective inhibition. Direct experimental validation is therefore required before any selectivity or mechanistic interpretation can be assigned, and such validation is reported in the following section.

### 2.3. In Vitro COX-1 and COX-2 Inhibitory Screening

To test the computational prioritization experimentally, all 18 synthesized compounds were screened for COX-1 and COX-2 inhibition at a single concentration of 1 µM using fluorometric inhibitor-screening assays, with SC-560 and celecoxib as COX-1- and COX-2-selective reference inhibitors, respectively. The screening results are summarized in Table 5. Because the assays were performed at a single concentration, the results are interpreted as preliminary screening data rather than as potency or selectivity estimates.

Under these screening conditions, the measurable experimental response was concentrated in the COX-1 assay, and only a small number of compounds showed inhibition. Compound **3f** produced strong apparent COX-1 inhibition at 1 µM (86.64 ± 5.18%), comparable to the SC-560 reference values on the assay plates, whereas 3h and 3l showed weaker COX-1 inhibition (31.05 ± 8.34% and 15.91 ± 11.20%, respectively). None of the compounds reached high or moderate COX-2 inhibition at 1 µM; only weak COX-2 signals (approximately 10–17%) were detected for **3r**, **3e**, **3f**, **3k** and **3q**. For most compounds, the apparent inhibition values were negative, which is interpreted here as absence of detectable inhibition rather than as evidence of enzyme activation.

These experimental data refine the computational picture. Whereas the multiple-docking/FCCorNet model prioritized the whole series as potential COX-1 and COX-2 ligands, direct enzymatic screening identified a single strong preliminary COX-1 hit at 1 µM, compound **3f**, together with two weaker COX-1-active compounds, and did not reproduce the predicted COX-2 activity at this screening concentration. This discrepancy indicates that the computational descriptors are best regarded as a prioritization layer rather than a quantitative predictor of inhibition. Compound **3f** therefore represents the principal experimentally supported, COX-1-skewed hit of this series and a starting point for subsequent dose–response (IC_50_), selectivity, and cell-based anti-inflammatory studies.

## 3. Materials and Methods

### 3.1. General Information

^1^H NMR (400 MHz) and ^13^C NMR (100 MHz) spectra were recorded on a Bruker Avance II 400 spectrometer (Billerica, MA, USA); ^1^H NMR (600 MHz) and ^13^C NMR (150 MHz) spectra were recorded on a Bruker Avance III 600 spectrometer (Billerica, MA, USA) in CDCl_3_, and chemical shifts are reported on the δ scale relative to Me_4_Si. ^1^H and ^13^C signal assignments were made using a combination of two-dimensional heteronuclear ^1^H–^13^C HSQC and ^1^H–^13^C HMBC experiments. Mass spectra were acquired on a Shimadzu GCMS-QP2010 Ultra instrument (Kyoto, Japan) with electron-impact (EI) ionization. Melting points were determined on a Boetius apparatus (VEB Dresden Analytik, Dresden, Germany). Column chromatography was performed on Merck (Darmstadt, Germany) silica gel 60 (0.063–0.200 mm). Reaction progress was monitored by TLC on SiO_2_ plates (0.25 mm; Merck 60 F_254_).

Imidazo[1,2-*a*]pyridines **1a**–**f** and [1,2,5]oxadiazolo[3,4-*b*]pyrazines **2a**–**c** were synthesized according to previously published procedures [24,25]. The photocatalyst TiO_2_ (rutile, particle size 10–20 nm) was prepared as reported in the literature [26]. Solvents were of chemically pure grade from domestic suppliers. Copies of the ^1^H and ^13^C NMR spectra of compounds **3a**–**r** are provided in the Supplementary Materials.

### 3.2. General Procedure for the Synthesis of Compounds ***3a***–***r***

A quartz flask containing a solution of the imidazo[1,2-a]pyridine **1a**–**f** (0.5 mmol) and the [1,2,5]oxadiazolo[3,4-b]pyrazine **2a**–**c** (0.5 mmol) in acetic acid (20 mL), together with the TiO_2_ photocatalyst (10 wt %), was sonicated for 3 min to give a suspension. The reaction mixture was irradiated with a Xe lamp (5000 K, 35 W, 4000 lm) positioned approximately 20 cm from the quartz flask and refluxed for 6 h. After completion of the reaction, the solution was concentrated under reduced pressure, and the residue was purified by preparative column chromatography on SiO_2_.

The detailed characterization data, elemental analyses, mass-spectrometric data, and copies of the ^1^H and ^13^C NMR spectra of compounds **3a**–**r** are provided in the Supplementary Materials.

### 3.3. Multiple Docking

Optimized 3D models of compounds **3a**–**r** were constructed using MarvinSketch 17.1.23 [27]. Ten lowest-energy conformers were generated separately for each compound. The conformers were optimized with MOPAC2012 [28] using the semiempirical quantum-chemical PM7 method. For each compound, the single conformer with the lowest total energy was selected from the optimized set.

**Figure 3:**
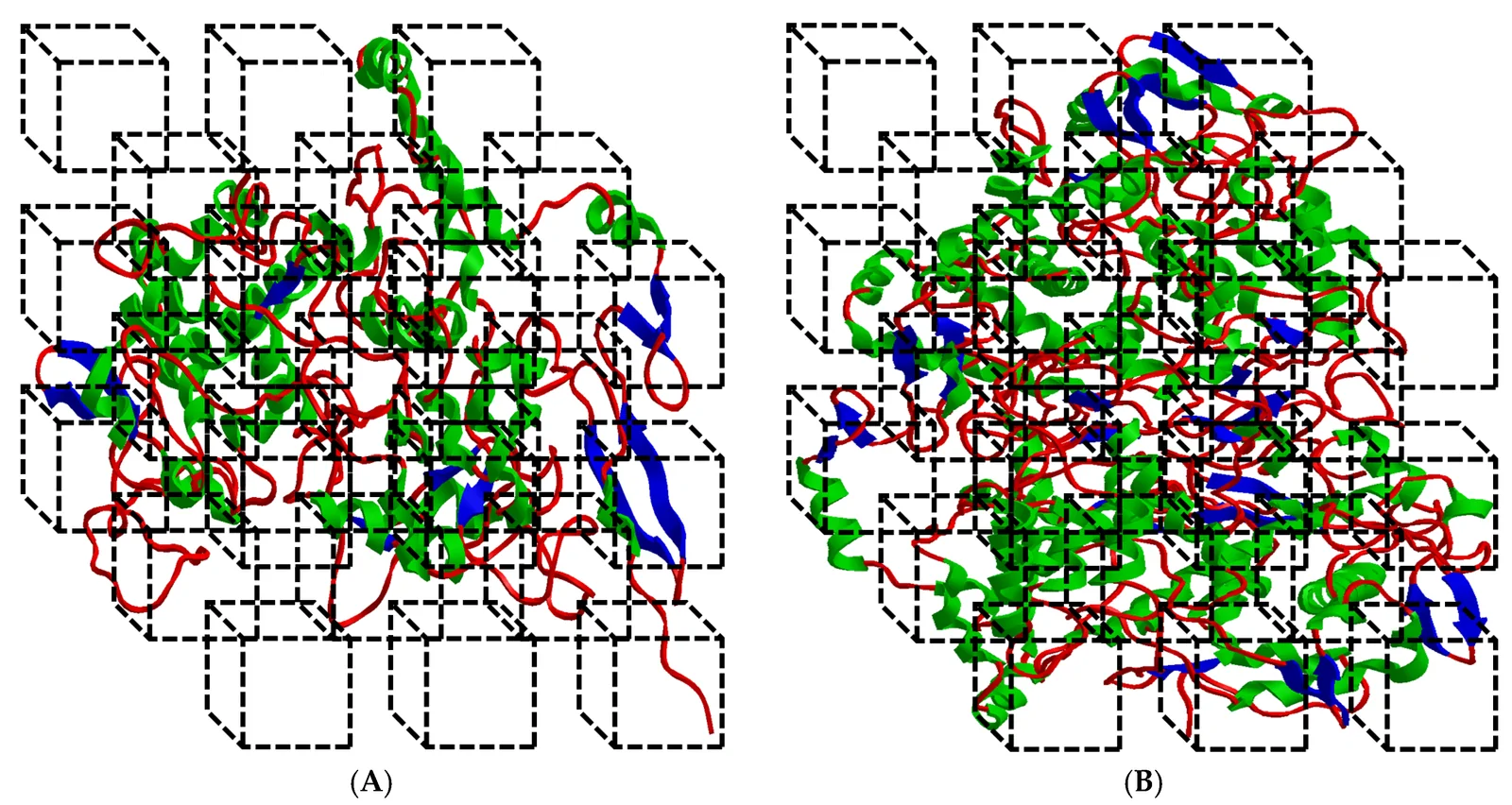
Construction of the multiple-docking spaces on the 3D models of human cyclooxygenase-1 (COX-1; PDB 6Y3C, (**A**)) and human cyclooxygenase-2 (COX-2; PDB 5KIR, (**B**)).

The calculations used the experimental 3D models of human cyclooxygenase-1 (COX-1; PDB 6Y3C) [29] and human cyclooxygenase-2 (COX-2; PDB 5KIR) [30]. On each model, 27 multiple-docking spaces covering the entire volume of each target protein were constructed with the in-house program MSite 5.6.25, following the multiple-docking energy-spectrum algorithm described previously [31], using a 3 × 3 × 3 grid with a constant step (Figure 3). Identification of a specific binding site is not required for multiple docking.

Multiple ensemble docking of the optimized 3D models of the new compounds was carried out with AutoDock Vina 1.1.1 [32] into the 27 spaces formed on each COX-1 and COX-2 model. For each of the 27 spaces and each compound, docking was performed five times, each in a separate run, considering the 10 most energetically favorable ligand conformations. From the resulting 50 docking-energy values, the effective multiple-docking energy ΔEli was determined for each compound l in each space i of the target:

(1) $$\Delta E_{li}=\overset{5}{\underset{j=1}{\min}}\left(\overset{10}{\underset{k=1}{\min}}\left(\Delta E_{lijk}\right)\right)$$

where *l* is the compound index, *l* = 1…*N*; *i* is the docking-space index, *i* = 1…27; *j* is the docking-run index, *j* = 1…5; *k* is the conformation index in docking run *j*, *k* = 1…10; and *N* is the number of compounds.

### 3.4. Construction of the Fully Connected Convolutional Correlation Neural Network Based on Multiple-Docking Energy Spectra

For multiple docking into a single target, the variables Δ*E_li_* calculated by Equation (1) can be regarded as neurons of a fully connected neural network with a symmetric connection matrix. They are interdependent because they are defined for the same protein. In a fully connected network, the interneuron connections (synapses) are linear, so their weights can be computed as pairwise correlation coefficients between the signals of two connected neurons. Thus, taking into account the boundary conditions of multiple docking, the energy of the fully connected convolutional correlation neural network for compound l can be expressed as:

(2) $$W_{l}=\frac{1}{2}\cdot \frac{1}{27}\sum_{\begin{array}{c} i,j=1\\ i\neq j \end{array}}^{27}R_{ij}\cdot {∆E}_{li}\cdot {∆E}_{lj},\;l=1\ldots N$$

where *R*_ij_ is the Pearson correlation coefficient between the energies Δ*E*_il_ and Δ*E*_jl_, *i* ≠ *j*; Δ*E*_li_ is energy *i* for compound *l*, *l* = 1…*N*; Δ*E*_lj_ is energy *j* for compound *l*, *l* = 1…*N*; and *N* is the number of compounds.

Equation (2) defines the architecture of the new fully connected convolutional neural network based on the correlation convolution of multiple-docking energy spectra, developed specifically for the in silico discovery of biologically active compounds [33].

**Figure 4:**
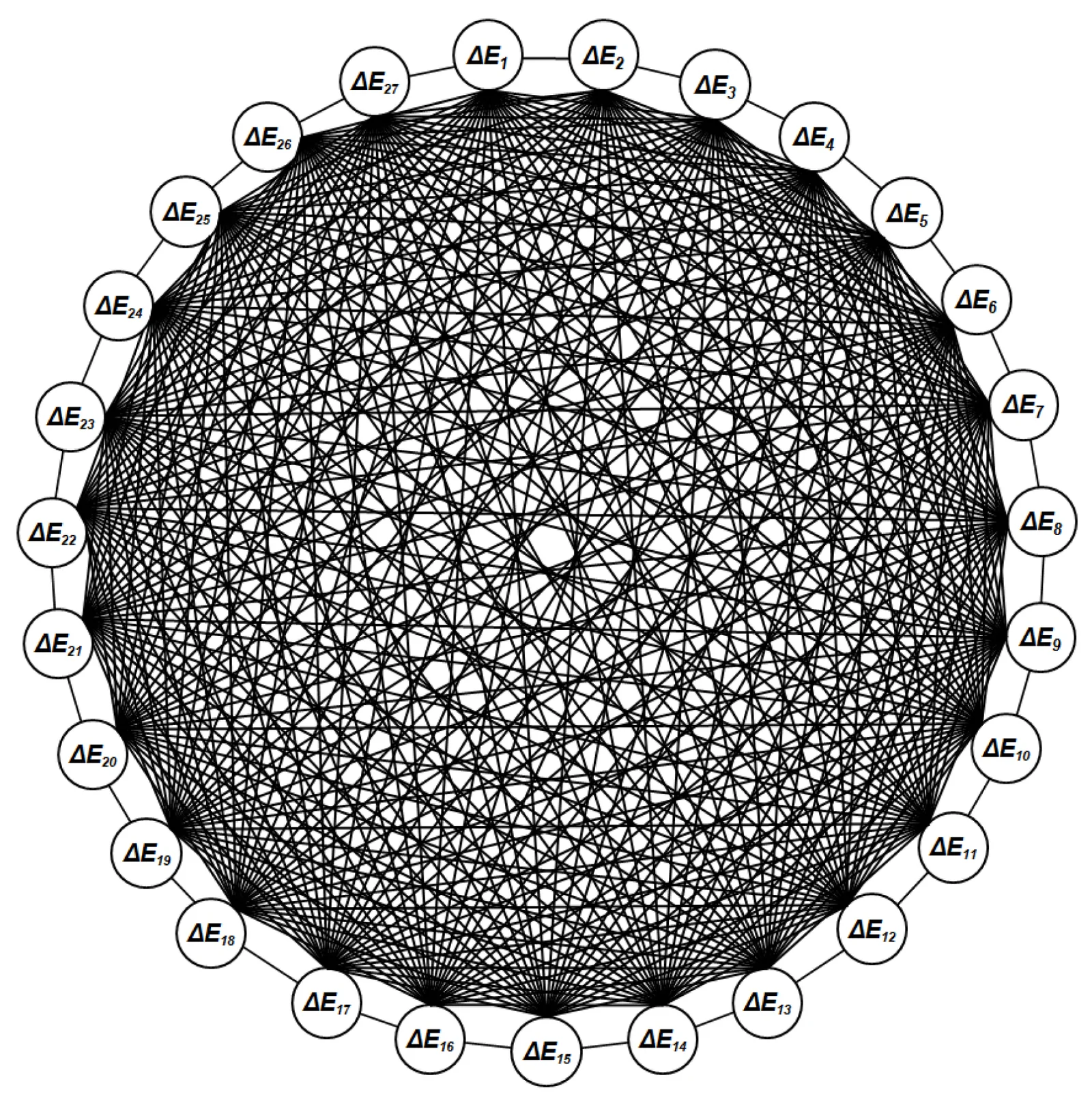
Architecture of the fully connected convolutional correlation neural network based on the multiple-docking energy spectrum.

The architecture of this new neural network is shown in Figure 4.

The fully connected convolutional correlation neural networks for COX-1 and COX-2 based on multiple-docking energy spectra were built with the in-house program FCCorNet 11.3.25 [33].

### 3.5. Affinity Descriptors

The affinity of the new compounds to COX-1 and COX-2 was characterized by the neural-network energies calculated for each compound by Equation (2). This parameter reflects the integral affinity of the predicted compound to the entire volume of the target protein. In addition, as a local-affinity descriptor, the minimum multiple-docking energy was calculated for each compound l:

(3) $$\Delta E_{min,l}=\overset{27}{\underset{i=1}{\min}}\left(\Delta E_{li}\right)$$

where *l* is the compound index, *l* = 1…*N*; *i* is the docking-space index, *i* = 1…27; Δ*E*_li_ is the docking energy into space *i*; and *N* is the number of compounds.

All predicted new compounds were sorted according to their integral affinity, expressed as the fully connected convolutional correlation neural-network energy *W*_l_ calculated by Equation (2), separately for COX-1 and COX-2.

### 3.6. COX-1 and COX-2 Fluorometric Inhibitor Assays

Inhibition of COX-1 and COX-2 was measured with commercial fluorometric COX inhibitor-screening assay kits (COX-1, ab204698; COX-2, ab283401; Abcam, Cambridge, UK) according to the manufacturer’s instructions. In these kits, COX-1 is ovine COX-1 and COX-2 is human recombinant COX-2. Each test compound was assayed at a final concentration of 1 µM. SC-560 and celecoxib were used as selective reference inhibitors of COX-1 and COX-2, respectively. Fluorescence was recorded on a CLARIOstar microplate reader (BMG Labtech, Ortenberg, Germany) at excitation/emission wavelengths of 535/587 nm. Each measurement was performed in triplicate, and the percentage inhibition relative to the uninhibited enzyme control is reported as mean ± SD.

### 3.7. Single-Crystal X-Ray Diffraction

The X-ray diffraction data set for 3l was collected on a Bruker D8 Venture diffractometer (Billerica, MA, USA) equipped with a CCD camera and a graphite-monochromated MoKα radiation source (λ = 0.71073 Å). A semiempirical absorption correction was applied [34]. The structure was solved and refined with SHELXL [35] using Olex2 [36]. The most important experimental crystallographic data and refinement statistics for 3l are as follows: C21H11FN6OS, M = 414.42 g/mol, T = 293(2) K, monoclinic P21/c, a = 20.179(8) Å, b = 7.815(5) Å, c = 12.170(9) Å, β = 73.369(15)°, V = 1838.9(19) Å3, Z = 4, Tmin/Tmax = 0.6478/0.7464, μ = 0.214 mm-1, dcalc = 1.497 g/cm3, θmax = 32.02°, Rint = 0.0281, 16877 measured reflections, 5496 independent reflections, 4424 reflections with I > 2σ(I), GooF = 1.093, R1(I > 2σ(I)) = 0.0564, wR2(I > 2σ(I)) = 0.1522, R1(all) = 0.0692, wR2(all) = 0.1614, ρmin/ρmax = −0.779/0.459 e∙Å-3. Supplementary crystallographic data for the compounds synthesized are given in CCDC number 2570305. These data can be obtained free of charge from The Cambridge Crystallographic Data Centre via www.ccdc.cam.ac.uk/data_request/cif (accessed on 10 August 2026).

## 4. Conclusions

A metal-free, TiO_2_-mediated C–H/C–H coupling of imidazo[1,2-*a*]pyridines with [1,2,5]oxadiazolo[3,4-*b*]pyrazines was developed using a simple oxidative photocatalytic system based on atmospheric oxygen, nanosized TiO_2_, and light irradiation. The method provides rapid access to 18 C3-heteroarylated biheteroaryl scaffolds of potential interest for pharmaceutical and materials applications.

The synthesized compounds were prioritized in silico with a fully connected convolutional correlation neural network based on multiple-docking energy spectra, and this prioritization was then tested directly by fluorometric COX-1 and COX-2 inhibitor screening at 1 µM. The experimental screen identified compound **3f** as a strong preliminary COX-1 hit at this concentration (86.64 ± 5.18%), with **3h** and **3l** showing weaker COX-1 inhibition, whereas no compound reached high or moderate COX-2 inhibition under the screening conditions.

The agreement between computational prioritization and enzymatic screening was therefore partial, which underlines that the in silico descriptors are best used as a ranking layer rather than as a quantitative predictor of activity. Compound **3f** represents the principal experimentally supported, COX-1-skewed hit of this series and a rational starting point for subsequent dose–response (IC_50_), selectivity, and cell-based anti-inflammatory studies; however, potency and selectivity claims will require IC_50_ determination and broader biological validation.

## Figures and Tables

**Table 1:** Optimization of the C–H/C–H coupling of imidazo[1,2-*a*]pyridine **1a** with [1,2,5]oxadiazolo[3,4-*b*]pyrazine **2a** ^[a]^.

No.	Catalyst/Acid	Solvent	TiO_2_ (wt %)	Temperature (°C)	Yield (%)
1	AcOH	–	–	120	8
2 ^[b]^	AcOH	–	10	rt	0
3 ^[b]^	AcOH	–	10	120	62
4	BF_3_·OEt_2_	MeCN	–	rt	0
5 ^[b]^	CF_3_COOH	MeCN	10	rt	0
6 ^[b]^	CF_3_COOH	1,4-dioxane	10	120	trace
7 ^[b]^	CF_3_SO_3_H	MeCN	10	rt	0
8 ^[b]^	CF_3_SO_3_H	1,4-dioxane	10	120	trace
9	I_2_	MeOH	–	60	0
10 ^[b]^	AcOH	–	10 (anatase)	120	36

^[a]^ Reaction conditions: imidazo[1,2-*a*]pyridine **1a** (0.5 mmol), [1,2,5]oxadiazolo[3,4-*b*]pyrazine **2a** (0.5 mmol), the corresponding acid, solvent and temperature. ^[b]^ The reaction was run under irradiation with a Xe lamp (5000 K, 35 W, 4000 lm) positioned approximately 20 cm from the quartz flask and refluxed for 6 h. Unless stated otherwise, the TiO_2_ used was the rutile nanopowder (10–20 nm) prepared as described in Section 3.1; entry 10 was run with commercial anatase TiO_2_ for comparison.

**Table 2:** Yields of compounds **3a**–**r** ^a^.

№	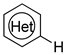	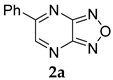	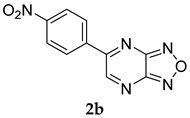	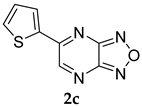
1	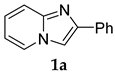	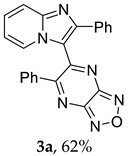	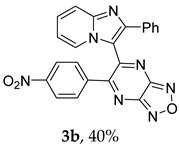	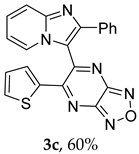
2	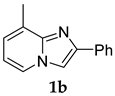	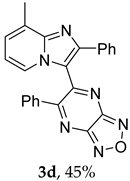	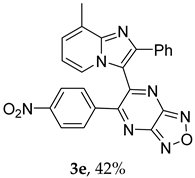	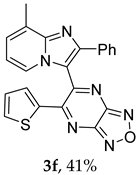
3	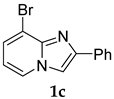	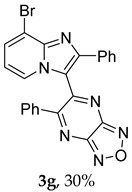	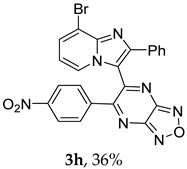	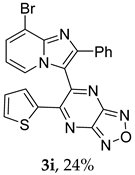
4	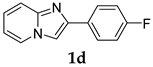	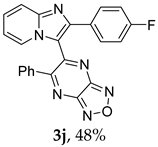	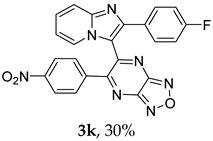	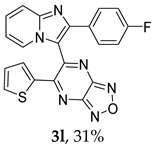
5	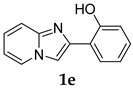	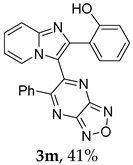	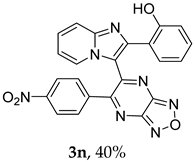	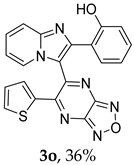
6	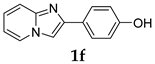	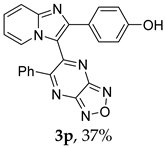	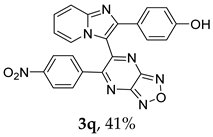	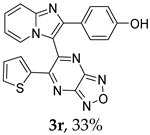

^a^ Reaction conditions: imidazo[1,2-*a*]pyridine **1a**–**f**, [1,2,5]oxadiazolo[3,4-*b*]pyrazine **2a**–**c**, TiO_2_ (10 wt %; rutile, 10–20 nm, prepared as described in Section 3.1), AcOH, reflux for 6 h under irradiation with a Xe lamp (5000 K, 35 W, 4000 lm). Yields of isolated products.

**Table 3:** Calculated affinity of compounds **3a**–**r** toward cyclooxygenase-1 (COX-1) ^[a,b]^.

№	*W* ^[a]^	Δ*E* ^[b]^	№	*W* ^[a]^	Δ*E* ^[b]^	№	*W* ^[a]^	Δ*E* ^[b]^
**3k**	359	−8.8	**3b**	345	−8.6	**3g**	332	−8.7
**3e**	357	−8.8	**3h**	345	−8.7	**3c**	330	−8.5
**3n**	356	−8.7	**3m**	344	−8.7	**3f**	329	−8.4
**3q**	356	−8.8	**3a**	343	−8.8	**3r**	327	−8.5
**3j**	353	−9.0	**3l**	340	−8.7	**3o**	326	−8.4
**3d**	346	−8.8	**3p**	340	−8.8	**3i**	322	−8.4

^[a]^ *W* is the energy of the fully connected convolutional correlation neural network based on multiple-docking energies (arb. units); compounds are listed in order of decreasing *W*. ^[b]^ Δ*E* is the minimum energy in the multiple-docking energy spectrum (kcal/mol).

**Table 4:** Calculated affinity of compounds **3a**–**r** toward cyclooxygenase-2 (COX-2) ^[a,b]^.

№	*W* ^[a]^	Δ*E* ^[b]^	№	*W* ^[a]^	Δ*E* ^[b]^	№	*W* ^[a]^	Δ*E* ^[b]^
**3k**	300	−10.2	**3h**	282	−10.3	**3m**	273	−9.5
**3q**	299	−10.1	**3l**	280	−9.8	**3c**	267	−9.4
**3b**	295	−10.1	**3p**	279	−9.7	**3f**	264	−9.4
**3n**	292	−10.2	**3a**	278	−9.8	**3o**	264	−9.8
**3e**	288	−10.4	**3d**	277	−10.3	**3r**	264	−9.6
**3j**	288	−9.9	**3g**	274	−10.1	**3i**	254	−9.2

^[a]^ *W* is the energy of the fully connected convolutional correlation neural network based on multiple-docking energies (arb. units); compounds are listed in order of decreasing *W*. ^[b]^ Δ*E* is the minimum energy in the multiple-docking energy spectrum (kcal/mol).

**Table 5:** In vitro COX-1 and COX-2 inhibition by compounds **3a**–**r** at 1 µM ^a^.

Compound	COX-1 Inhibition at 1 µM (%) ^b^	COX-2 Inhibition at 1 µM (%) ^b^
**3a**	−66.71 ± 12.02	−31.35 ± 14.96
**3b**	−38.81 ± 9.78	−12.31 ± 12.62
**3c**	−26.70 ± 12.58	−27.51 ± 11.21
**3d**	−72.48 ± 16.99	−15.72 ± 10.85
**3e**	−52.65 ± 14.16	12.71 ± 23.94
**3f**	86.64 ± 5.18	12.64 ± 7.72
**3g**	−16.50 ± 25.25	−8.11 ± 4.11
**3h**	31.05 ± 8.34	8.49 ± 2.08
**3i**	−37.98 ± 35.81	3.01 ± 4.21
**3j**	−51.94 ± 12.44	−20.32 ± 12.93
**3k**	−48.93 ± 30.07	12.14 ± 3.87
**3l**	15.91 ± 11.20	6.43 ± 4.04
**3m**	−38.55 ± 14.43	−22.61 ± 16.91
**3n**	−44.83 ± 30.08	0.15 ± 3.54
**3o**	−62.93 ± 25.70	6.58 ± 3.55
**3p**	−3.78 ± 24.47	1.01 ± 3.03
**3q**	−5.90 ± 14.39	10.88 ± 2.78
**3r**	−8.62 ± 11.29	16.97 ± 2.53

^a^ Values are mean ± SD of triplicate measurements at a single screening concentration of 1 µM. ^b^ Reference inhibitors on the two assay plates: SC-560 (COX-1) 83.36 ± 7.25% and 90.24 ± 3.68%; celecoxib (COX-2) 95.44 ± 1.66% and 91.76 ± 7.10%. Negative apparent inhibition values mean that the fluorescence slope measured in the presence of the test compound exceeded that of the uninhibited enzyme control. In this screening format, these values should not be interpreted as enzymatic activation; they more conservatively indicate absence of detectable inhibition under the assay conditions and may reflect assay variability, compound-dependent fluorescence effects, or kinetic noise.

## Data Availability

The data presented in this study are available within the article and its Supplementary Materials. Crystallographic data for **3l** have been deposited with the Cambridge Crystallographic Data Centre (CCDC 2570305) and can be obtained free of charge via www.ccdc.cam.ac.uk/data_request/cif (accessed on 10 August 2026).

## Supplementary Materials

The following supporting information can be downloaded at: https://www.mdpi.com/article/10.3390/molecules31162830/s1.
