# Supplementary material for: Light-Promoted C–H/C–H Coupling of Imidazo[1,2-a]pyridines with 5-(Hetero)aryl-1,2,5-oxadiazolo[3,4-b]pyrazines over TiO2 and Experimental/In Silico Evaluation of COX-1 and COX-2 Inhibitory Activity
Source: Molecules. 2026 Aug 13;31(16):2830. doi: 10.3390/molecules31162830 (PMC13515192; doi:10.3390/molecules31162830)
Supplement: Supplementary file 1 [file molecules-31-02830-s001.zip › molecules-4470251-supplementary.pdf]

Supporting Information

Light-Promoted C–H/C–H Coupling of Imidazo[1,2-*a*]pyridines with 5-(Hetero)aryl-1,2,5-oxadiazolo[3,4-*b*]pyrazines over TiO_2 and Experimental/In Silico Evaluation of COX-1 and COX-2 Inhibitory Activity

Maria A. Trestsova [1,2,3], Daria A. Andreeva [1], Mikhail A. Kiskin [4], Maria V. Komelkova [3], Pavel M. Vassiliev [3,5], Alena. S. Taran [3], Ludmila A. Yolshina [6], Alexander G. Kvashnichev [6], Veronika A. Isaeva [7], Irina A. Utepova [1,2,3,*], Oleg N. Chupakhin [1,2] and Alexey P. Sarapultsev [3,*]

[1] Department of Organic and Biomolecular Chemistry, Ural Federal University, 28 Mira Street, 620002 Ekaterinburg, Russia; mtrescova@mail.ru (M.A.T.); andreevadarya03@mail.ru (D.A.A.); chupakhin@ios.uran.ru (O.N.C.)
[2] Institute of Organic Synthesis of the Russian Academy of Sciences, 22 S. Kovalevskoy Street, 620066 Ekaterinburg, Russia
[3] Russian-Chinese Education and Research Center of System Pathology, South Ural State University, 76 Lenin prosp., 454080 Chelyabinsk, Russia; mkomelkova@mail.ru (M.V.K.); pvassiliev@mail.ru (P.M.V.)
[4] Kurnakov Institute of General and Inorganic Chemistry of the Russian Academy of Sciences, 31 Leninsky prosp., 119991 Moscow, Russia; mkiskin@igic.ras.ru
[5] Scientific Center for Innovative Medicines, Volgograd State Medical University, 1 Pavshikh Bortsov Square, 400131 Volgograd, Russia
[6] Institute of High-Temperature Electrochemistry, Ural Branch of the Russian Academy of Sciences, 20 Akademicheskaya Street, 620066 Ekaterinburg, Russia; kvashnichev@bk.ru (A.G.K.)
[7] Institute of Immunology and Physiology, Ural Branch of the Russian Academy of Sciences, 106 Pervomaiskaya Street, 620049 Ekaterinburg, Russia
* Correspondence: i.a.utepova@urfu.ru (I.A.U.); a.sarapultsev@gmail.com (A.P.S.)

Table of Contents

1. Characterization data of **3a-r** 2

2. ^{1}H NMR spectra of **1a-f** and **2a-c** 8

3. ^{1}H NMR and ^{13}C NMR spectra of **3a-r** 13

4. Single-crystal X-ray diffraction analysis the structure of **3l** 31

1. Characterization data of **3a-r**

5-Phenyl-6-(2-phenylimidazo[1,2-*a*]pyridin-3-yl)[1,2,5]oxadiazolo[3,4-*b*]pyrazine 3a. The residue was purified by column chromatography on silica gel eluting with mixture hexane/ethyl acetate 9:1 to give compound as a dark-red solid (121 mg, 62 %), mp 261-263 °C. R_f = 0.1. ^{1}H NMR (400 MHz, $CDCl_3$) δ 9.17 (d, J = 6.9 Hz, 1H, 5′-H), 7.74 (d, J = 8.9 Hz, 1H, 8′-H), 7.46 (t, J = 7.8 Hz, 1H, 7′-H), 7.24 – 7.16 (m, 1H, Ph′), 7.13 (t, J = 7.4 Hz, 1H, Ph), 7.06 (q, J = 7.4, 6.7 Hz, 3H, Ph, 6′-H), 6.90 (t, J = 7.6 Hz, 2H, Ph′), 6.81 (dd, J = 16.4, 7.6 Hz, 4H, Ph, Ph′). ^{13}C NMR (101 MHz, $CDCl_3$) δ 164.14, 153.74, 152.08, 150.92, 150.88, 147.48, 136.79, 133.65, 130.32, 128.85, 128.72, 128.60, 128.54, 128.14, 127.89, 125.91, 118.05, 117.99, 114.51. MS (EI): *m/z* 390 ($[M]^+$, 100%). Anal. calcd. for $C_{23}H_{14}N_6O$: C, 70.76; H, 3.61; N, 21.53. Found: C, 70.62; H, 3.62; N, 21.46.

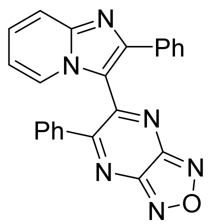

5-(4-Nitrophenyl)-6-(2-phenylimidazo[1,2-*a*]pyridin-3-yl)[1,2,5]oxadiazolo[3,4-*b*]pyrazine 3b. The residue was purified by column chromatography on silica gel eluting with mixture hexane/ethyl acetate 8:2 to give compound as a dark-red solid (87 mg, 40 %), mp >300 °C. R_f = 0.1. ^{1}H NMR (400 MHz, $CDCl_3$) δ 9.37 (d, J = 7.0 Hz, 1H, 5′-H), 7.90 – 7.81 (m, 3H, 8′-H, Ph′), 7.66 – 7.58 (m, 1H, 7′-H), 7.36 (t, J = 7.4 Hz, 1H, 6′-H), 7.26 – 7.14 (m, 3H, Ph′), 7.06 (d, J = 8.8 Hz, 2H, Ph), 6.92 (d, J = 7.1 Hz, 2H, Ph). ^{13}C NMR (150 MHz, $CDCl_3$) δ 162.00, 152.62, 150.91, 150.58, 148.14, 148.11, 142.51, 133.76, 129.40, 129.29, 129.06, 128.81, 128.63, 128.38, 126.13, 124.63, 123.02, 122.80, 118.26, 117.39, 114.94. MS (EI): *m/z* 435 ($[M]^+$, 100%). Anal. calcd. for $C_{23}H_{13}N_7O_3$: C, 63.45; H, 3.01; N, 22.52. Found: C, 63.41; H, 3.02; N, 22.60.

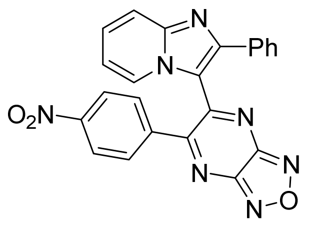

5-(Thien-2-yl)-6-(2-phenylimidazo[1,2-*a*]pyridin-3-yl)[1,2,5]oxadiazolo[3,4-*b*]pyrazine 3c. The residue was purified by column chromatography on silica gel eluting with mixture benzene/ethyl acetate 8:2 to give compound as a dark-red solid (119 mg, 60 %), mp 258-260 °C. R_f = 0.2. ^{1}H NMR (400 MHz, $CDCl_3$) δ 8.91 (d, J = 6.8 Hz, 1H, 5′-H), 7.79 (d, J = 8.9 Hz, 1H, 8′-H), 7.45 (t, J = 7.7 Hz, 1H, 7′-H), 7.26 (d, J = 3.2 Hz, 1H, thiophene), 7.20 – 7.18 (m, 1H, Ph), 7.15 – 7.12 (m, 4H, Ph), 7.03 (t, J = 6.7 Hz, 1H, 6′-H), 6.85 (d, J = 3.2 Hz, 1H, thiophene), 6.66 (t, J = 4.2 Hz, 1H, thiophene). ^{13}C NMR (101 MHz, $CDCl_3$) δ 156.07, 151.91, 150.75, 149.85, 149.69, 146.32, 139.47, 132.61, 132.40, 130.63, 127.99, 127.78, 127.52, 127.48, 126.68, 124.68,

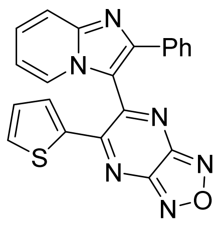

116.97, 116.61, 113.50. MS (EI): *m/z* 396 ([M]$^+$, 100%). Anal. calcd. for $C_{21}H_{12}N_6OS$: C, 63.63; H, 3.05; N, 21.20. Found: C, 63.59; H, 3.04; N, 21.27.

5-Phenyl-6-(2-phenyl-8-methylimidazo[1,2-*a*]pyridin-3-yl)[1,2,5]oxadiazolo[3,4-*b*]pyrazine 3d. The

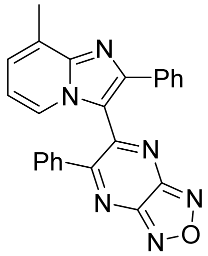

residue was purified by column chromatography on silica gel eluting with mixture hexane/ethyl acetate 8:2 to give compound as a dark-red solid (91 mg, 45 %), mp 188-190 °C. R_f = 0.1. ^{1}H NMR (600 MHz, $CDCl_3$) δ 9.02 (d, J = 6.9 Hz, 1H, 5′-H), 7.22 (d, J = 6.9 Hz, 1H, 7′-H), 7.15 (t, J = 7.4 Hz, 1H, Ph′), 7.12 (t, J = 7.4 Hz, 1H, Ph), 7.02 (t, J = 7.7 Hz, 2H, Ph), 6.94 (t, J = 6.9 Hz, 1H, 6′-H), 6.89 (t, J = 7.8 Hz, 2H, Ph′), 6.81 (dd, J = 13.8, 7.6 Hz, 4H, Ph, Ph′), 2.63 (s, 3H, CH_3′). ^{13}C NMR (150 MHz, $CDCl_3$) δ 163.19, 152.84, 150.75, 149.89, 149.85, 146.88, 135.89, 132.96, 129.18, 127.65, 127.61, 127.58, 127.08, 127.07, 126.83, 126.43, 122.66, 117.50, 113.39, 16.14. MS (EI): *m/z* 404 ([M]$^+$, 100%). Anal. calcd. for $C_{24}H_{16}N_6O$: C, 71.28; H, 3.99; N, 20.78. Found: C, 71.19; H, 4.01; N, 20.71

5-(4-Nitrophenyl)-6-(2-phenyl-8-methylimidazo[1,2-*a*]pyridin-3-yl)[1,2,5]oxadiazolo[3,4-*b*]pyrazine 3e.

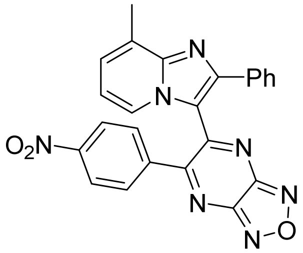

The residue was purified by column chromatography on silica gel eluting with mixture hexane/ethyl acetate 8:2 to give compound as a dark-red solid (94 mg, 42 %), mp 258-260 °C. R_f = 0.1. ^{1}H NMR (600 MHz, $CDCl_3$) δ 9.23 (d, J = 6.9 Hz, 1H, 5′-H), 7.85 (d, J = 8.7 Hz, 2H, Ph), 7.40 (d, J = 7.0 Hz, 1H, 7′-H), 7.34 (t, J = 7.4 Hz, 1H, Ph′), 7.17 (t, J = 7.7 Hz, 2H, Ph′), 7.12 (t, J = 6.9 Hz, 1H, 6′-H), 7.06 (d, J = 8.7 Hz, 2H, Ph), 6.95 – 6.88 (m, 2H, Ph′), 2.75 (s, 3H, CH_3′). ^{13}C NMR (150 MHz, $CDCl_3$) δ 162.08, 152.77, 152.19, 150.97, 150.57, 148.48, 148.10, 142.67, 134.03, 129.21, 129.04, 128.76, 128.48, 128.19, 123.87, 122.80, 117.85, 114.92, 17.14. MS (EI): *m/z* 449 ([M]$^+$, 100%). Anal. calcd. for $C_{24}H_{15}N_7O_3$: C, 64.14; H, 3.36; N, 21.82. Found: C, 64.18; H, 3.35; N, 21.93

5-(Thien-2-yl)-6-(2-phenyl-8-methylimidazo[1,2-*a*]pyridin-3-yl)[1,2,5]oxadiazolo[3,4-*b*]pyrazine 3f. The

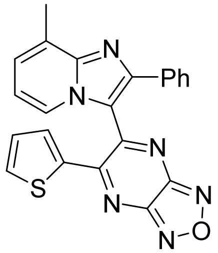

residue was purified by column chromatography on silica gel eluting with mixture hexane/ethyl acetate 8:2 to give compound as a dark-red solid (84 mg, 41 %), mp 168-170 °C. R_f = 0.1. ^{1}H NMR (600 MHz, $CDCl_3$) δ 8.87 (d, J = 6.9 Hz, 1H, 5′-H), 7.36 – 7.34 (m, 1H, thiophene), 7.32 (d, J = 6.9 Hz, 1H, 7′-H), 7.29 – 7.25 (m, 1H, Ph′), 7.23 – 7.17 (m, 4H, Ph′), 7.02 (t, J = 6.9 Hz, 1H, 6′-H), 6.97 – 6.94 (m, 1H, thiophene), 6.78 – 6.74 (m, 1H, thiophene), 2.77 (s, 3H, CH_3′). ^{13}C NMR (150 MHz, $CDCl_3$) δ 157.21, 153.13, 151.57, 150.85, 150.78, 147.83, 140.70, 133.83, 133.55, 131.62, 128.77, 128.62, 128.34, 128.18, 127.69, 127.36, 123.50, 118.15, 114.45, 17.21. MS (EI): *m/z* 410 ([M]$^+$, 100%). Anal. calcd. for $C_{22}H_{14}N_6OS$: C, 64.38; H, 3.44; N, 20.48. Found: C, 64.60; H, 3.43; N, 20.41.

5-Phenyl-6-(2-phenyl-8-bromoimidazo[1,2-*a*]pyridin-3-yl)[1,2,5]oxadiazolo[3,4-*b*]pyrazine 3g. The

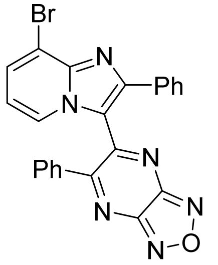

residue was purified by column chromatography on silica gel eluting with mixture hexane/ethyl acetate 9:1 to give compound as a dark-red solid (70 mg, 30 %), mp 238-240 °C. R_f = 0.1. ^{1}H NMR (600 MHz, $CDCl_3$) δ 9.19 (d, J = 6.8 Hz, 1H, 5′-H), 7.77 (d, J = 7.4 Hz, 1H, 7′-H), 7.34 – 7.18 (m, 2H, Ph, Ph′), 7.12 (t, J = 7.5 Hz, 2H, Ph), 7.05 – 6.97 (m, 3H, 6′-H, Ph′), 6.93 (d, J = 7.5 Hz, 2H, Ph′), 6.87 (d, J = 7.6 Hz, 2H, Ph). ^{13}C NMR (150 MHz, $CDCl_3$) δ 163.93, 153.68, 152.20, 150.89, 150.77, 145.45, 136.48, 133.33, 130.70, 130.42, 128.97, 128.75, 128.71, 128.10, 128.03, 125.08, 119.46, 114.33, 112.09. MS (EI): *m/z* 470 ($[M+H]^+$, 100%). Anal. calcd. for $C_{23}H_{13}BrN_6O$: C, 58.86; H, 2.79; N, 17.91. Found: C, 58.60; H, 2.80; N, 17.99.

5-(8-Bromo-2-phenylimidazo[1,2-a]pyridin-3-yl)-6-(4-nitrophenyl)[1,2,5]oxadiazolo[3,4-b]pyrazine 3h.

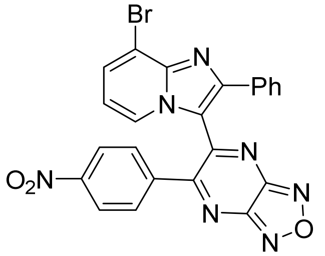

The residue was purified by column chromatography on silica gel eluting with mixture benzene/ethyl acetate 9:1 to give compound as a dark-red solid (93 mg, 36%), mp 280-282 °C. R_f = 0.1. ^{1}H NMR (400 MHz, DMSO-d_6) δ 9.15 (d, J = 6.8 Hz, 1H, 5′-H), 7.86 (d, J = 7.4 Hz, 1H, 7′-H), 7.77 (d, J = 8.5 Hz, 2H, Ph), 7.33 – 7.29 (m, 1H, 6′-H), 7.19 – 7.10 (m, 5H, Ph, Ph′), 7.04 (d, J = 6.9 Hz, 2H, Ph). ^{13}C NMR (101 MHz, DMSO-d_6) δ 163.07, 154.05, 152.24, 151.88, 150.04, 148.08, 145.07, 142.52, 133.39, 131.63, 130.31, 129.43, 129.14, 129.00, 128.79, 126.93, 122.77, 119.12, 114.71, 110.76. MS (EI): m/z 513 ($[M-H]^-$, 100%). Anal. calcd. for $C_{23}H_{12}BrN_7O_3$: C, 53.71; H, 2.35; N, 19.06. Found: C, 53.54; H, 2.36; N, 19.15.

5-(Thien-2-yl)-6-(2-phenyl-8-bromoimidazo[1,2-a]pyridin-3-yl)[1,2,5]oxadiazolo[3,4-b]pyrazine 3i. The

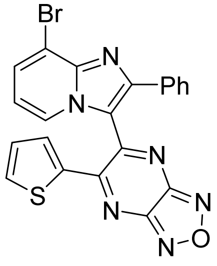

residue was purified by column chromatography on silica gel eluting with benzene to give compound as a dark-red solid (57 mg, 24 %), mp 228-230 °C. R_f = 0.1. ^{1}H NMR (600 MHz, $CDCl_3$) δ 8.93 (d, J = 6.7 Hz, 1H, 5′-H), 7.78 (d, J = 7.3 Hz, 1H, 7′-H), 7.37 (d, J = 4.7 Hz, 1H, thiophene), 7.29 – 7.27 (m, 1H, Ph′), 7.22 – 7.20 (m, 4H, Ph′), 6.98 (t, J = 7.3 Hz, 1H, 6′-H), 6.95 (m, J = 4.7 Hz, 1H, thiophene), 6.78 (t, J = 4.3 Hz, 1H, thiophene). ^{13}C NMR (150 MHz, $CDCl_3$) δ 156.80, 152.81, 151.91, 150.84, 150.61, 145.35, 140.19, 133.87, 133.13, 131.75, 130.59, 129.13, 128.78, 128.68, 127.90, 124.83, 119.04, 114.34, 112.14. MS (EI): m/z 476 ($[M+H]^+$, 100%). Anal. calcd. for $C_{21}H_{11}BrN_6OS$: C, 53.07; H, 2.33; N, 17.68. Found: 52.92; H, 2.32; N, 17.76.

5-Phenyl-6-(2-(4-fluorophenyl)imidazo[1,2-a]pyridin-3-yl)[1,2,5]oxadiazolo[3,4-b]pyrazine 3j. The

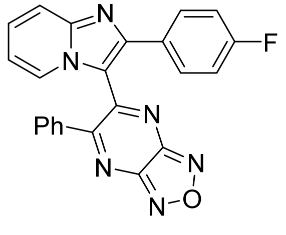

residue was purified by column chromatography on silica gel eluting with dichloromethane to give compound as a dark-red solid (98 mg, 48 %), mp 268-270 °C. R_f = 0.1. ^{1}H NMR (600 MHz, $CDCl_3$) δ 9.27 (d, J = 5.8 Hz, 1H, 5′-H), 7.82 (d, J = 8.3 Hz, 1H, 8′-H), 7.57 (m, 1H, 7′-H), 7.28 (m, J = 11.2 Hz, 1H, Ph), 7.17 (m, 1H, 6′-H), 7.06 (m, 2H, Ph), 7.01 – 6.80 (m, 6H, Ph, Ph′). ^{13}C NMR (150 MHz, $CDCl_3$) δ 163.87, 163.28 (d, J_{C-F} = 247.5 Hz), 153.47, 151.15, 150.85, 150.85, 147.59, 136.81, 130.57, 130.26 (d, J_{C-F} = 7.5 Hz), 130.09 (d, J_{C-F} = 3.0 Hz), 128.73, 128.16, 127.99, 125.95, 118.02, 115.79 (d, J_{C-F} = 21.0 Hz), 114.58. MS

(EI): m/z 408 ([M]$^+$, 100%). Anal. calcd. for $C_{23}H_{13}FN_6O$: C, 67.64; H, 3.21; N, 20.58. Found: C, 67.51; H, 3.22; N, 20.65.

5-(2-(4-Fluorophenyl)imidazo[1,2-*a*]pyridin-3-yl)-6-(4-nitrophenyl)[1,2,5]oxadiazolo[3,4-*b*]pyrazine 3k. The residue was purified by column chromatography on silica gel eluting with mixture benzene/ethyl acetate 9:1 to give compound as a dark-red solid (68 mg, 30%), mp 284-286 °C. R_f = 0.1. ^{1}H NMR (400 MHz, DMSO-d$_6$) δ 9.15 (d, J = 7.0 Hz, 1H, 8′-H), 7.89 (d, J = 8.7 Hz, 2H,Ph), 7.82 (d, J = 8.9 Hz, 1H, 5′-H), 7.63 (t, J = 7.8 Hz, 1H, 7′-H), 7.28 (t, J = 6.9 Hz, 1H, 6′-H), 7.21 (d, J = 8.8 Hz, 2H, Ph), 7.14 (dd,J = 8.6, 5.5 Hz, 2H, Ph′),6.97 (t, J = 8.8 Hz, 2H, Ph′). ^{13}C NMR (101 MHz, DMSO-d$_6$) δ 163.15, 163.08 (d, J_{C-F} = 246.0 Hz), 154.03, 152.31, 151.89, 149.30, 148.20, 147.36, 142.83, 131.22 (d, J_{C-F} = 8.0 Hz), 130.45 (d, J_{C-F} = 2.0 Hz), 130.34, 129.48, 128.79, 127.29, 122.76, 117.60, 117.53, 115.92 (d, J_{C-F} = 21.0 Hz), 114.55. MS (EI): m/z 453 ([M]$^+$, 100%). Anal. calcd. for $C_{23}H_{12}FN_7O_3$: C, 60.93; H, 2.67; N, 21.63. Found: C, 60.71; H, 2.68; N, 21.71.

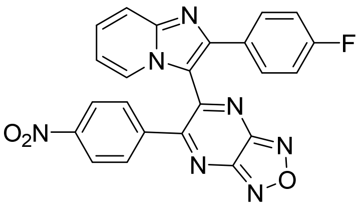

5-(2-(4-Fluorophenyl)imidazo[1,2-*a*]pyridin-3-yl)-6-(thien-2-yl)[1,2,5]oxadiazolo[3,4-*b*]pyrazine 3l. The residue was purified by column chromatography on silica gel eluting with mixture hexane/ethyl acetate 1:1 to give compound as a dark-red solid (64 mg, 31%), mp 244-246 °C. R_f = 0.2. ^{1}H NMR (600 MHz, DMSO-d$_6$) δ 8.83 (d, J = 6.9 Hz, 1H, 8′-H), 7.84 (d, J = 9.0 Hz, 1H, 5′-H), 7.74 (d, J = 4.9 Hz, 1H, thiophene), 7.56 (t, J = 6.8 Hz, 1H, thiophene), 7.54 – 7.48 (m, 2H, Ph′), 7.15 (t, J = 6.9 Hz, 1H, 7'-H), 7.09 – 7.01 (m, 2H, Ph′), 6.93 (d, J = 3.9 Hz, 1H, thiophene), 6.86 (t, J = 4.9 Hz, 1H, 6′-H). ^{13}C NMR (150 MHz, DMSO-d$_6$) δ 162.74 (d, J_{C-F} = 244.5 Hz), 157.26, 153.87, 152.10, 152.04, 147.72, 146.53, 139.94, 135.26, 132.67, 130.81 (d, J_{C-F} = 7.5 Hz), 130.23 (d, J_{C-F} = 3.0 Hz), 128.75, 128.68, 126.80, 117.56, 117.39, 115.90 (d, J_{C-F} = 21.0 Hz), 114.40. MS (EI): m/z 414 ([M]$^+$, 100%). Anal. calcd. for $C_{21}H_{11}FN_6OS$: C, 60.86; H, 2.68; N, 20.28. Found: C, 60.57; H, 2.68; N, 20.37.

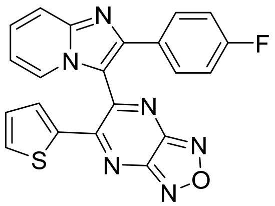

5-(2-(2-Hydroxyphenyl)imidazo[1,2-*a*]pyridin-3-yl)-6-phenyl[1,2,5]oxadiazolo[3,4-*b*]pyrazine 3m. The residue was purified by column chromatography on silica gel eluting with benzene to give compound as a dark-red solid (83 mg, 41%), mp 246-248 °C. R_f = 0.1. ^{1}H NMR (400 MHz, DMSO-d$_6$) δ 9.76 (s, 1H, OH), 8.91 (d, J = 6.9 Hz, 1H, 8′-H), 7.76 (d, J = 9.0 Hz, 1H, 5′-H), 7.54 – 7.46 (m, 1H, 7′-H), 7.24 (t, J = 7.4 Hz, 1H, 6′-H), 7.17 (d, J = 7.3 Hz, 2H, Ph′), 7.14 – 6.99 (m, 5H, Ph, Ph′), 6.63 (m, 2H, Ph). ^{13}C NMR (101 MHz, DMSO-d$_6$) δ 165.28, 156.92, 154.71, 151.80, 146.33, 146.05, 136.73, 130.77, 130.67, 130.20, 129.02, 128.14, 127.81, 126.65, 120.63, 119.65, 118.31, 117.16, 115.77, 113.91. MS (EI): m/z 406([M]$^+$, 100%). Anal. calcd. for $C_{23}H_{14}N_6O_2$: C, 67.97; H, 3.47; N, 20.68. Found: C, 67.69; H, 3.48; N, 20.75.

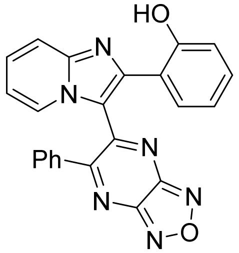

5-(2-(2-Hydroxyphenyl)imidazo[1,2-*a*]pyridin-3-yl)-6-(4-nitrophenyl)[1,2,5]oxadiazolo[3,4-*b*]pyrazine

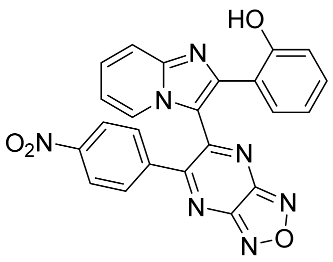

3n. The residue was purified by column chromatography on silica gel eluting with dichloromethane to give compound as a dark-red solid (90 mg, 40%), mp 236-238 °C. R_f = 0.2. ^{1}H NMR (400 MHz, DMSO-d_6) δ 9.69 (s, 1H, OH), 9.12 (d, J = 7.0 Hz, 1H, 8′-H), 7.85 (d, J = 8.9 Hz, 2H, Ph), 7.80 (d, J = 9.0 Hz, 1H, 5′-H), 7.63 – 7.55 (m, 1H, 7′-H), 7.33 (d, J = 8.5 Hz, 2H, Ph), 7.23 (t, J = 6.9 Hz, 1H, 6′-H), 7.11 (t, J = 6.9 Hz, 1H, Ph′), 6.90 (d, J = 7.7 Hz, 1H, Ph′), 6.62 (d, J = 8.1 Hz, 1H, Ph′), 6.55 (t, J = 7.4 Hz, 1H, Ph′). ^{13}C NMR (101 MHz, DMSO-d_6) δ 163.92, 156.24, 154.88, 151.79, 151.38, 148.20, 147.03, 146.89, 142.57, 130.66, 130.58, 130.43, 128.80, 127.08, 122.73, 120.92, 119.56, 117.83, 117.36, 115.83, 114.27. MS (EI): m/z 451([M]$^+$, 100%). Anal. calcd. for $C_{23}H_{13}N_7O_4$: C, 61.20; H, 2.90; N, 21.72. Found: C, 61.01; H, 2.91; N, 21.81.

5-(2-(2-Hydroxyphenyl)imidazo[1,2-*a*]pyridin-3-yl)-6-(thien-2-yl)[1,2,5]oxadiazolo[3,4-*b*]pyrazine 3o.

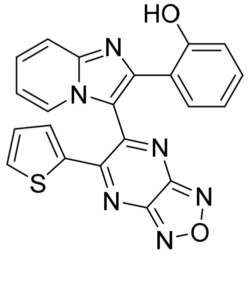

The residue was purified by column chromatography on silica gel eluting with mixture hexane/ethyl acetate 8:2 to give compound as a dark-red solid (74 mg, 36%), mp 237-239 °C. R_f = 0.1. ^{1}H NMR (400 MHz, DMSO-d_6) δ 10.04 (s, 1H, OH), 8.65 (d, J = 6.8 Hz, 1H, 8′-H), 7.83 (d, J = 9.0 Hz, 1H, 5′-H), 7.76 (d, J = 7.6 Hz, 1H, thiophene), 7.59 – 7.45 (m, 2H, 7′-H, thiophene), 7.11 (t, J = 7.3 Hz, 1H, 6′-H), 7.08 – 7.00 (m, 2H, Ph′), 6.88 (t, J = 4.4 Hz, 1H, thiophene), 6.78 (t, J = 7.4 Hz, 1H, Ph′), 6.66 (d, J = 8.1 Hz, 1H, Ph′). ^{13}C NMR (101 MHz, DMSO-d_6) δ 157.66, 155.97, 151.84, 151.65, 145.50, 145.34, 139.80, 135.34, 133.16, 130.66, 130.32, 128.74, 127.95, 126.45, 120.08, 119.85, 117.72, 117.26, 116.20, 114.08. MS (EI): m/z 412 ([M]$^+$, 100%). Anal. calcd. for $C_{21}H_{12}N_6O_2S$: C, 61.16; H, 2.93; N, 20.38. Found: C, 61.05; H, 2.94; N, 20.42.

5-Phenyl-6-(2-(4-hydroxyphenyl)imidazo[1,2-*a*]pyridin-3-yl)[1,2,5]oxadiazolo[3,4-*b*]pyrazine 3p. The

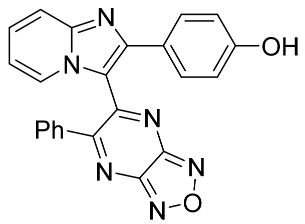

residue was purified by column chromatography on silica gel eluting with mixture hexane/ethyl acetate 7:3 to give compound as a dark-red solid (75 mg, 37%), mp 285-287 °C. R_f = 0.2. ^{1}H NMR (400 MHz, DMSO-d_6) δ 9.60 (s, 1H, OH), 9.03 (d, J = 6.9 Hz, 1H, 8′-H), 7.74 (d, J = 9.0 Hz, 1H, 5′-H), 7.59 – 7.47 (m, 1H, 7′-H), 7.23 (t, J = 7.4 Hz, 1H, 6′-H), 7.16 (t, J = 6.7 Hz, 1H, Ph′), 7.03 (t, J = 7.8 Hz, 2H, Ph), 6.95 (d, J = 8.2 Hz, 4H, Ph′), 6.55 (d, J = 8.5 Hz, 2H, Ph). ^{13}C NMR (101 MHz, DMSO-d_6) δ 165.18, 158.59, 155.16, 151.18, 152.06, 150.34, 146.80, 137.10, 130.50, 130.28, 128.62, 128.52, 127.65, 126.78, 124.91, 117.34, 117.22, 115.66, 113.89. MS (EI): m/z 406 ([M]$^+$, 100%). Anal. calcd. for $C_{23}H_{14}N_6O_2$: C, 67.97; H, 3.47; N, 20.68. Found: C, 67.65; H, 3.46; N, 20.76.

5-(2-(4-Hydroxyphenyl)imidazo[1,2-*a*]pyridin-3-yl)-6-(4-nitrophenyl)[1,2,5]oxadiazolo[3,4-*b*]pyrazine

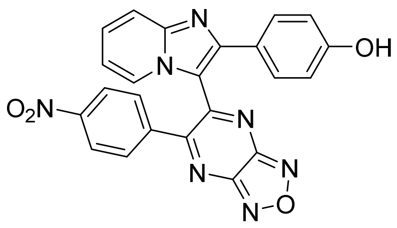

3q. The residue was purified by column chromatography on silica gel eluting with mixture benzene/ethyl acetate 9:1 to give compound as a dark-red solid (92.5 mg, 41%), mp 281-283 °C. Rf = 0.1. ^{1}H NMR (400 MHz, DMSO-d$_6$) δ 9.54 (s, 1H, OH), 9.18 (d, J = 6.9 Hz, 1H, 8′-H), 7.82 (d, J = 8.8 Hz, 2H,Ph), 7.71 (d, J = 9.1 Hz, 2H, 5′-H), 7.61 – 7.49 (m, 1H, 7′-H), 7.23 – 7.09 (m, 3H, 6′-H, Ph),6.79 (d, J = 8.5 Hz, 2H, Ph′), 6.53 (d, J = 8.5 Hz, 2H, Ph′). ^{13}C NMR (101 MHz, DMSO-d$_6$) δ 163.62, 158.98, 154.31, 152.30, 151.83, 151.12, 150.18, 148.05, 147.41, 143.08, 130.55, 129.89, 129.25, 127.16, 124.90, 122.60, 117.35, 116.97, 115.84, 114.18. MS (EI): m/z 451 ([M]$^+$, 100%). Anal. calcd. for $C_{23}H_{13}N_7O_4$: C, 61.20; H, 2.90; N, 21.72. Found: C, 60.99; H, 2.91; N, 21.80.

5-(2-(4-Hydroxyphenyl)imidazo[1,2-*a*]pyridin-3-yl)-6-(thien-2-yl)[1,2,5]oxadiazolo[3,4-*b*]pyrazine 3r.

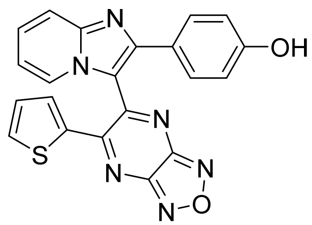

The residue was purified by column chromatography on silica gel eluting with mixture benzene/ethyl acetate 9:1 to give compound as a dark-red solid (68 mg, 33%), mp 300-302 °C. R_f = 0.1. ^{1}H NMR (400 MHz, DMSO-d$_6$) δ 9.60 (s, 1H, OH), 8.78 (d, J = 6.9 Hz, 1H, 8′-H), 7.79 (d, J = 9.0 Hz, 1H, 5′-H), 7.73 (d, J = 4.9 Hz, 1H, thiophene), 7.57 – 7.47 (m, 1H, 7'-H), 7.29 (d, J = 8.5 Hz, 2H, Ph′), 7.09 (t, J = 6.8 Hz, 1H, 6′-H), 6.94 (d, J = 3.0 Hz, 1H, thiophene), 6.90 – 6.81 (m, 1H, thiophene), 6.62 (d, J = 8.6 Hz, 2H, Ph′). ^{13}C NMR (101 MHz, DMSO-d$_6$) δ 158.43, 157.47, 154.28, 152.06, 152.03, 150.18, 149.18, 146.47, 140.07, 135.14, 132.44, 130.04, 128.79, 128.60, 128.34, 126.56, 124.62, 117.29, 116.48, 115.87, 113.95. MS (EI): m/z 412 ([M]$^+$, 100%). Anal. calcd. for $C_{21}H_{12}N_6O_2S$: C, 61.16; H, 2.93; N, 20.38. Found: C, 61.02; H, 2.93; N, 20.47.

2. ¹H NMR spectra of **1a-f** and **2a-c**

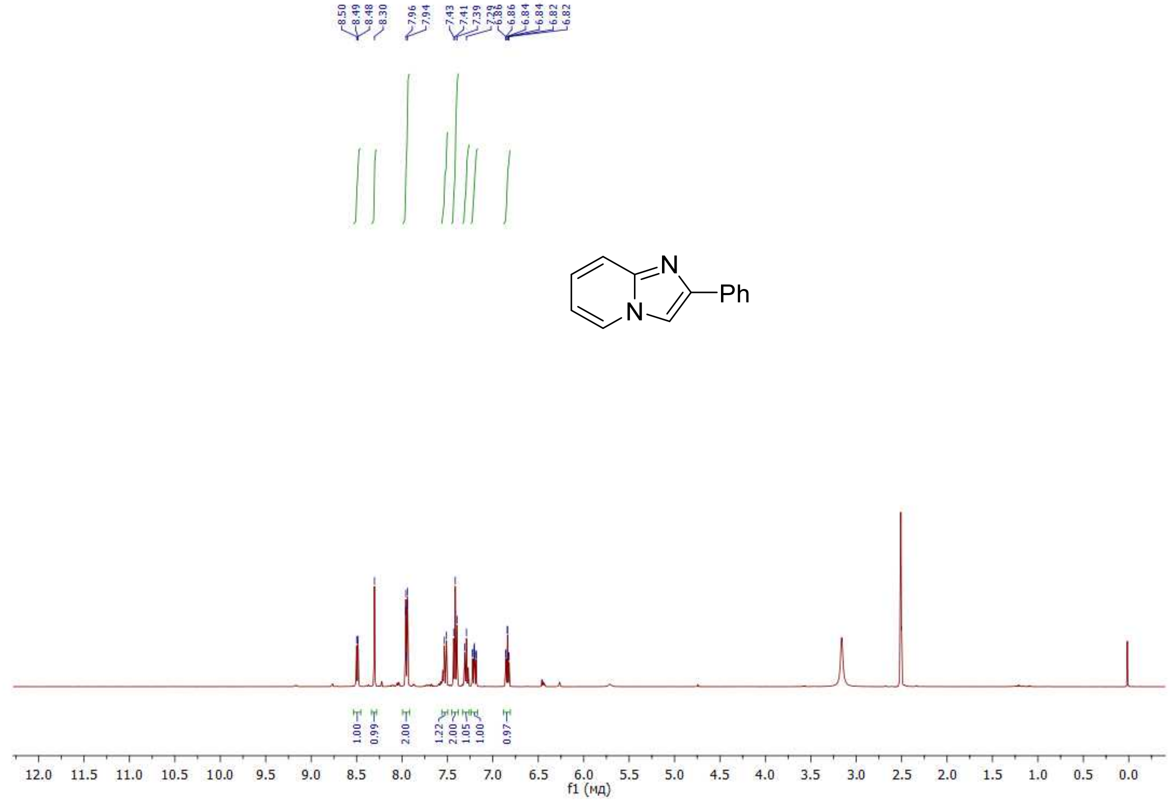

Figure S1. ¹H NMR spectrum of **1a**

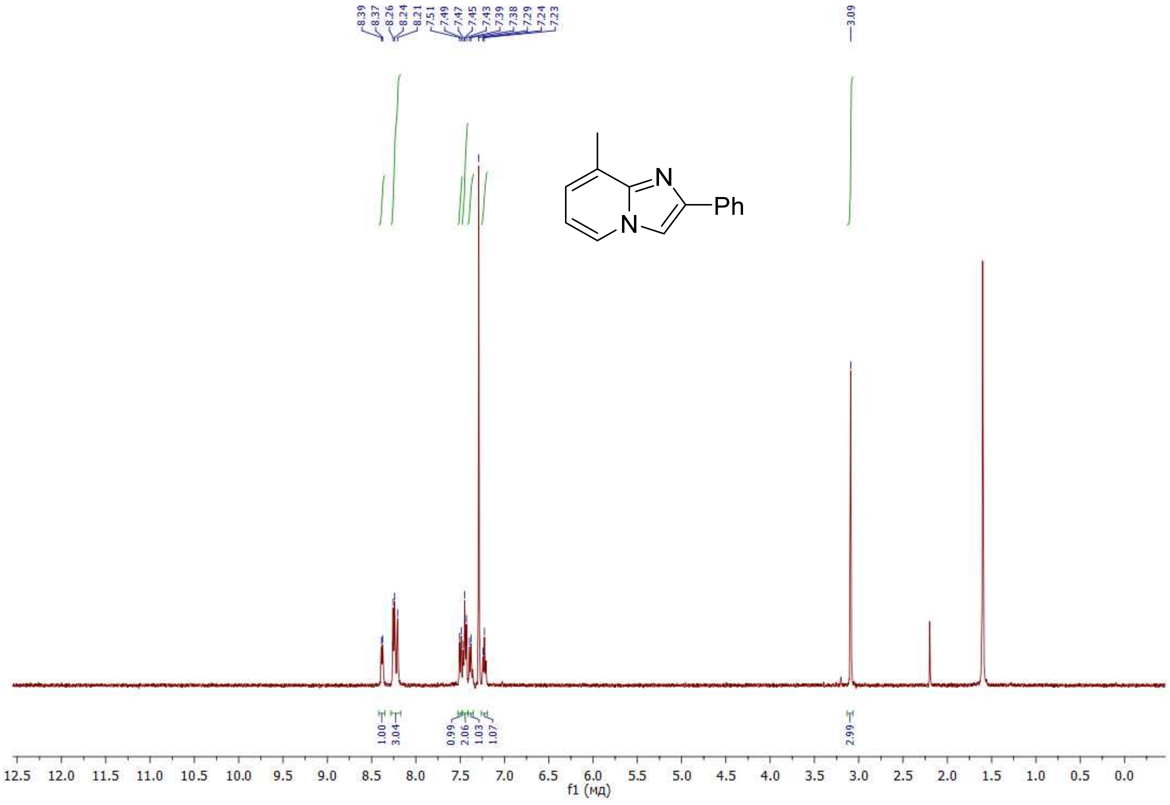

Figure S2. ¹H NMR spectrum of **1b**

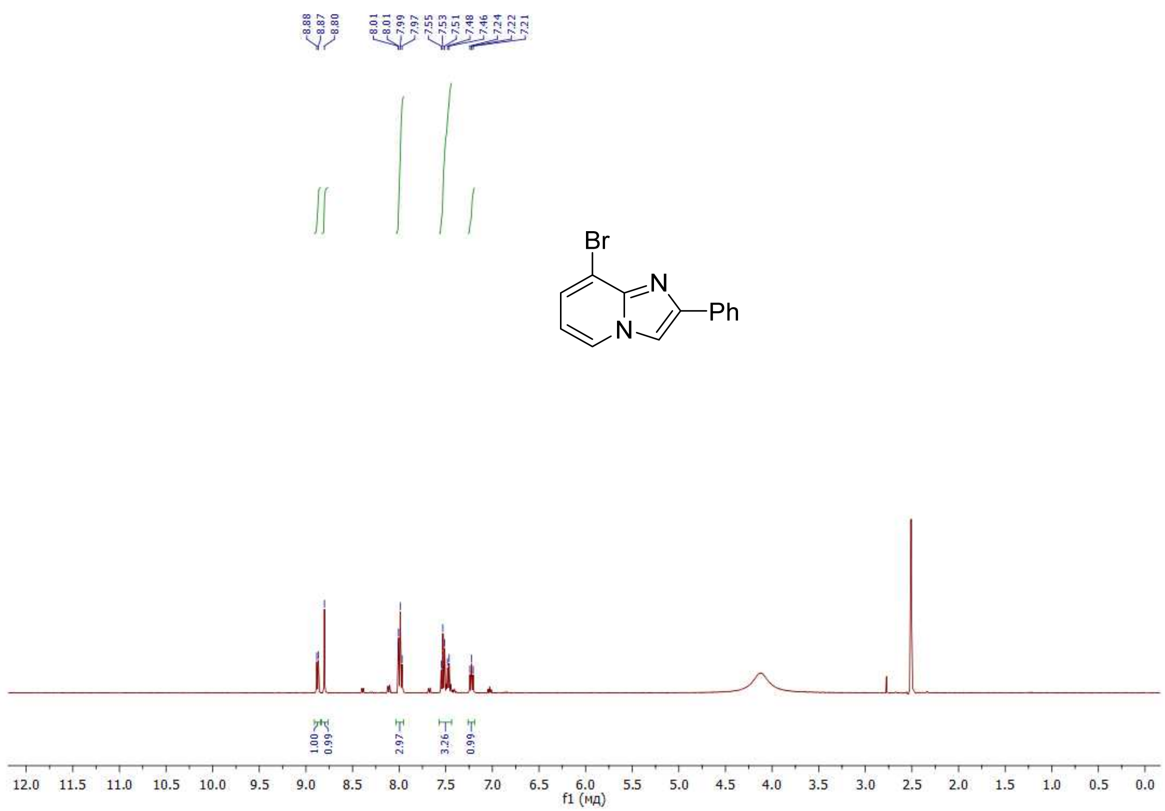

Figure S3. ^{1}H NMR spectrum of **1c**

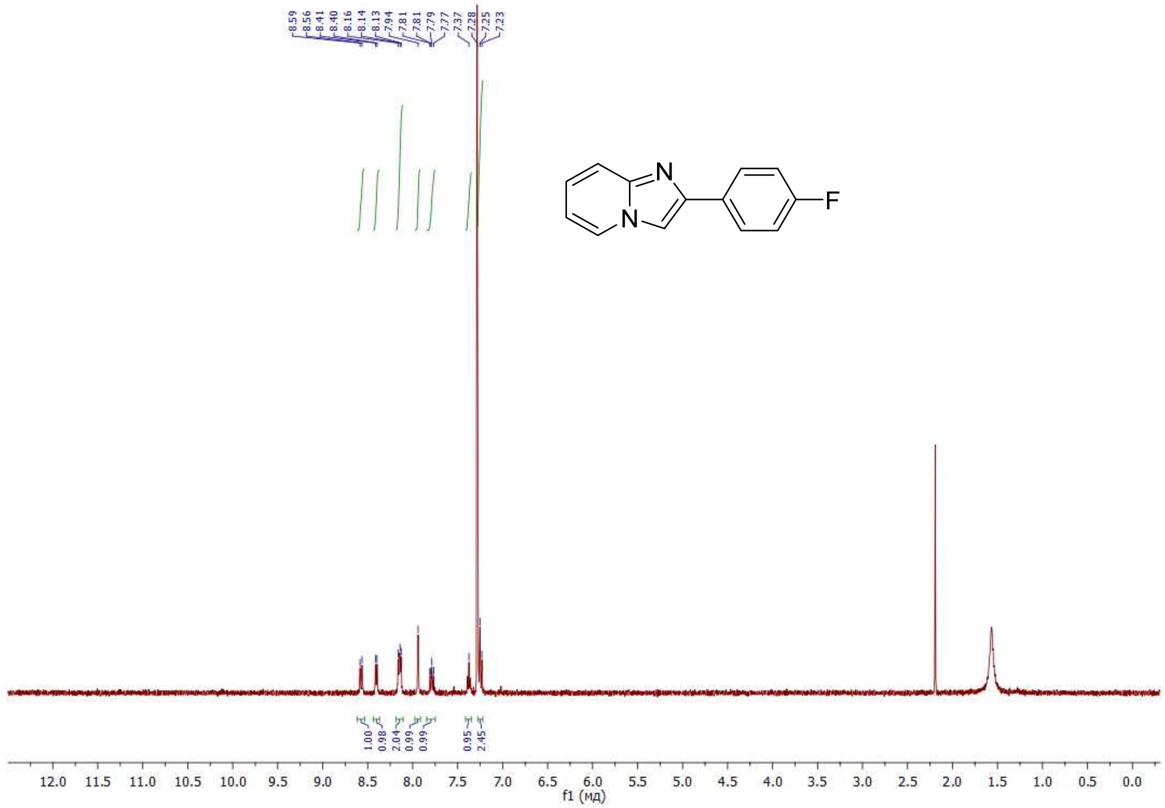

Figure S4. ^{1}H NMR spectrum of **1d**

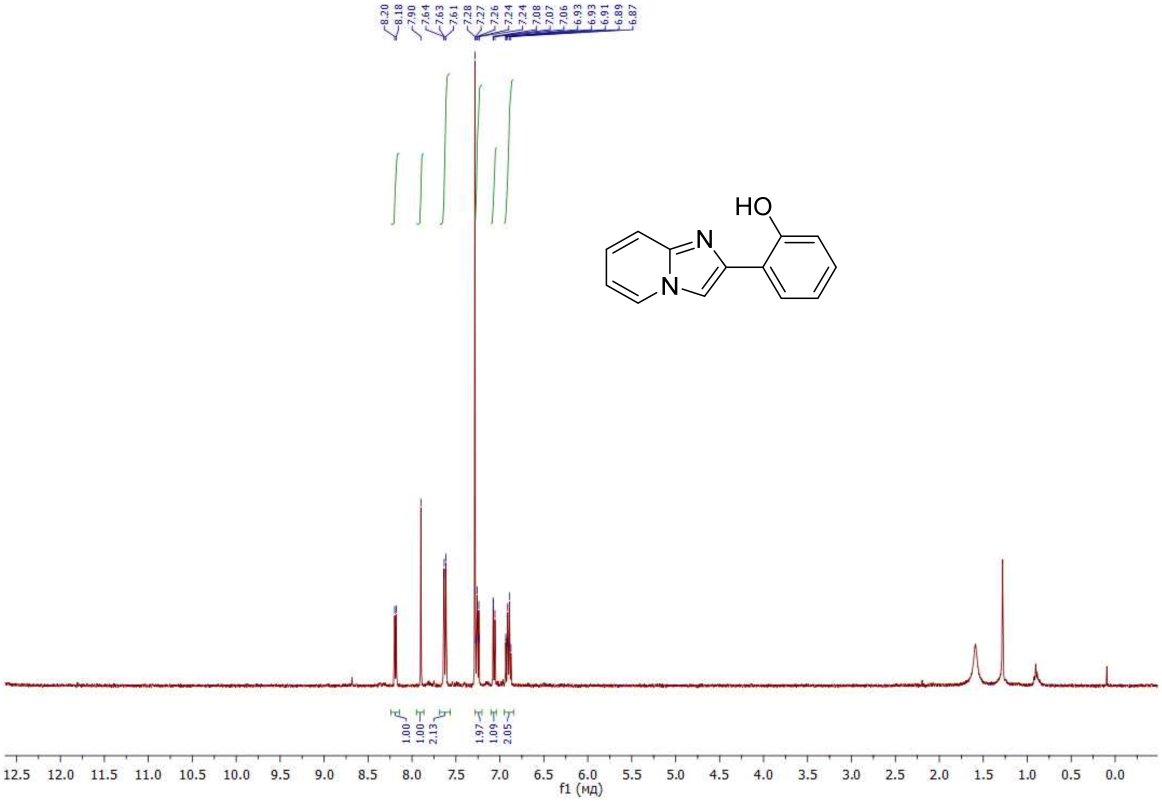

Figure S5. ^{1}H NMR spectrum of **1e**

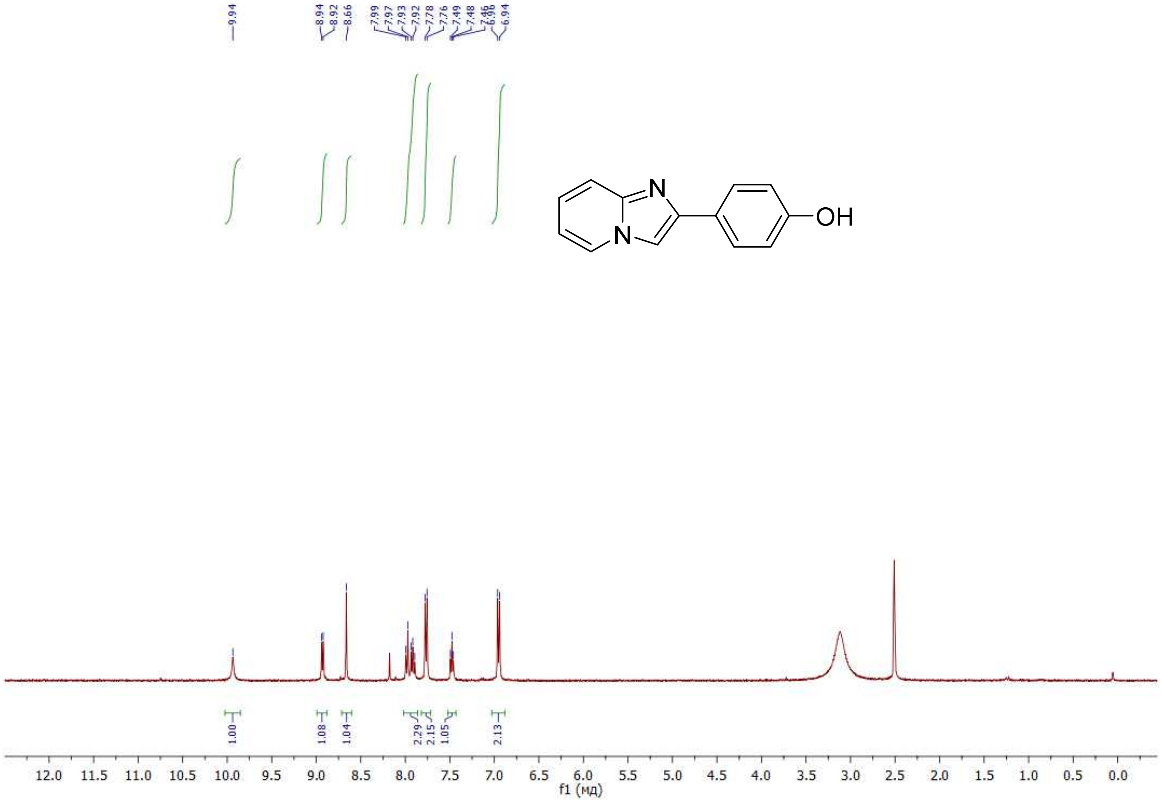

Figure S6. ^{1}H NMR spectrum of **1f**

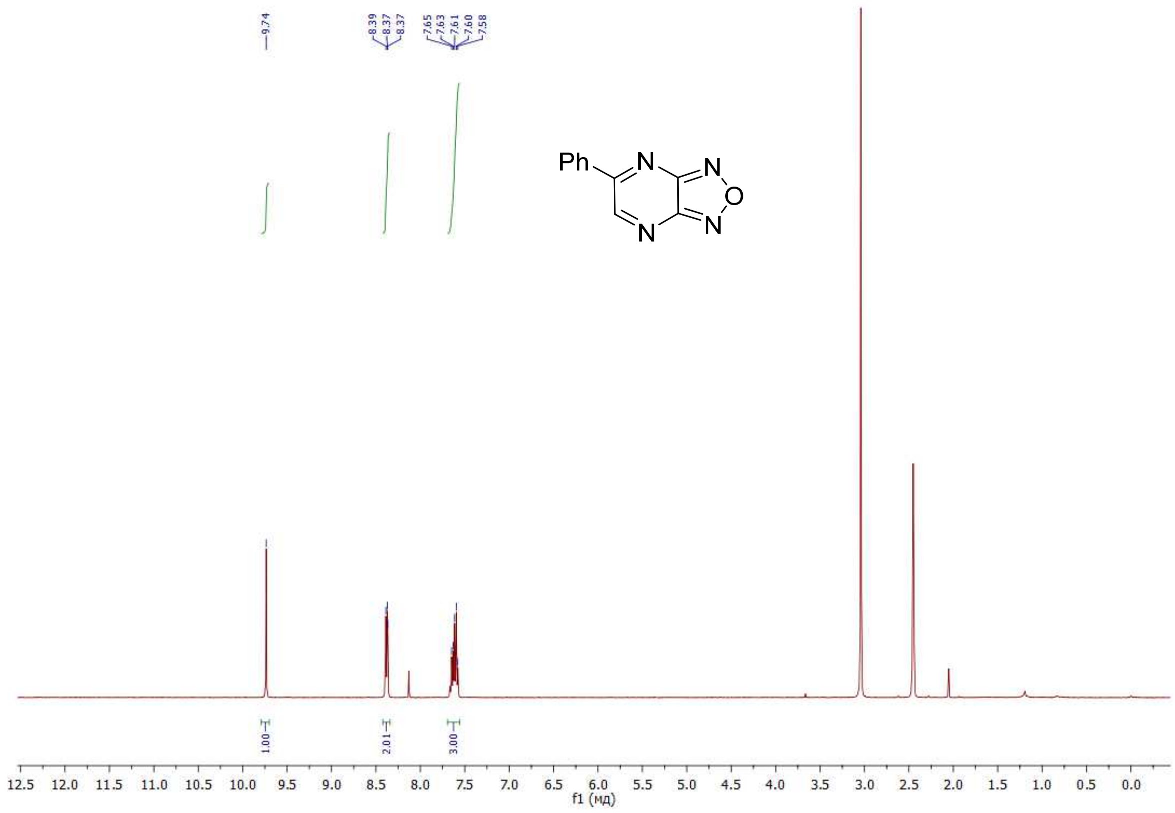

Figure S7. ^{1}H NMR spectrum of **2a**

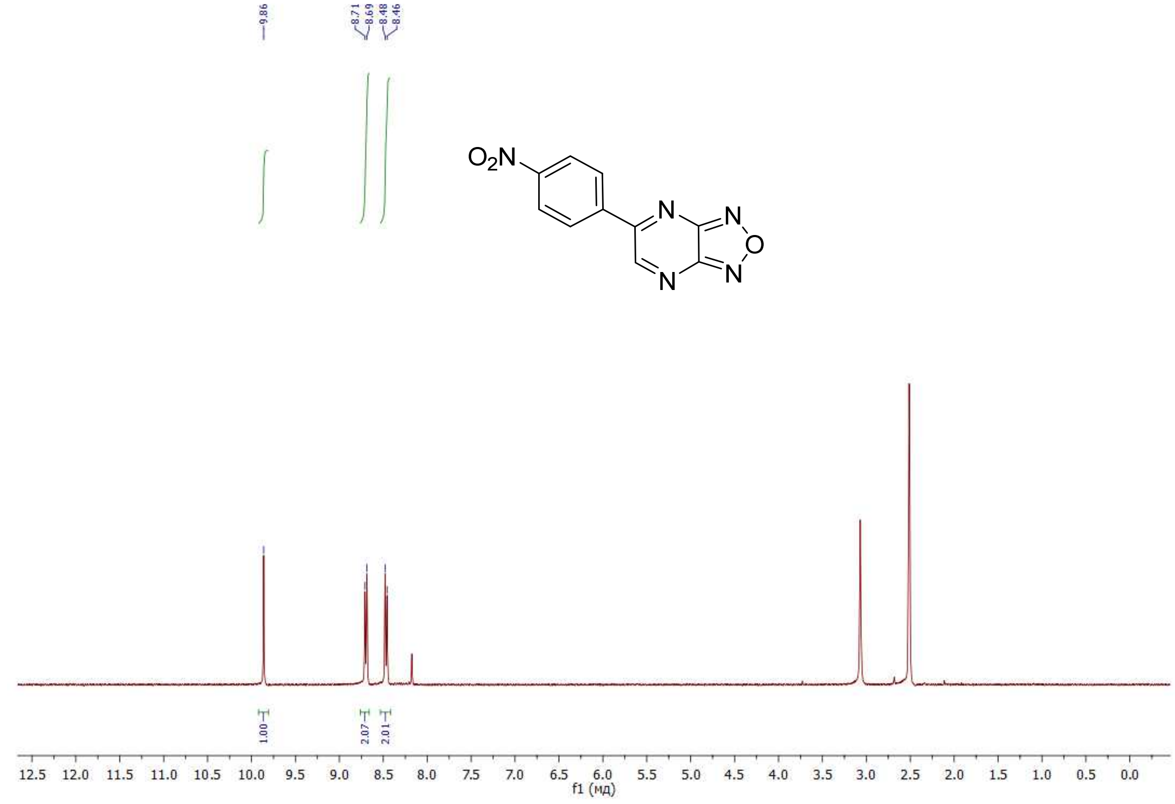

Figure S8. ^{1}H NMR spectrum of **2b**

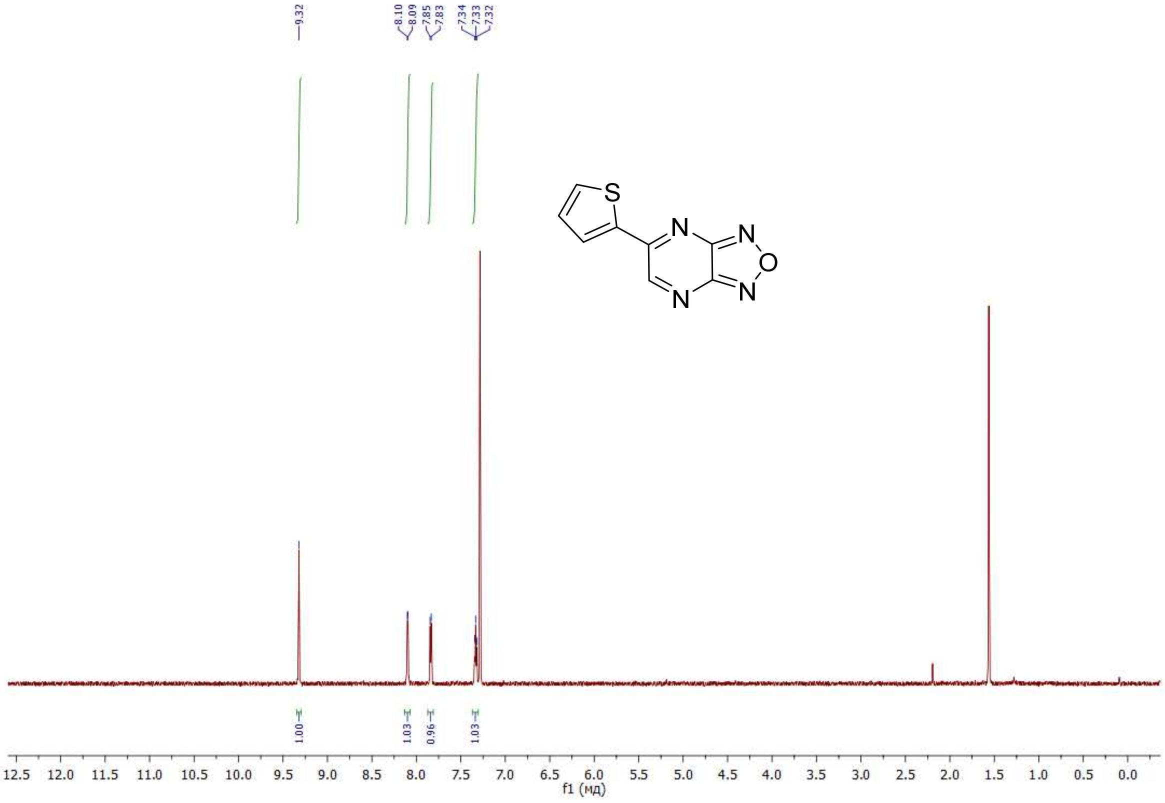

Figure S9. ^{1}H NMR spectrum of **2c**

3. ¹H NMR and ¹³C NMR spectra of **3a-r**

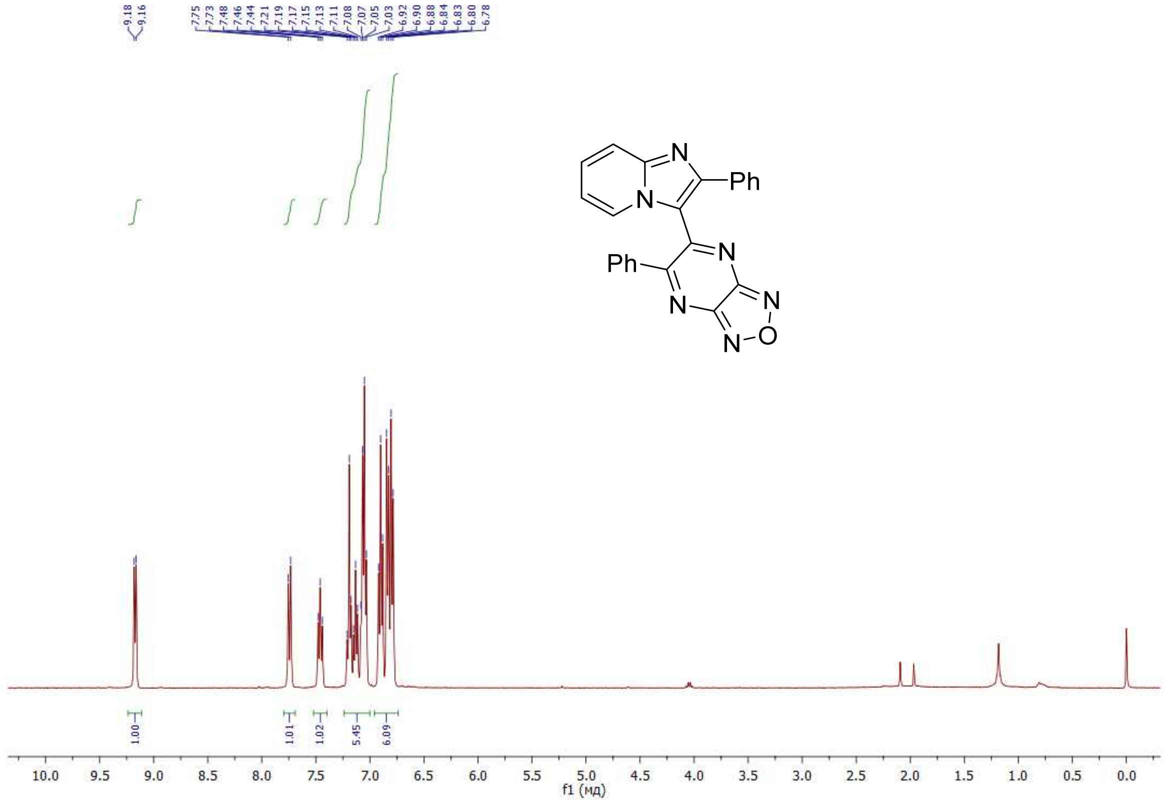

Figure S10. ¹H NMR spectrum of **3a**

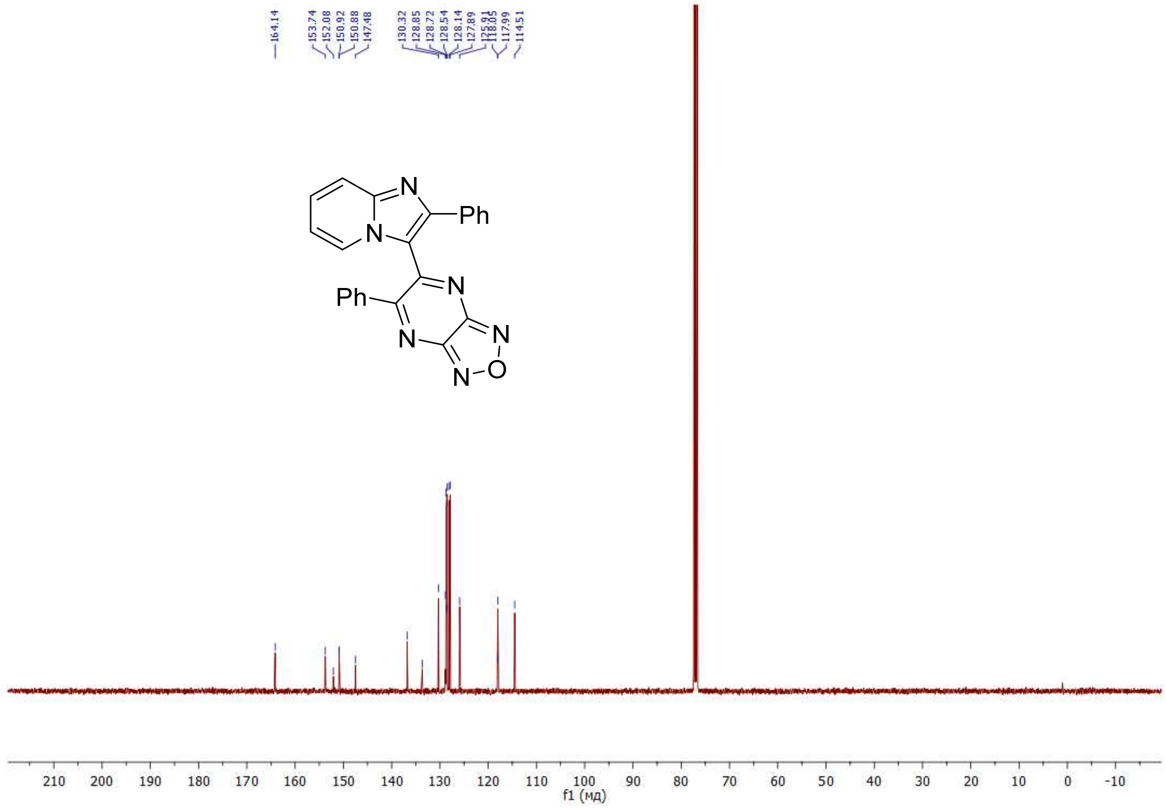

Figure S11. ¹³C NMR spectrum of **3a**

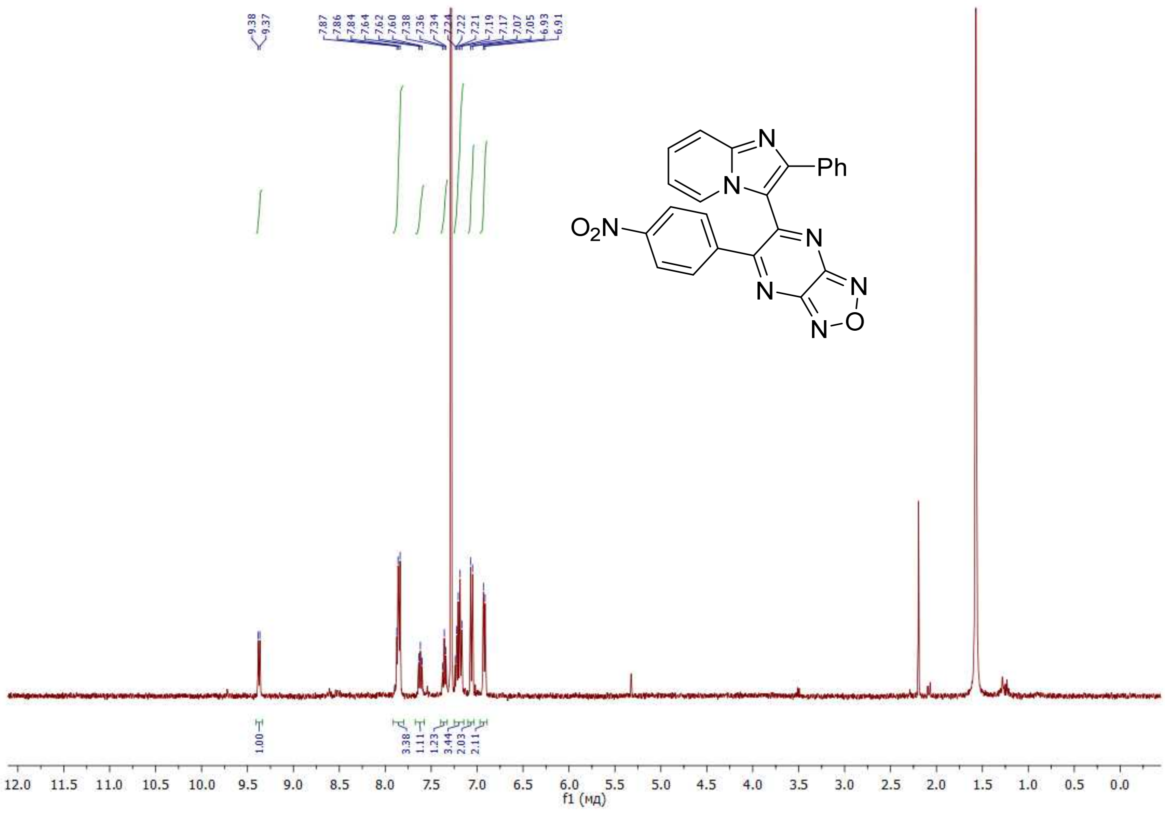

Figure S12. ^{1}H NMR spectrum of **3b**

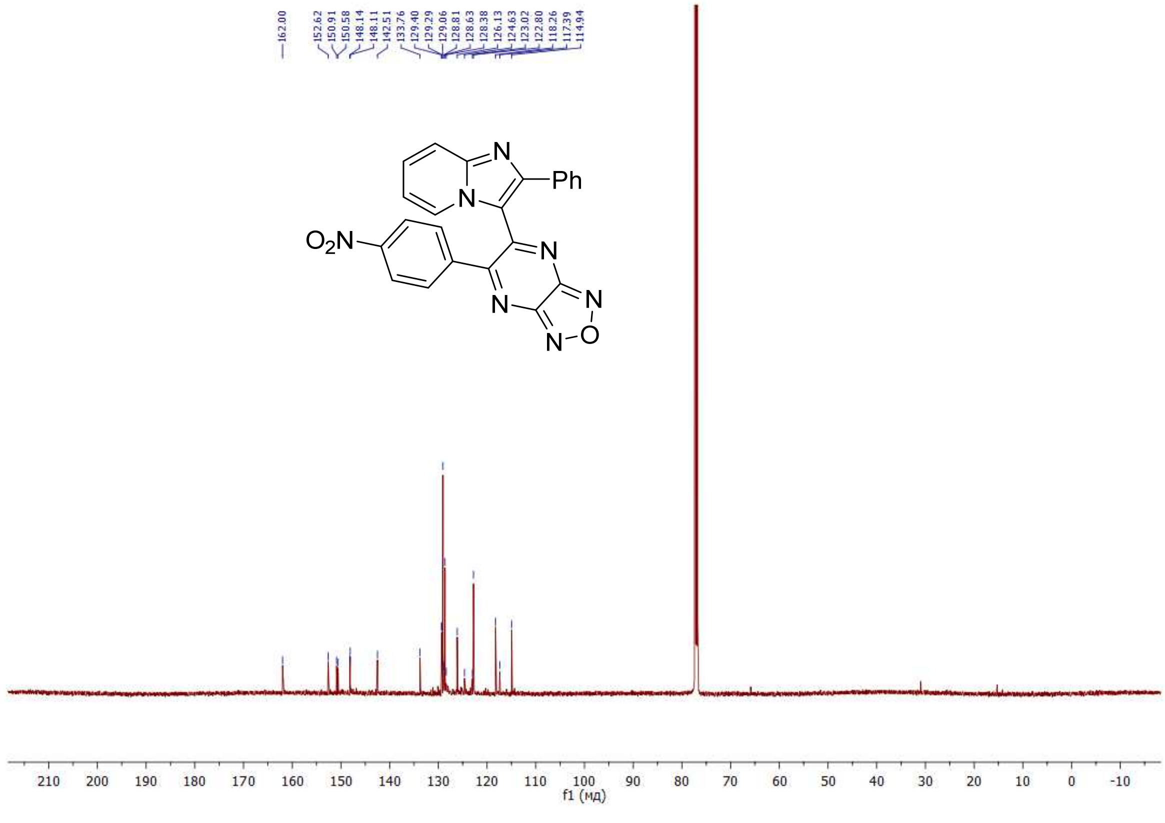

Figure S13. ^{13}C NMR spectrum of **3b**

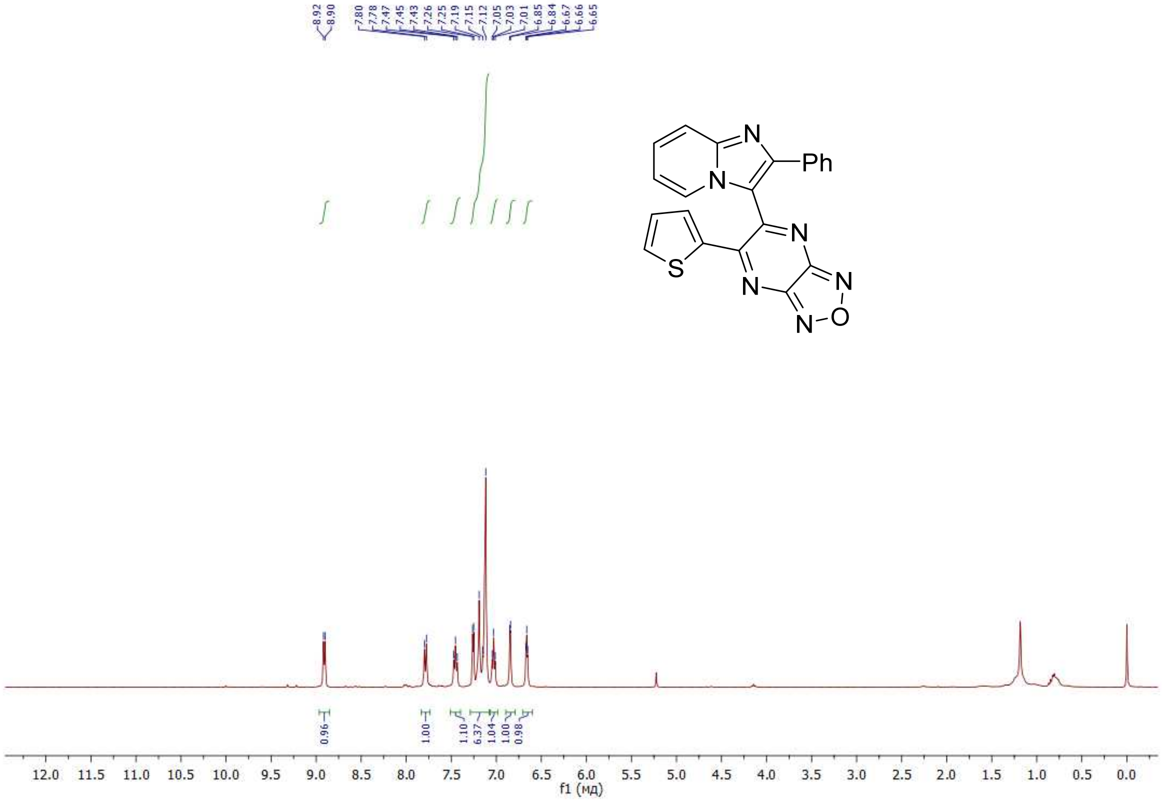

Figure S14. ^{1}H NMR spectrum of **3c**

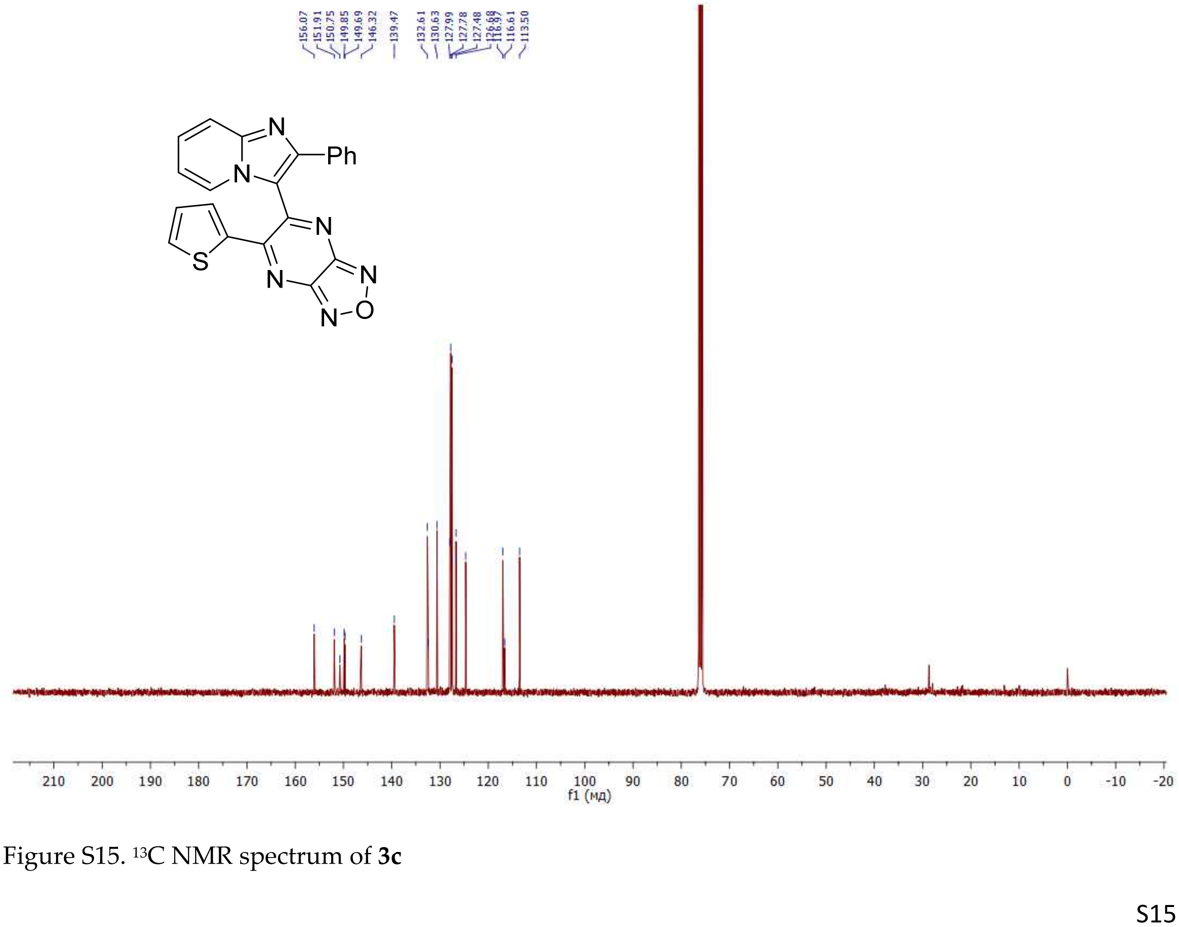

Figure S15. ^{13}C NMR spectrum of **3c**

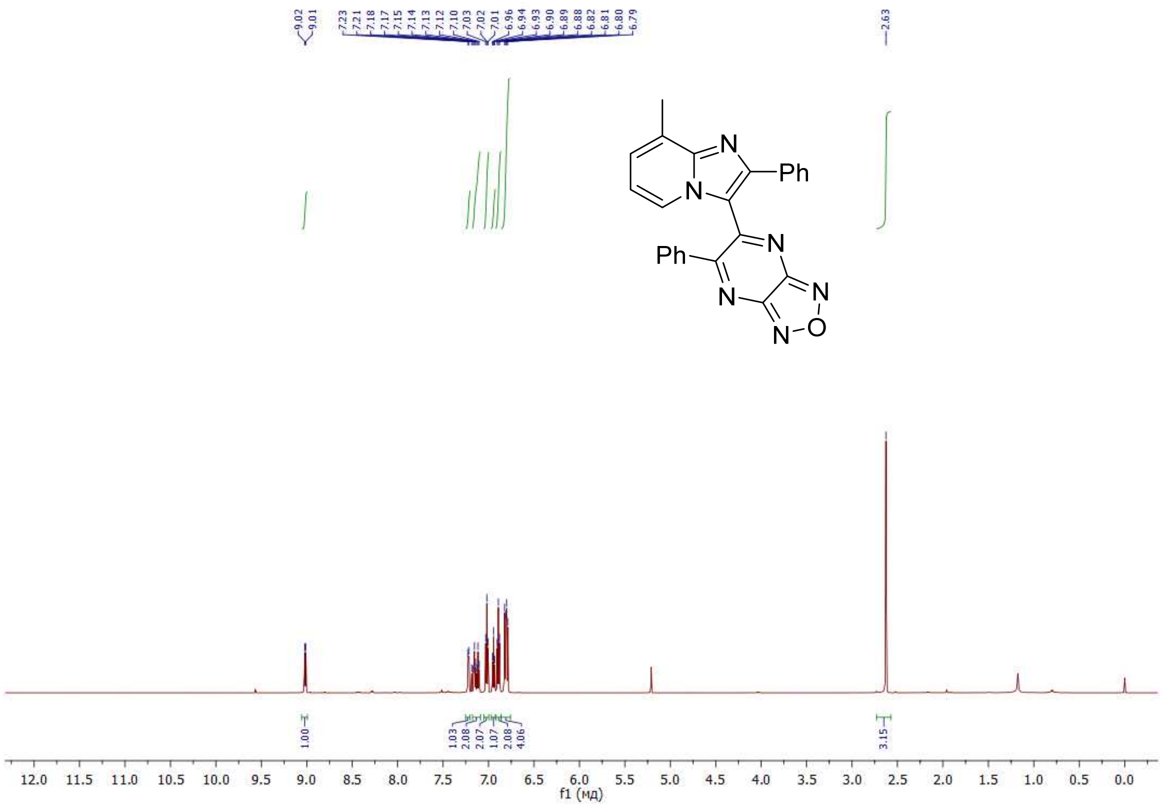

Figure S16. ^{1}H NMR spectrum of **3d**

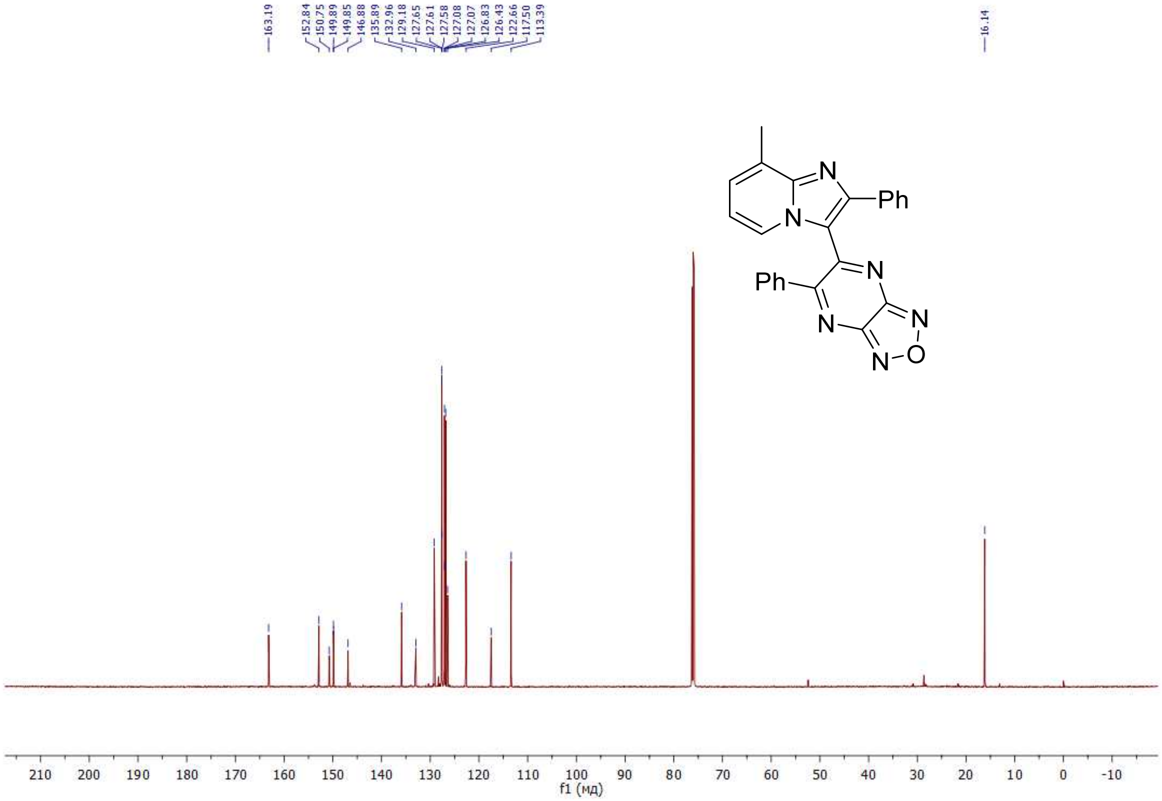

Figure S17. ^{13}C NMR spectrum of **3d**

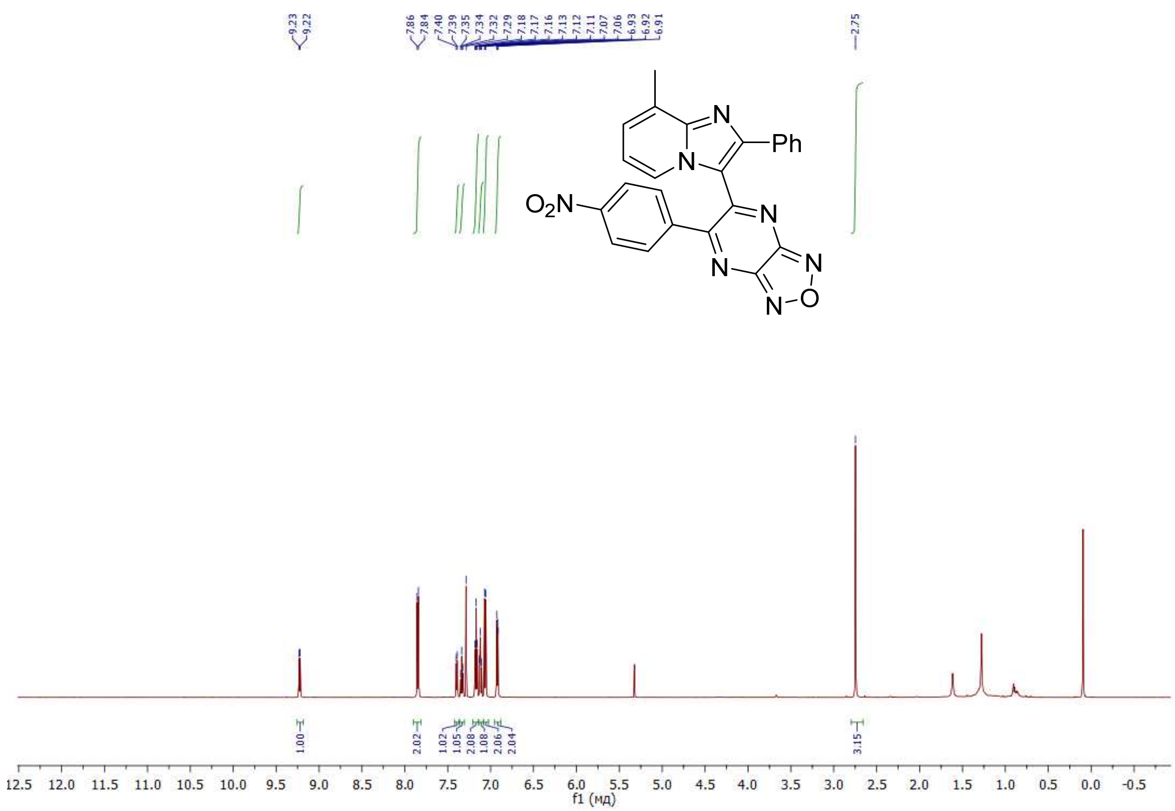

Figure S18. ^{1}H NMR spectrum of **3e**

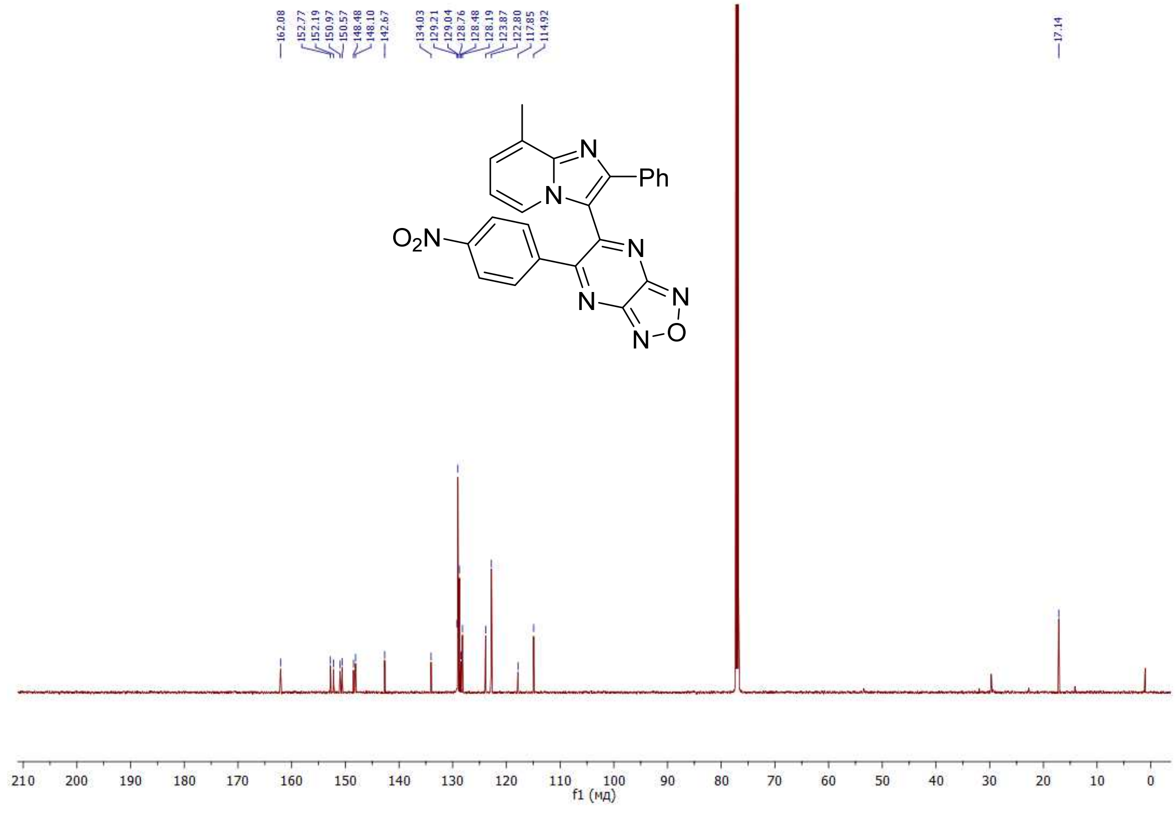

Figure S19. ^{13}C NMR spectrum of **3e**

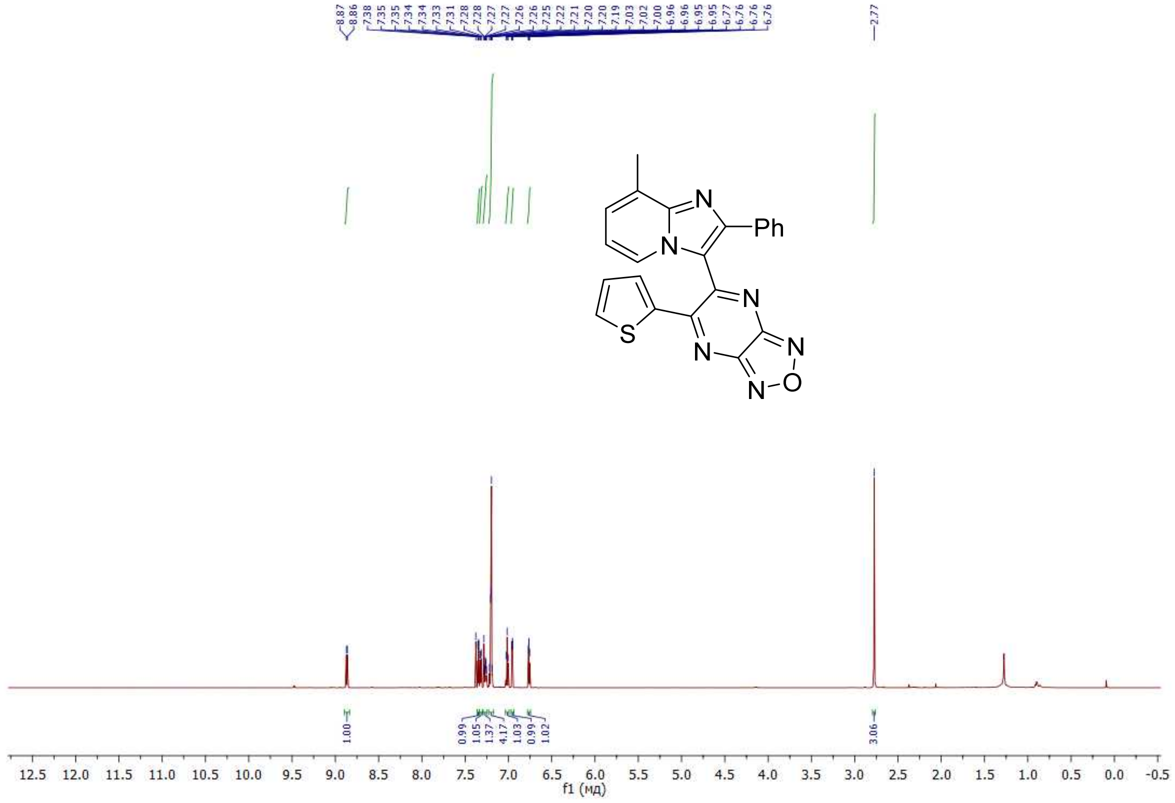

Figure S20. ¹H NMR spectrum of **3f**

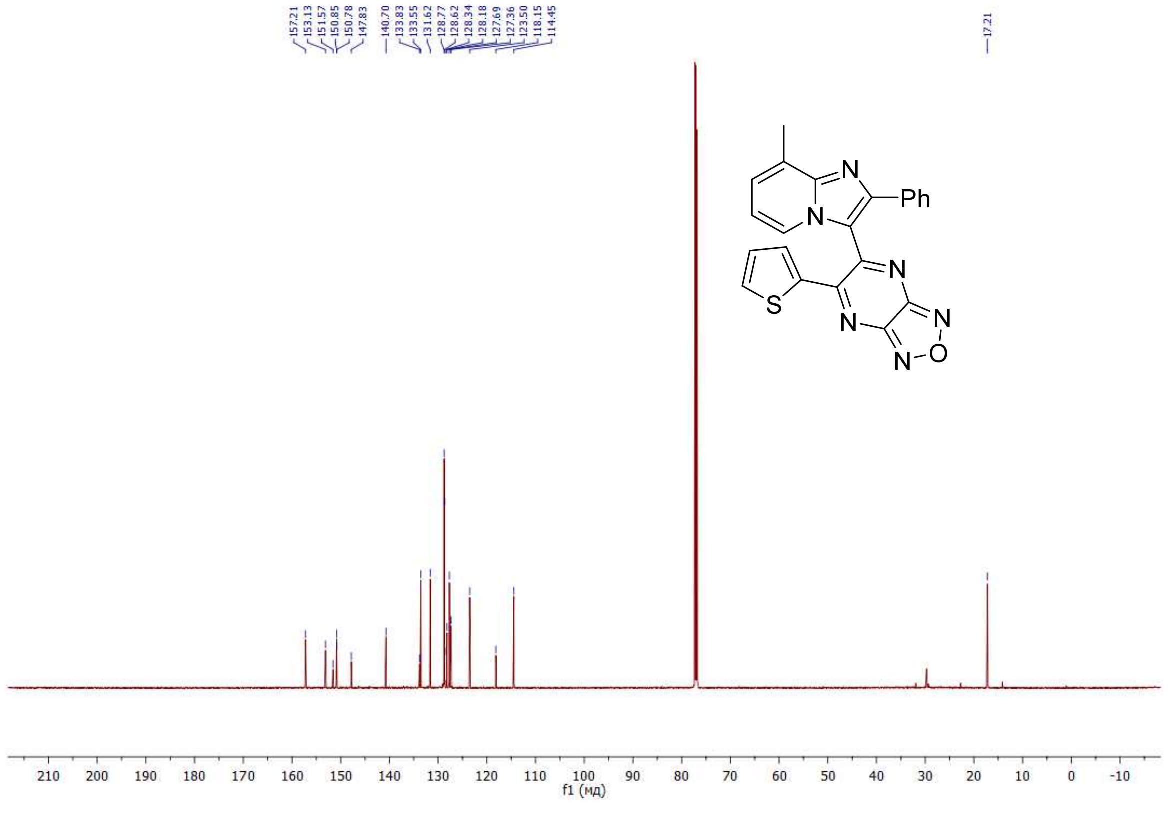

Figure S21. ¹³C NMR spectrum of **3f**

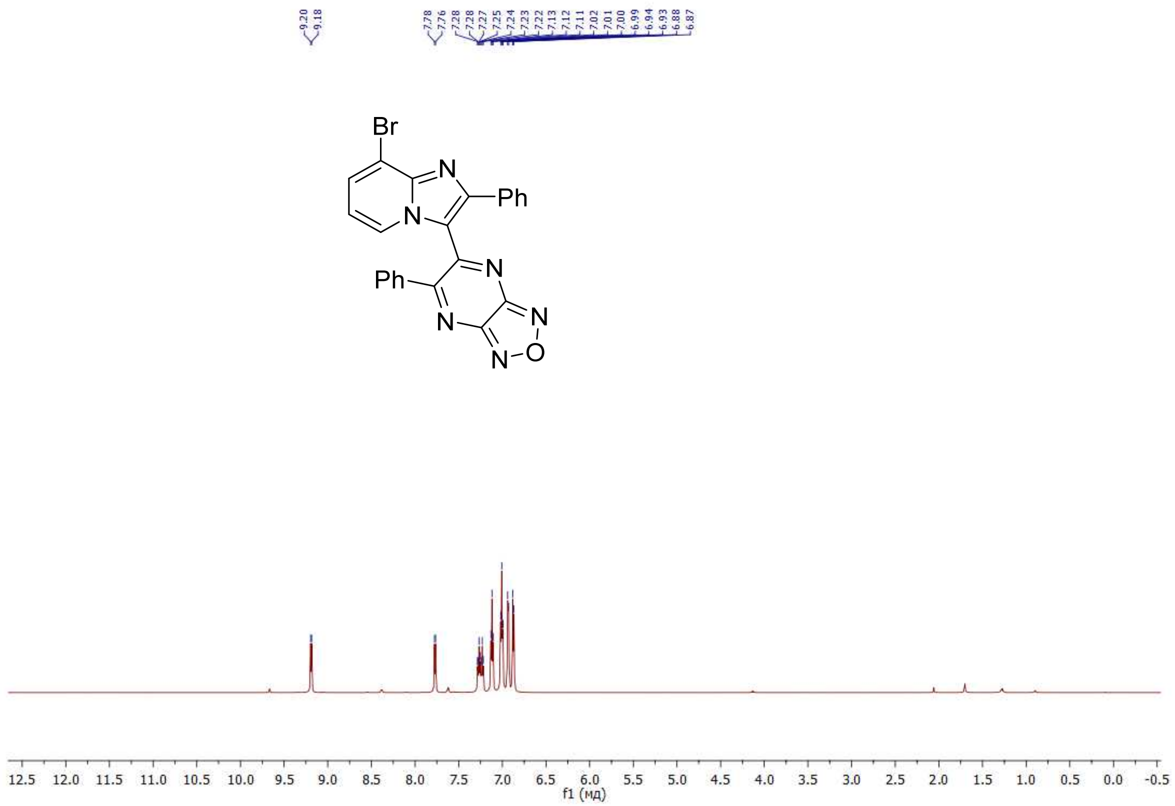

Figure S22. ^{1}H NMR spectrum of **3g**

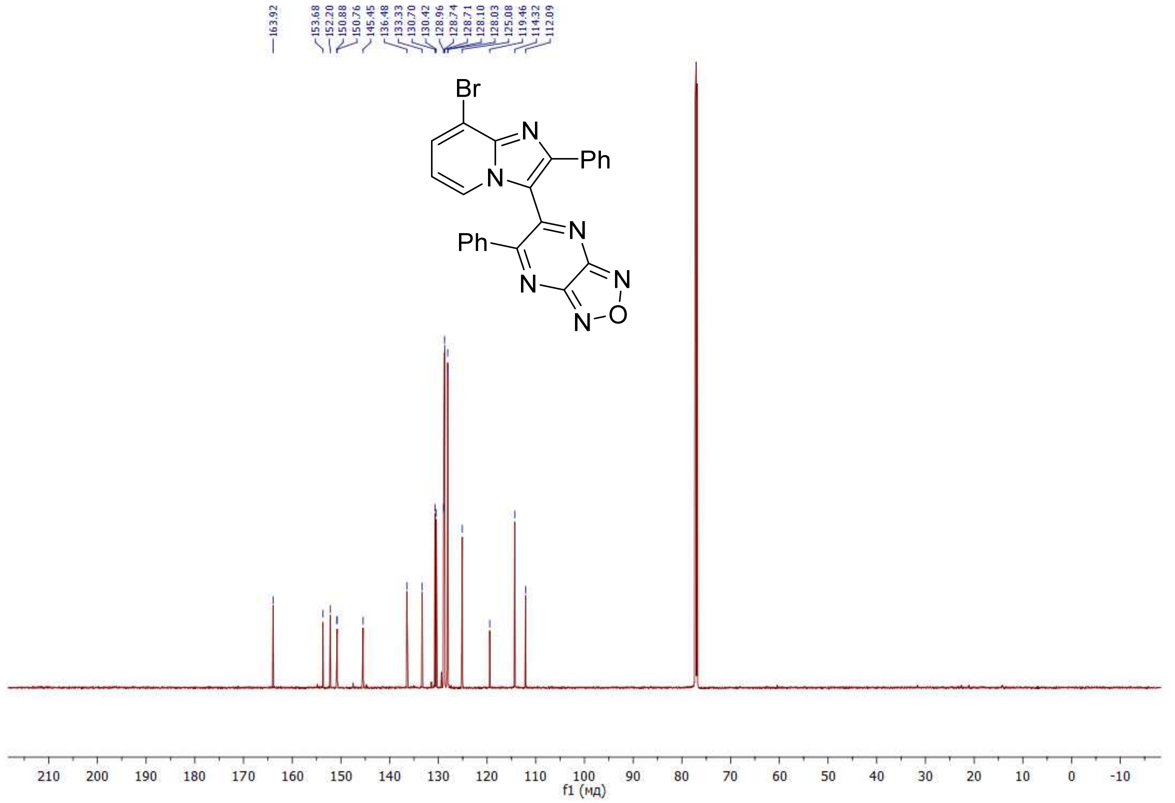

Figure S23. ^{13}C NMR spectrum of **3g**

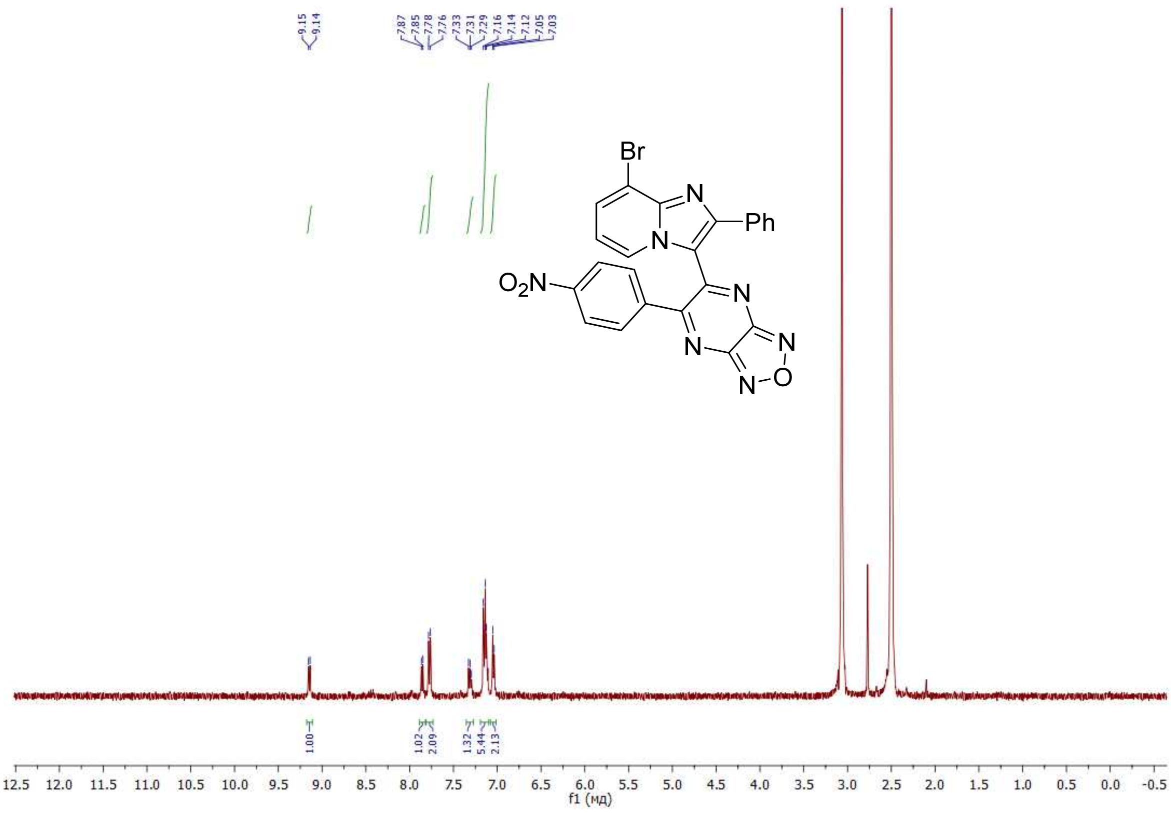

Figure S24. ^{1}H NMR spectrum of **3h**

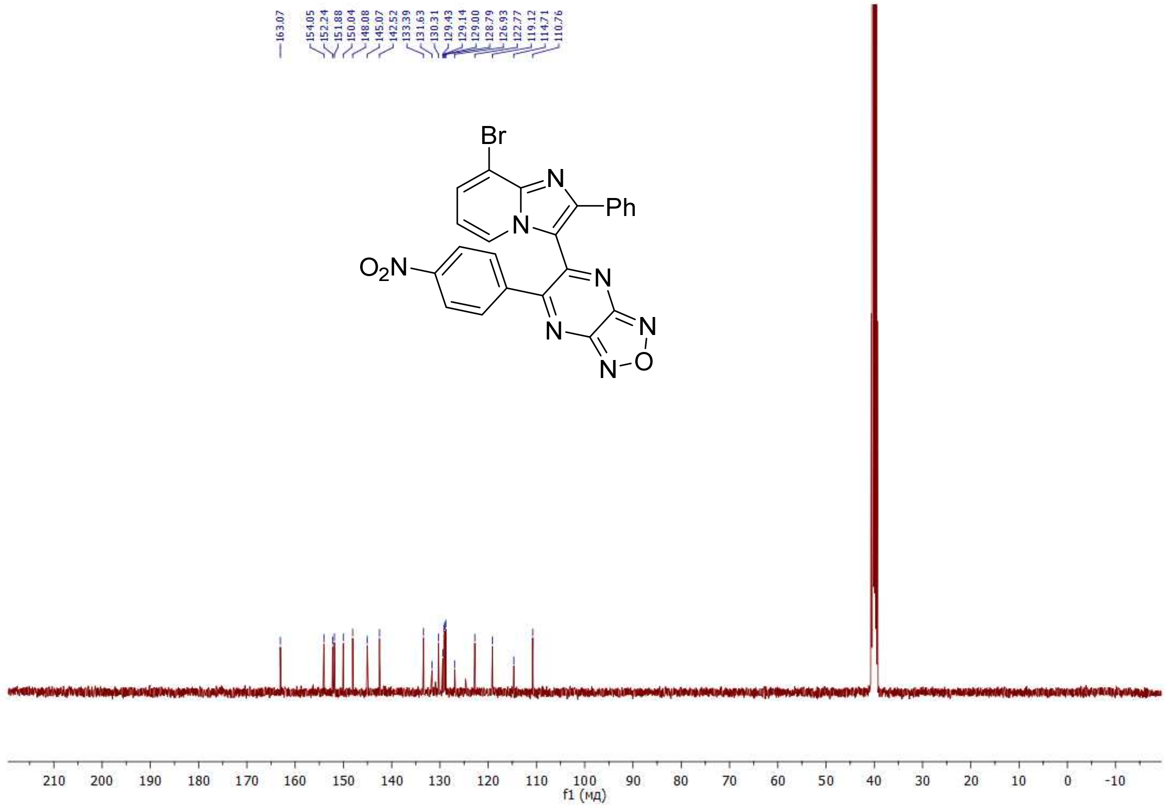

Figure S25. ^{13}C NMR spectrum of **3h**

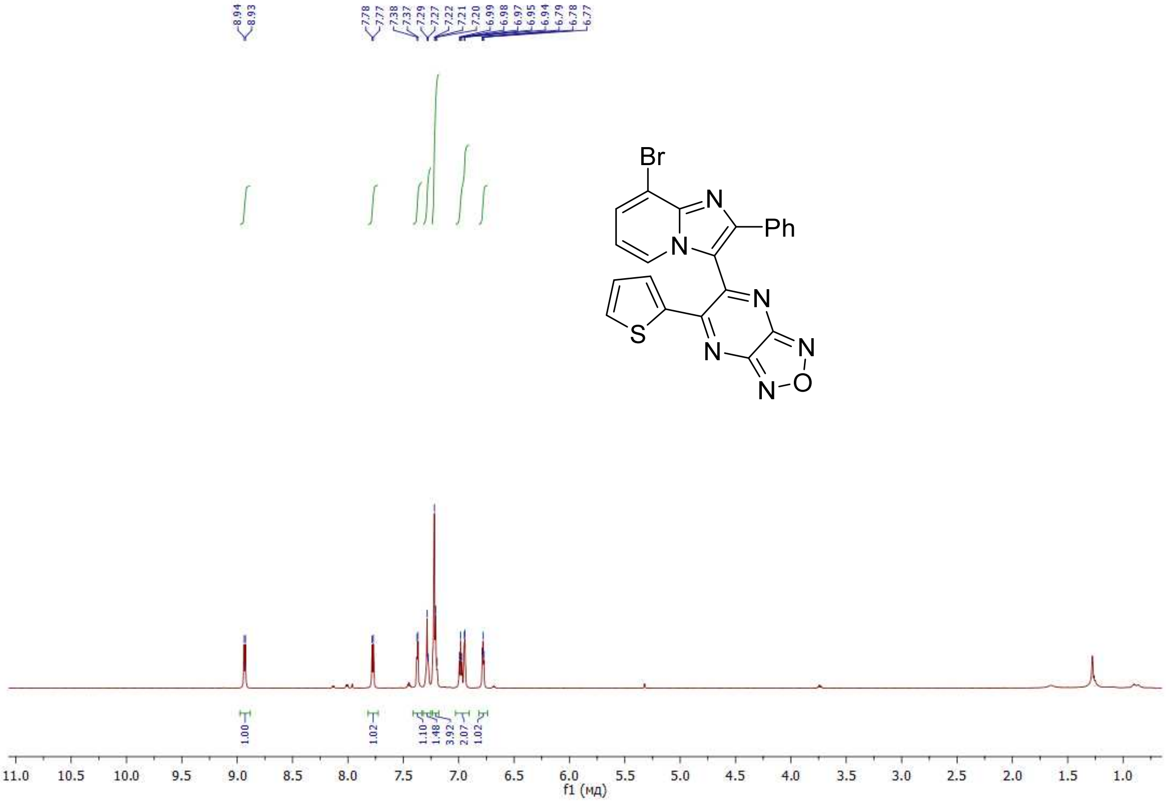

Figure S26. ^{1}H NMR spectrum of **3i**

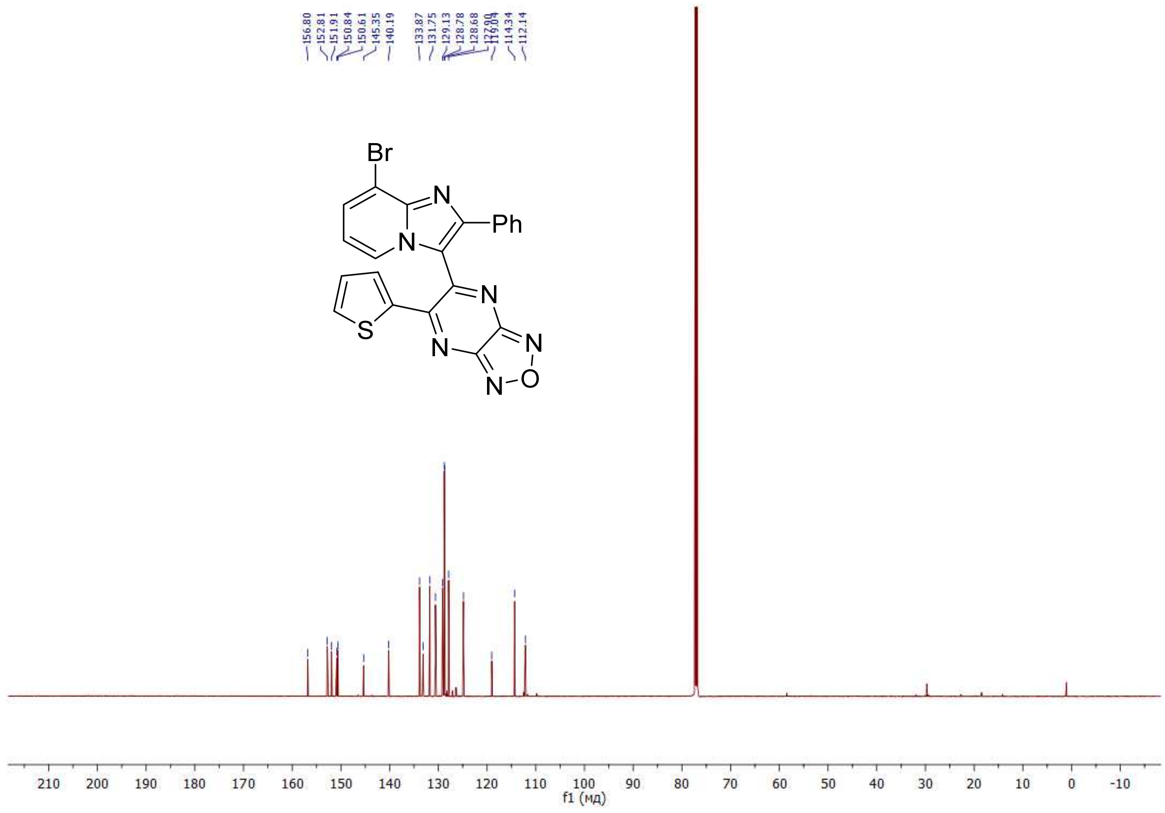

Figure S27. ^{13}C NMR spectrum of **3i**

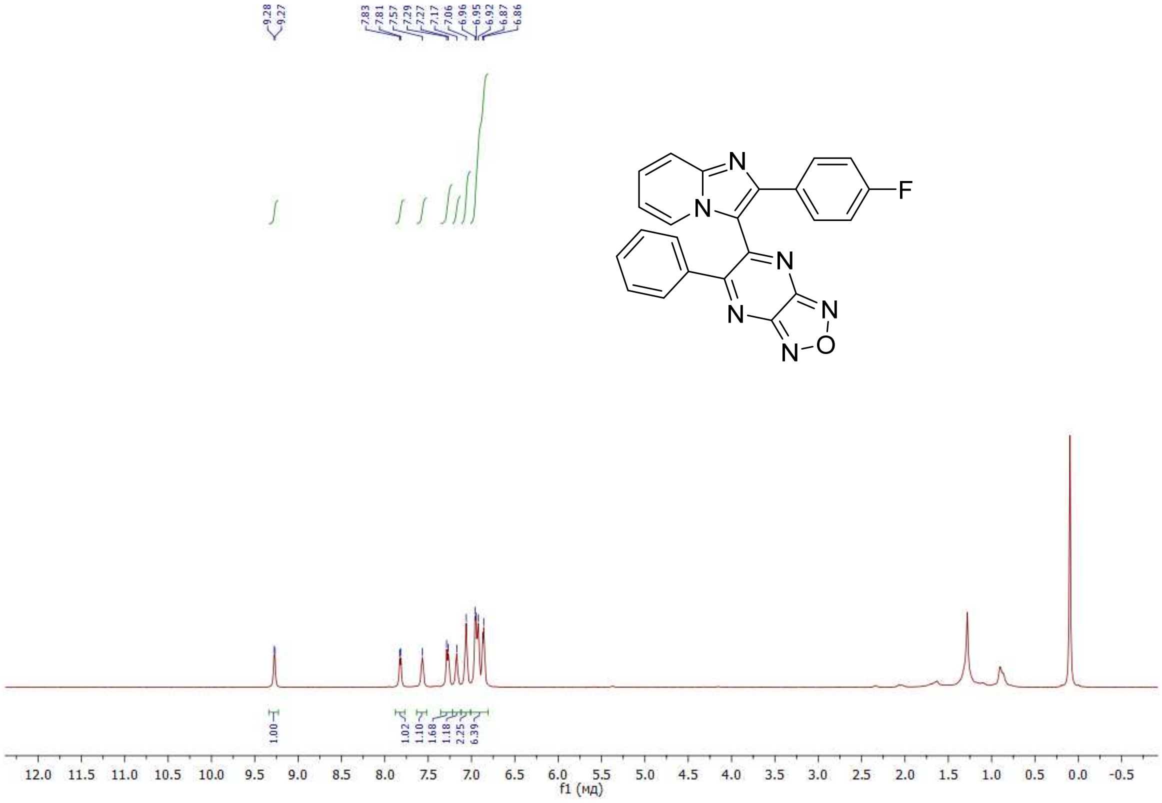

Figure S28. ^{1}H NMR spectrum of **3j**

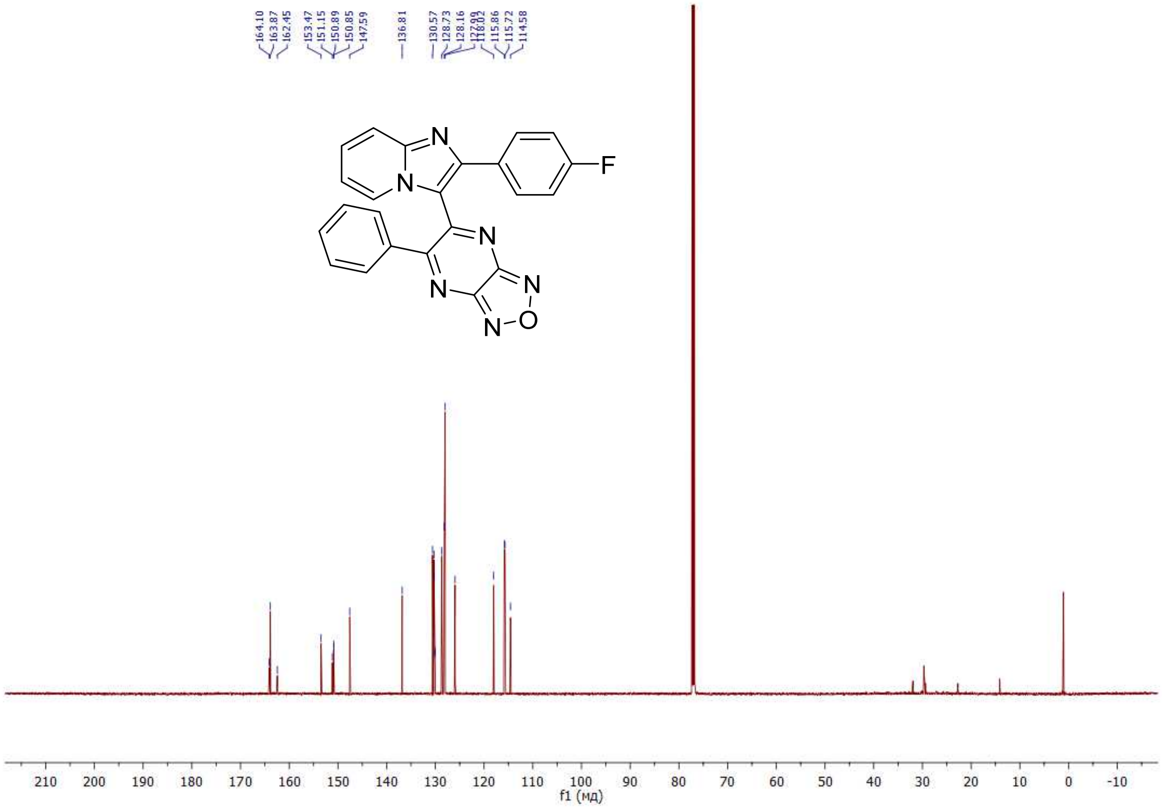

Figure S29. ^{13}C NMR spectrum of **3j**

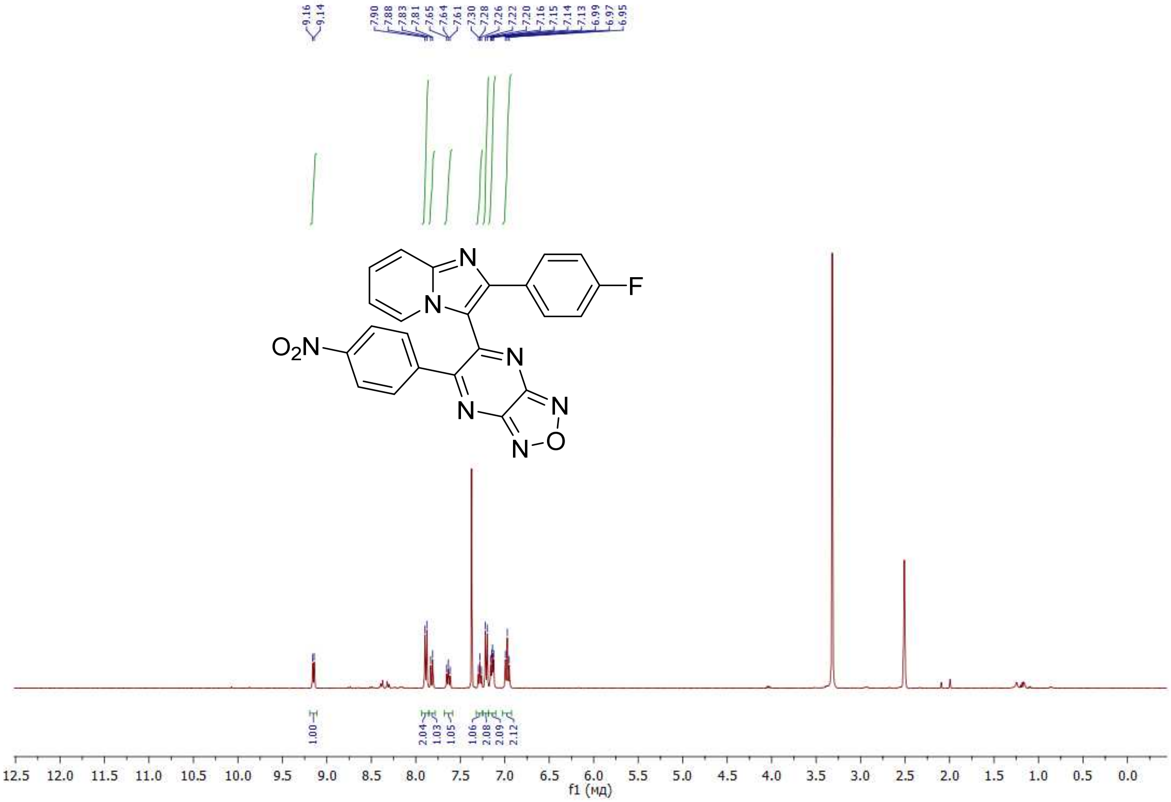

Figure S30. ^{1}H NMR spectrum of **3k**

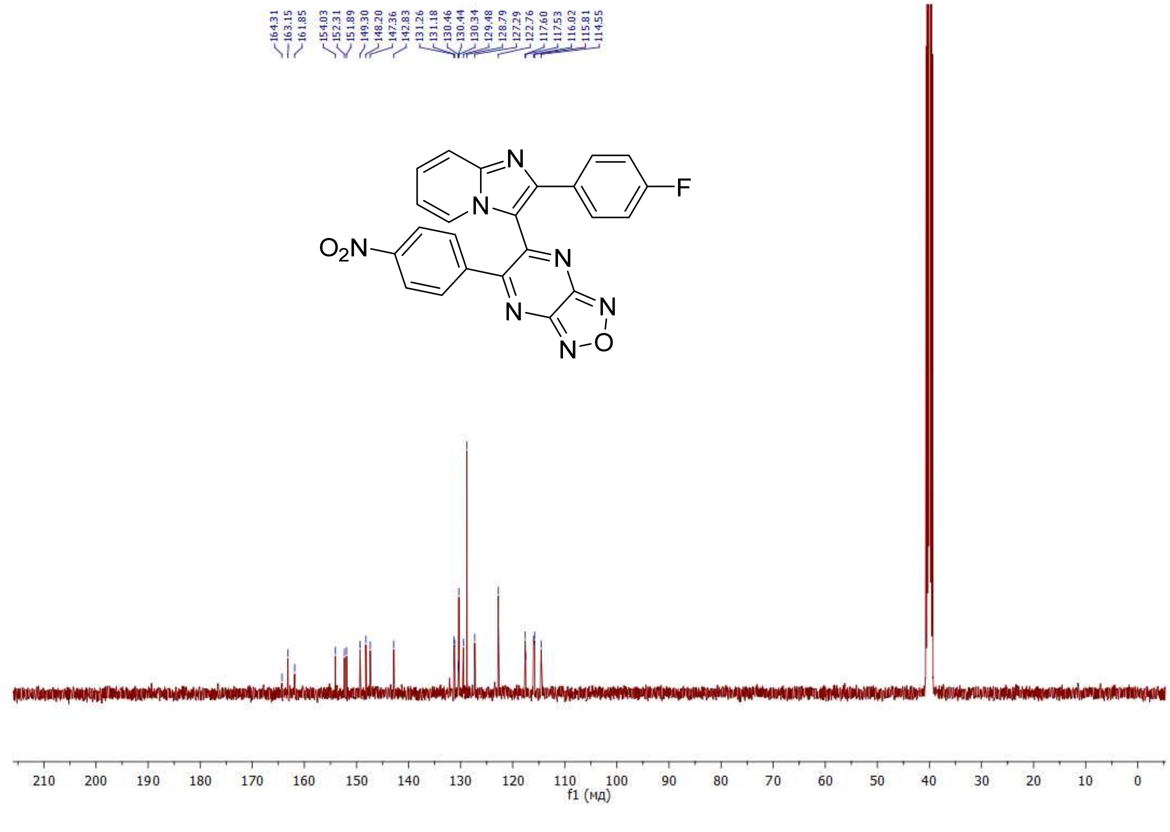

Figure S31. ^{13}C NMR spectrum of **3k**

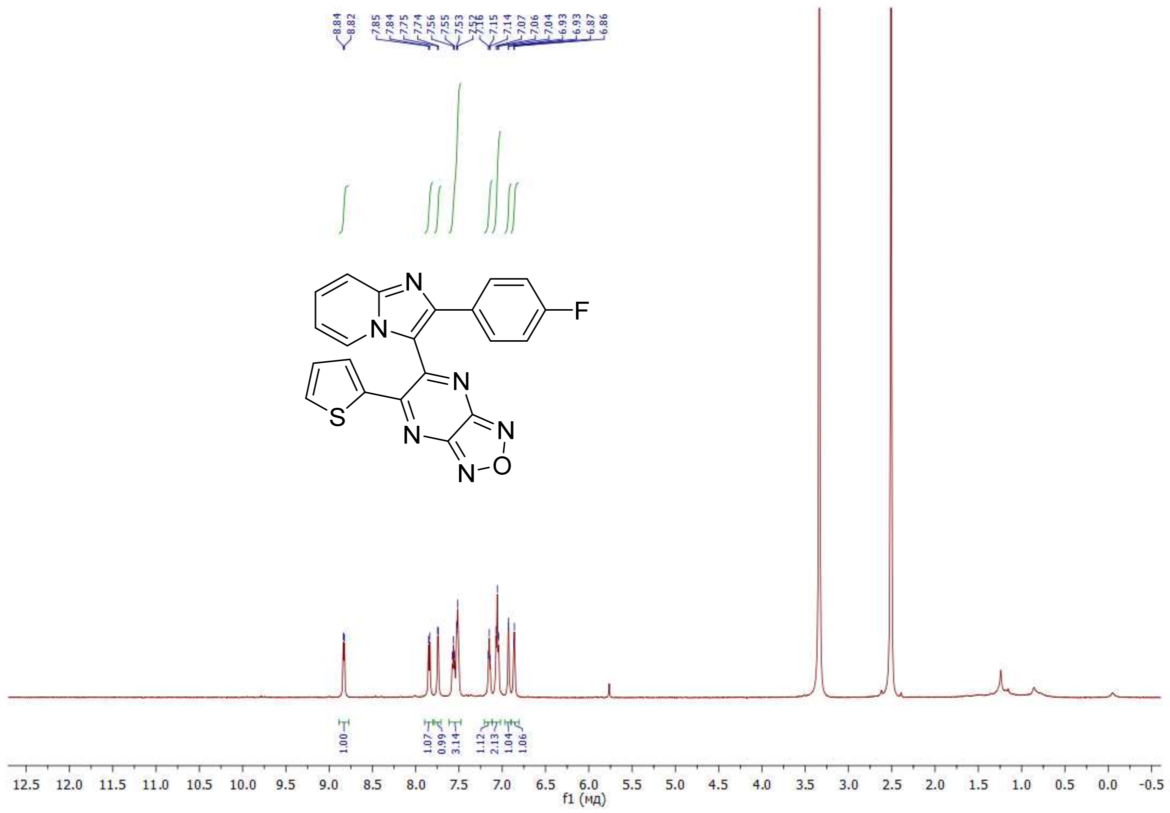

Figure S32. ^{1}H NMR spectrum of **3l**

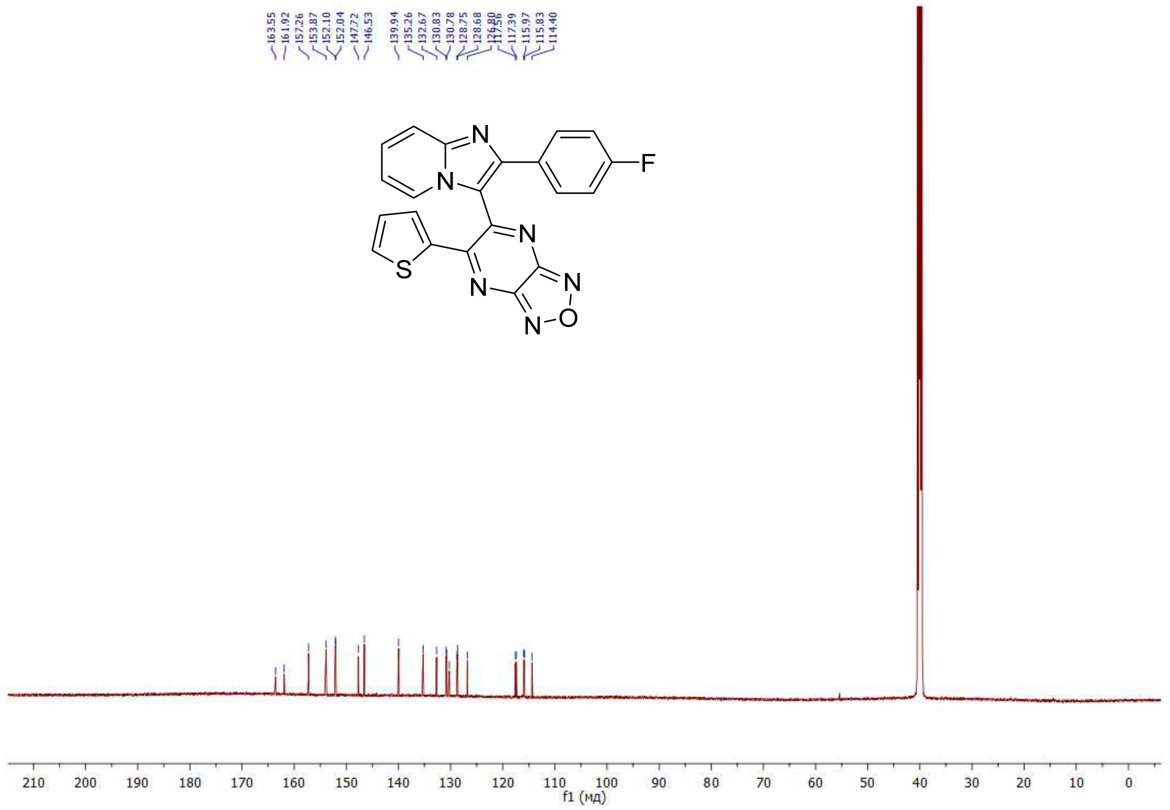

Figure S33. ^{13}C NMR spectrum of **3l**

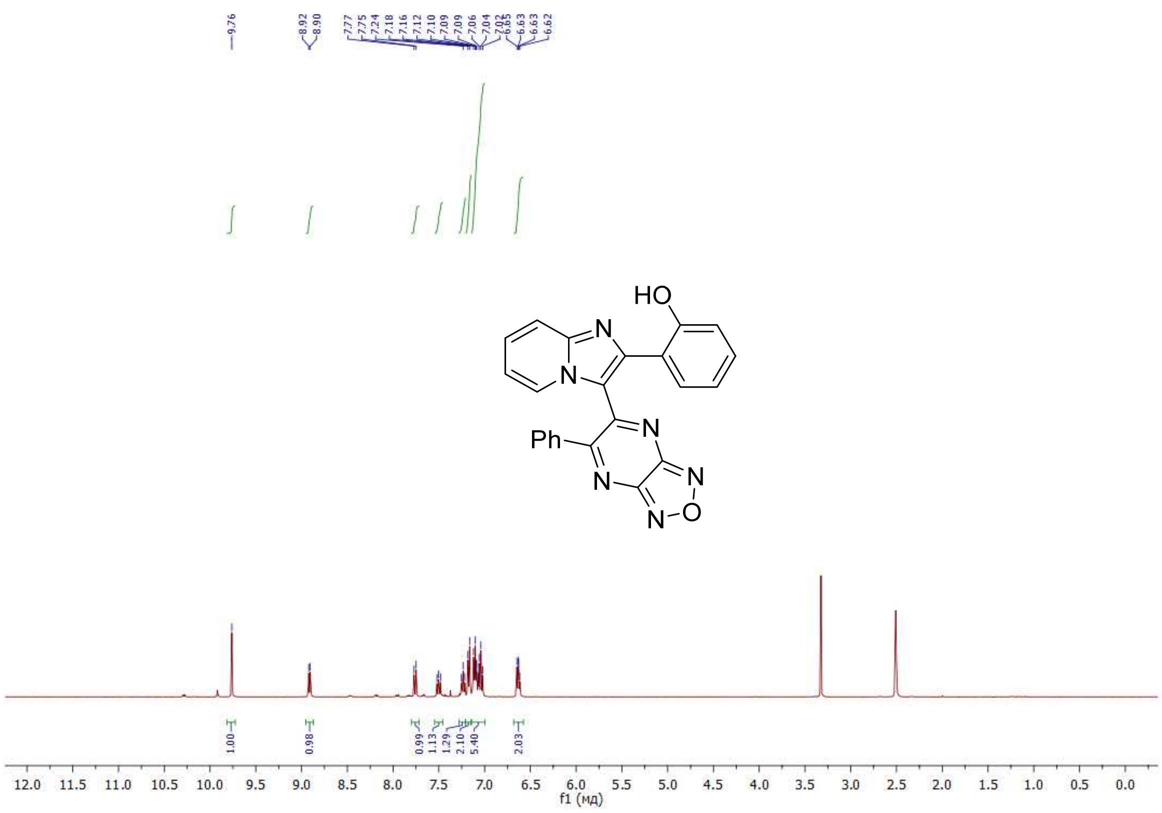

Figure S34. ^{1}H NMR spectrum of **3m**

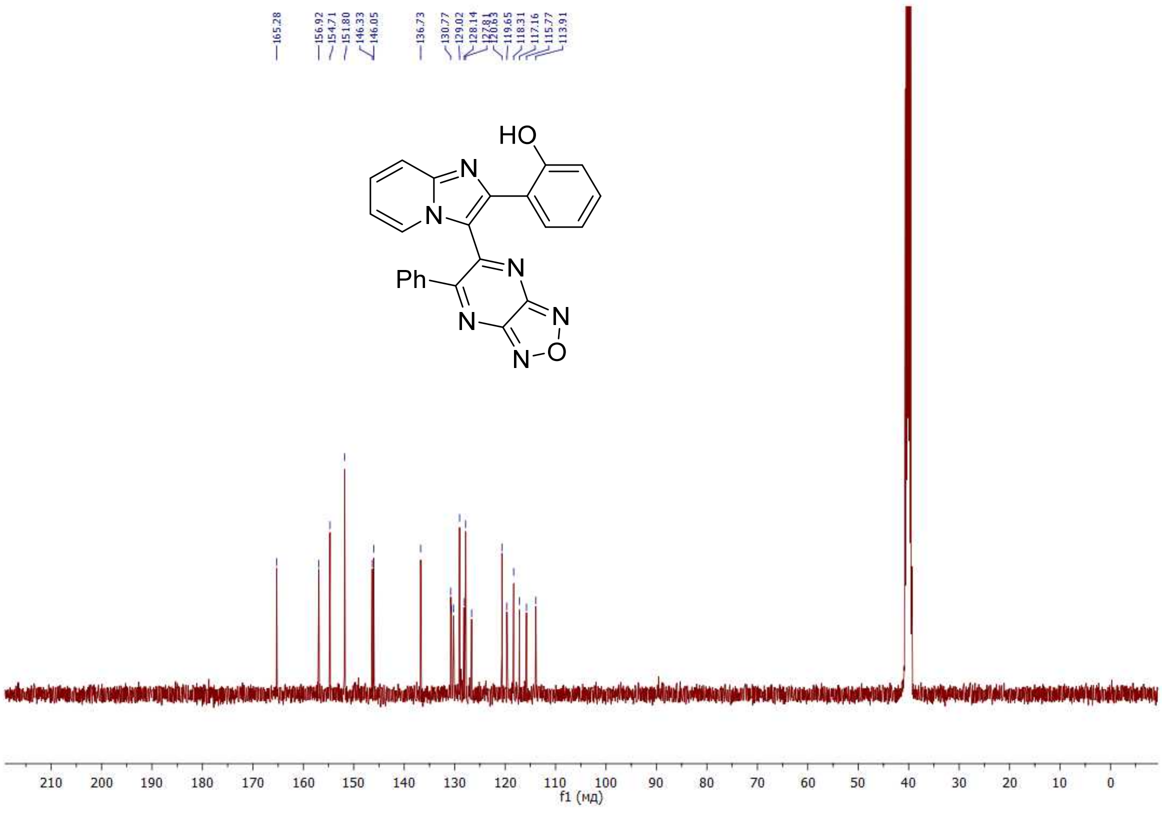

Figure S35. ^{13}C NMR spectrum of **3m**

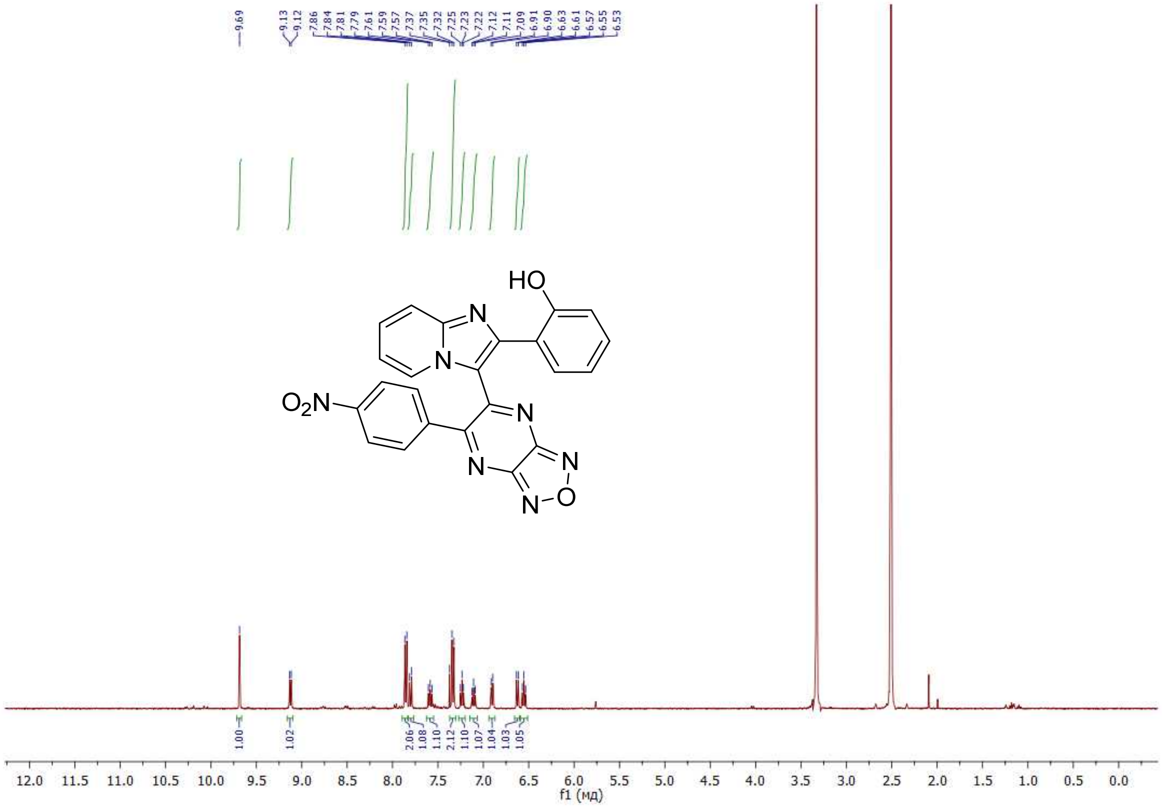

Figure S36. ¹H NMR spectrum of **3n**

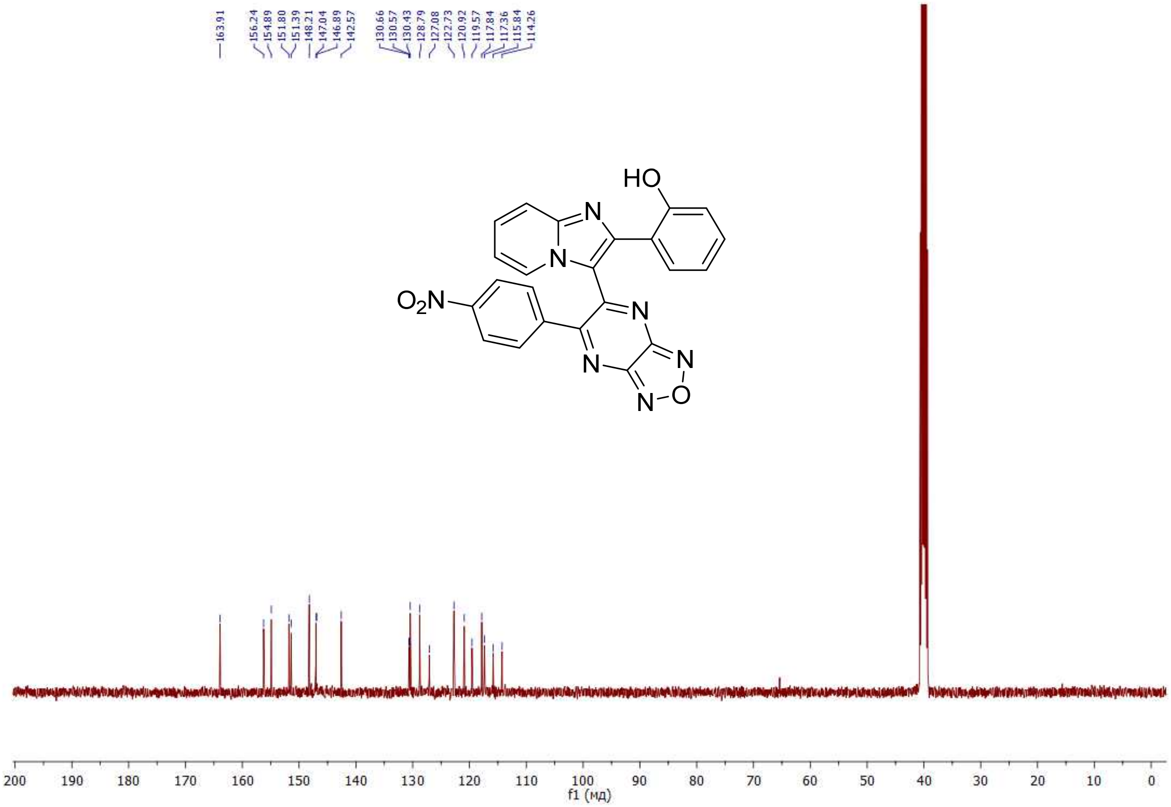

Figure S37. ¹³C NMR spectrum of **3n**

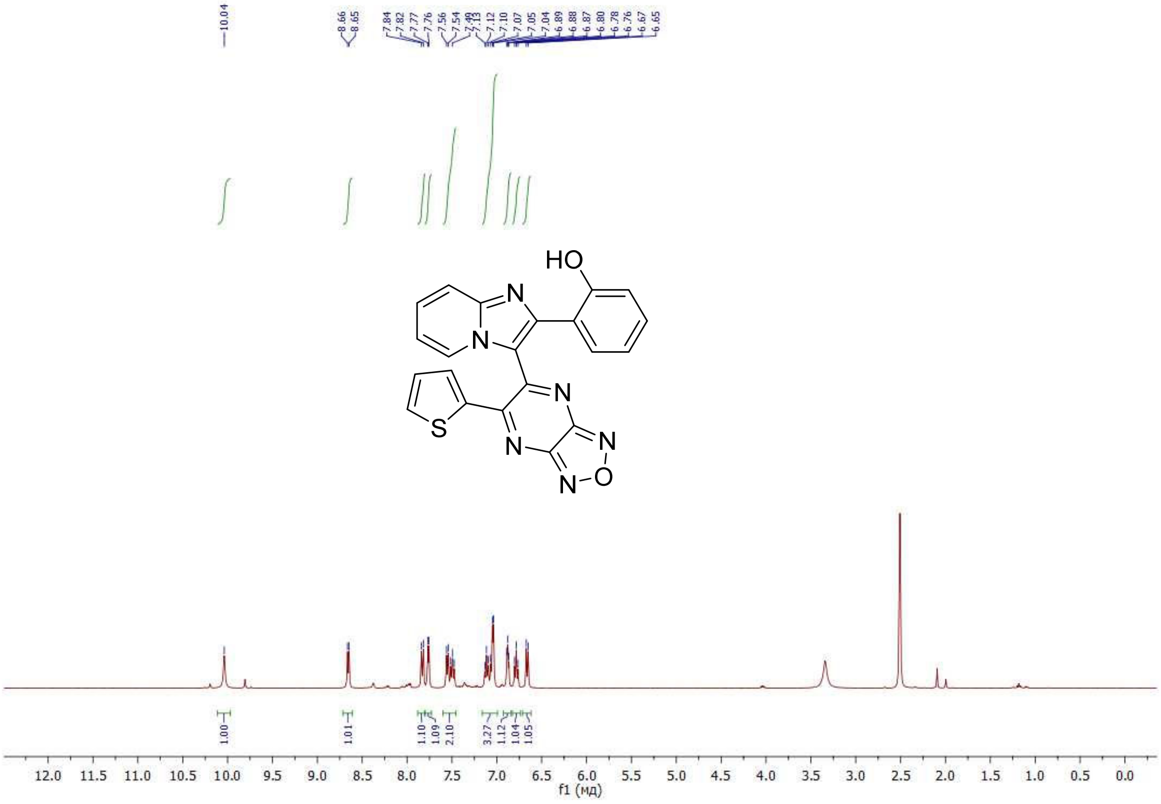

Figure S38. ^{1}H NMR spectrum of **3o**

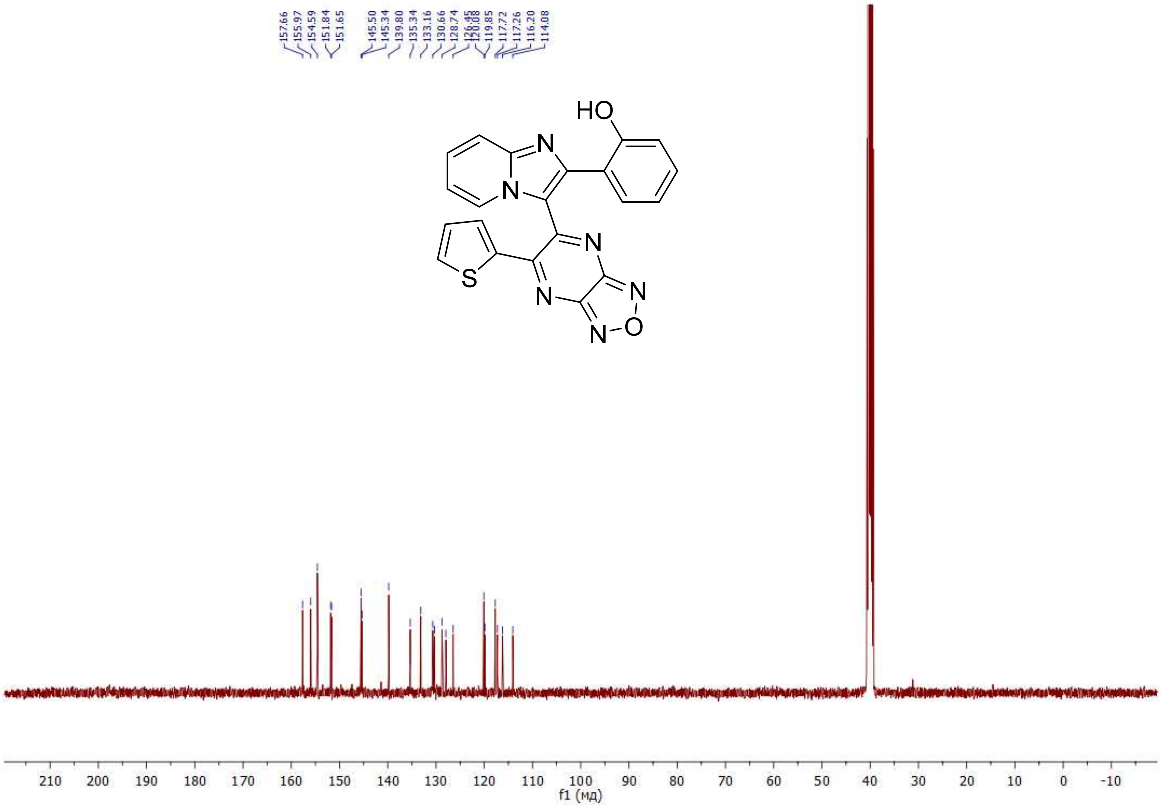

Figure S39. ^{13}C NMR spectrum of **3o**

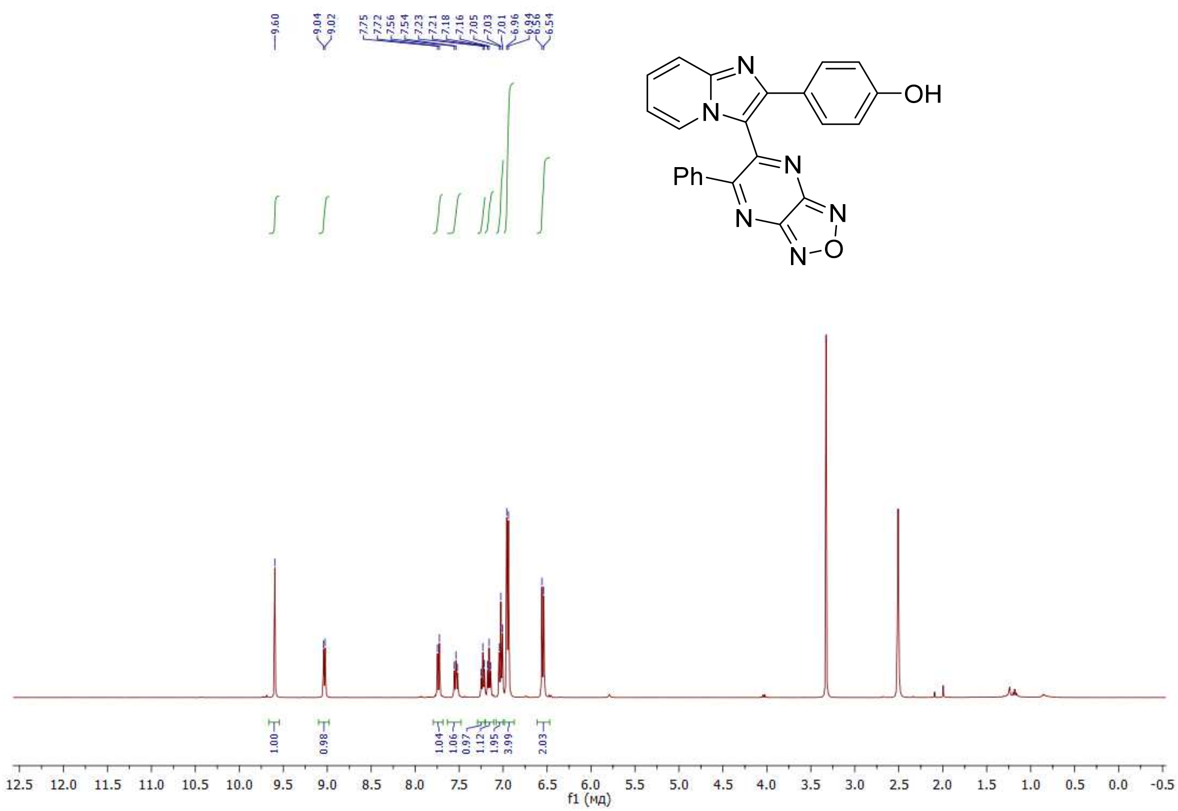

Figure S40. ^{1}H NMR spectrum of **3p**

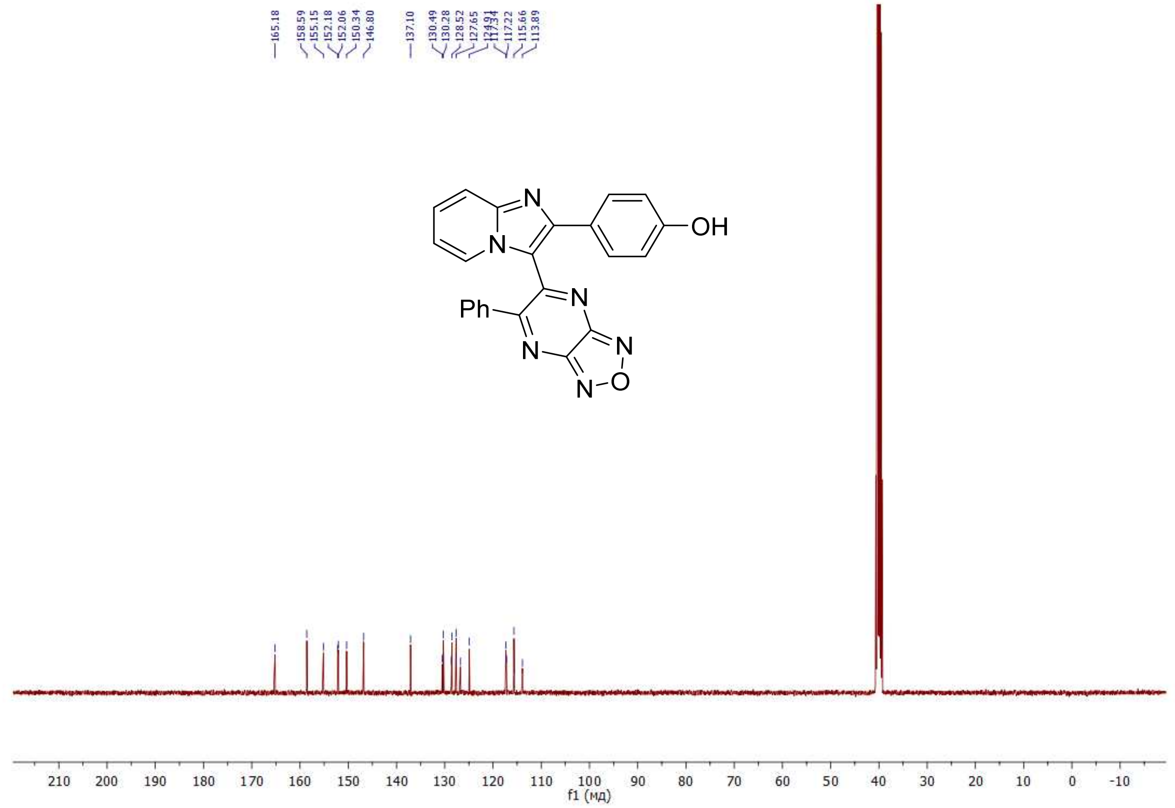

Figure S41. ^{13}C NMR spectrum of **3p**

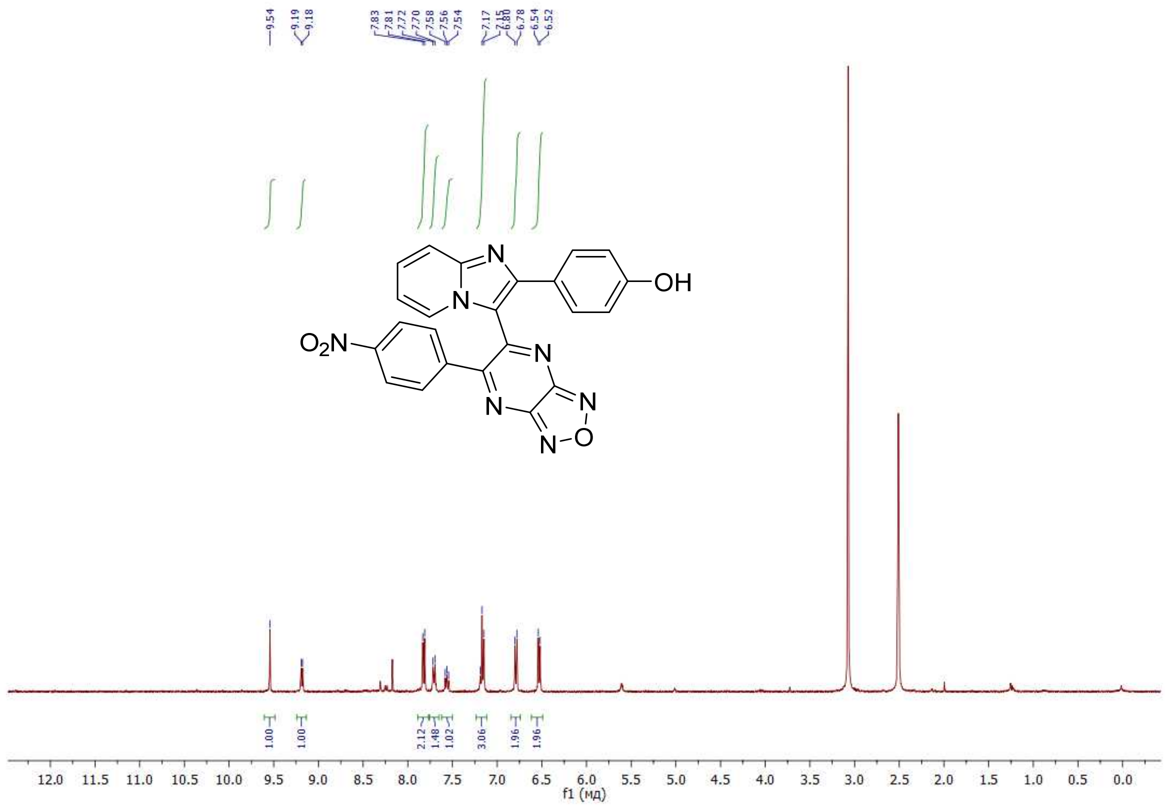

Figure S42. ^{1}H NMR spectrum of **3q**

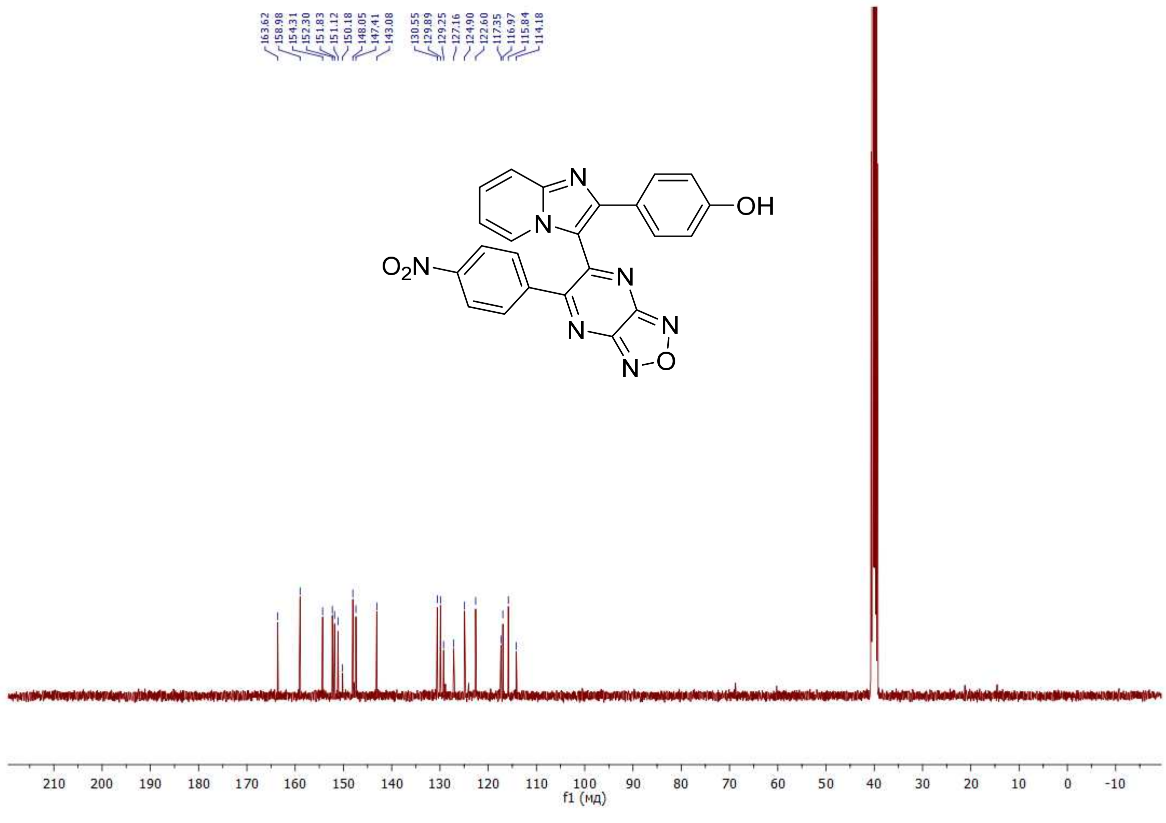

Figure S43. ^{13}C NMR spectrum of **3q**

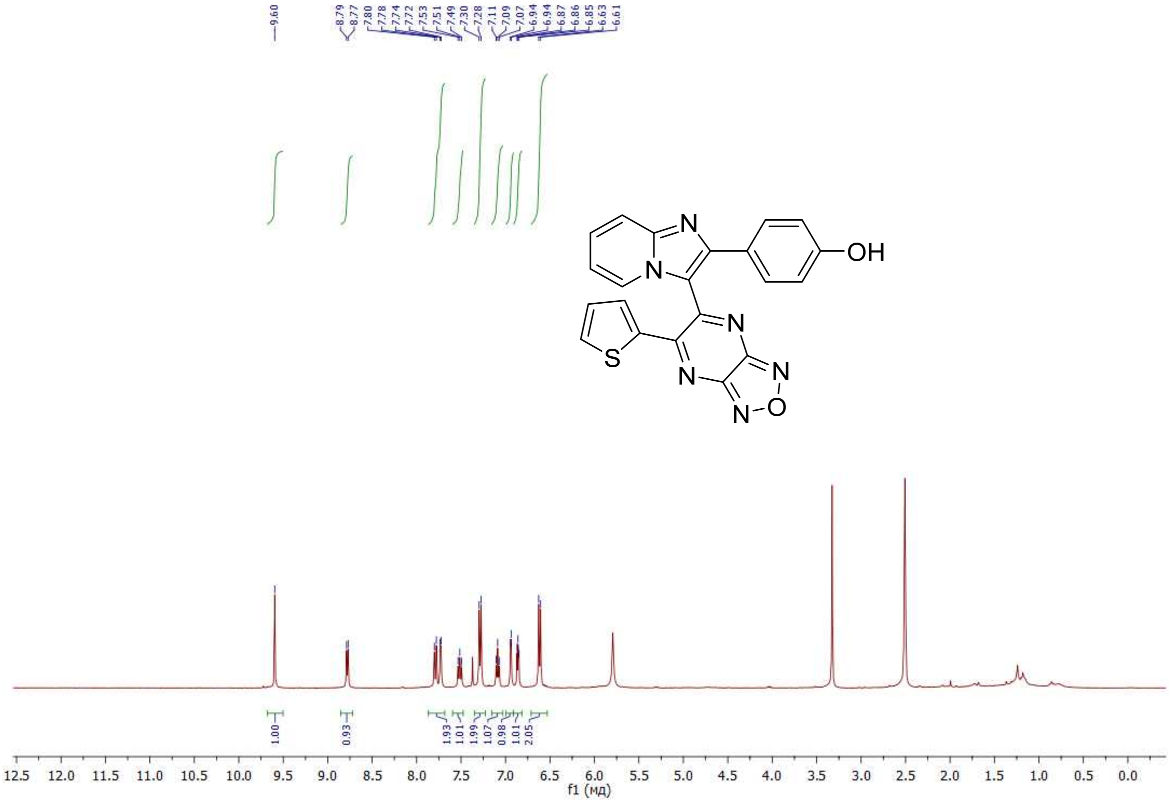

Figure S44. ^{1}H NMR spectrum of **3r**

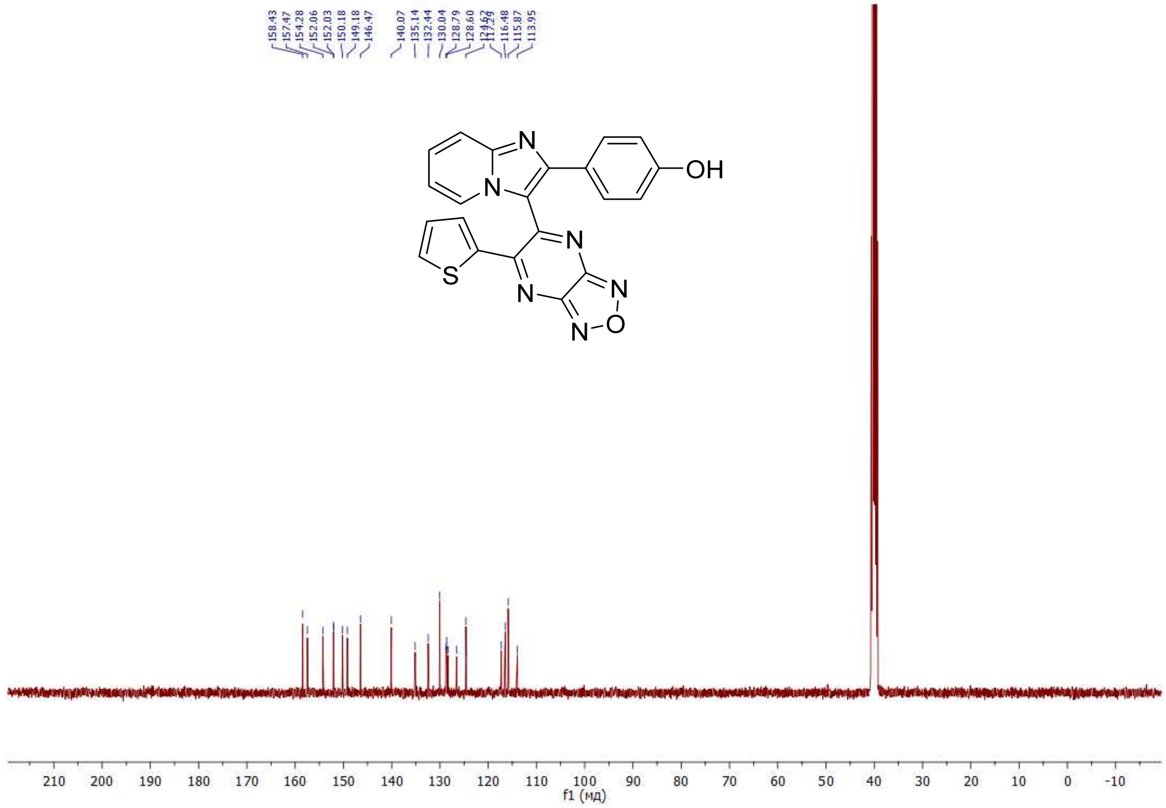

Figure S45. ^{13}C NMR spectrum of **3r**

4. Single-crystal X-ray diffraction analysis the structure of **3l**

Table S1. Selected distances and angles **3l**

Fragment	Distances, Å	Angles, deg.
oxadiazol	O-N 1.388(2), 1.397(2)	N-O-N 113.00(14)
	C-N 1.320(3) , 1.321(2)	C-N-O 103.43(16), 103.75(16)
	C-C 1.424(3)	C-C-N 109.95(17), 109.87(17)
pyrazine	C-N 1.319(2)-1.371(2)	C-N-C 113.28(15), 113.82(15)
	C-C 1.424(3), 1.506(2)	C-C-N 122.12(16)-123.38(17)
pyridine	C-N 1.382(2), 1.395(2)	C-N-C 122.52(15)
	C-C 1.366(3)-1.426(3)	C-C-N 118.59(17), 118.67(17)
		C-C-C 119.02(17)-120.80(17)
imidazol	C-N 1.343(2)-1.398(2)	N-C-N 111.39(15)
	C-C 1.394(2)	C-N-C 105.48(14) 106.37(15)
		C-C-N 105.09(15), 111.61(15)
F-phenyl	C-C 1.375(3)-1.396(3)	C-C-C 118.5(2)-120.52(19)
	C-F 1.372(2)	C-C-F 118.1(2), 118.7(2)
thiophene	S-C 1.697(2), 1.732(2)	S-C-C 112.23(14), 112.74(16)
	C-C 1.379(3)-1.435(3)	C-S-C 91.81(11)
		C-C-C 109.61(18)-130.09(17)

Table S2. Parameters of C-H…π interaction in **3l** (Cg_i is centroid of aromatic ring; H-Perp is perpendicular distance of X on ring; γ is angle X→Cg_i vector and normal to ring plane)

C-H...Cg_i	Symmetry element	H..Cg, Å	H-Perp, Å	γ, deg.	C-H..Cg, deg.	C..Cg, Å
C21-H21… C_{g3}(N1C5N2C6C7)	x,y,z	2.89	2.65	23.82	118	3.431(3))

Table S3. Selected parameters for π-π interactions in **3l** (C_g-C_g is a distance between ring centroids (Å); α is a dihedral angle between planes I and J (deg.); C_g(I)-Perp is perpendicular distance of C_g(I) on ring J (Å); C_g(J)-Perp is perpendicular distance of C_g(J) on ring I (Å); Slippage is a distance between C_g(I) and perpendicular projection of C_g(J) on ring I (Å)).

Rings I and J	Symmetry element	C_g-C_g	α	C_g-Perp	Slippage

C_{g1}(S1C18C20C21C19)…C_{g6} (C8-C13)	x,y,z	3.658(3)	19.06(10)	3.4136(8)	1.080
C_{g2}(O1N5C15C16N6)…C_{g3}(N1C5N2C6C7)	x,1/2-y,1/2+z	3.624(3)	2.18(11)	3.5762(9)	0.710
C_{g2}(O1N5C15C16N6)…C_{g4}(N1C1-C5)	x,3/2-y,-1/2+z	3.872(3)	4.42(11)	3.5097(9)	1.593
C_{g4}(N1C1-C5)…C_{g5}(N3C14C17N4C16 C15)	x,3/2-y,-1/2+z	3.694(3)	1.37(9)	3.4823(8)	1.311

Table S4. Selected parameters for the C–H···N interactions in **3l**

Interaction	Symmetry element	C–H, Å	H···N, Å	C···N, Å	C–H–N, deg.
C4-H4...N6	x,y,-1+z	0.93	2.62	3.456(4)	149
